# Diversification of phenolic glucosides by two UDP-glucosyltransferases featuring complementary regioselectivity

**DOI:** 10.1186/s12934-022-01935-w

**Published:** 2022-10-10

**Authors:** Fei Guo, Xingwang Zhang, Cai You, Chengjie Zhang, Fengwei Li, Nan Li, Yuwei Xia, Mingyu Liu, Zetian Qiu, Xianliang Zheng, Li Ma, Gang Zhang, Lianzhong Luo, Fei Cao, Yingang Feng, Guang-Rong Zhao, Wei Zhang, Shengying Li, Lei Du

**Affiliations:** 1 State Key Laboratory of Microbial Technology, Shandong University, Qingdao, 266237 Shandong China; 2 Shenzhen Branch, Guangdong Laboratory for Lingnan Modern Agriculture, Genome Analysis Laboratory of the Ministry of Agriculture, Agricultural Genomics Institute at Shenzhen, Chinese Academy of Agricultural Sciences, Shenzhen, 518000 China; 3 Shandong Provincial Key Laboratory of Synthetic Biology, CAS Key Laboratory of Biofuels, Qingdao Institute of Bioenergy and Bioprocess Technology, Chinese Academy of Sciences, Qingdao, 266101 Shandong China; 4 Frontier Science Center for Synthetic Biology and Key Laboratory of Systems Bioengineering (Ministry of Education), School of Chemical Engineering and Technology, Tianjin University, Yaguan Road 135, Jinnan District, Tianjin, 300350 China; 5 Key Laboratory of Marine Drugs, Ministry of Education of China, School of Medicine and Pharmacy, Ocean University of China, Qingdao, 266003 Shandong China; 6 College of Biotechnology, Tianjin University of Science and Technology, Tianjin, 300457 China; 7 Fujian Universities and Colleges Engineering Research Center of Marine Biopharmaceutical Resources, Xiamen Medical College, Xiamen, 361023 Fujian China; 8 College of Pharmaceutical Sciences, Hebei University, Baoding, 071002 China; 9 Laboratory for Marine Biology and Biotechnology, Qingdao National Laboratory for Marine Science and Technology, Qingdao, 266237 Shandong China; 10 Center For Biocatalysis and Enzyme Technology, Angel Yeast Co., LTD, Cheng Dong Avenue, Yichang, 443003 Hubei China

**Keywords:** Phenolic glucosides, Biocatalysis, UDP-glucosyltransferases, Alkylphenol bio-oxidation

## Abstract

**Background:**

Glucoside natural products have been showing great medicinal values and potentials. However, the production of glucosides by plant extraction, chemical synthesis, and traditional biotransformation is insufficient to meet the fast-growing pharmaceutical demands. Microbial synthetic biology offers promising strategies for synthesis and diversification of plant glycosides.

**Results:**

In this study, the two efficient UDP-glucosyltransferases (UGTs) (UGT85A1 and RrUGT3) of plant origin, that are capable of recognizing phenolic aglycons, are characterized in vitro. The two UGTs show complementary regioselectivity towards the alcoholic and phenolic hydroxyl groups on phenolic substrates. By combining a developed alkylphenol bio-oxidation system and these UGTs, twenty-four phenolic glucosides are enzymatically synthesized from readily accessible alkylphenol substrates. Based on the bio-oxidation and glycosylation systems, a number of microbial cell factories are constructed and applied to biotransformation, giving rise to a variety of plant and plant-like *O*-glucosides. Remarkably, several unnatural *O*-glucosides prepared by the two UGTs demonstrate better prolyl endopeptidase inhibitory and/or anti-inflammatory activities than those of the clinically used glucosidic drugs including gastrodin, salidroside and helicid. Furthermore, the two UGTs are also able to catalyze the formation of *N*- and *S*-glucosidic bonds to produce *N*- and *S*-glucosides.

**Conclusions:**

Two highly efficient UGTs, UGT85A1 and RrUGT3, with distinct regioselectivity were characterized in this study. A group of plant and plant-like glucosides were efficiently synthesized by cell-based biotransformation using a developed alkylphenol bio-oxidation system and these two UGTs. Many of the *O*-glucosides exhibited better PEP inhibitory or anti-inflammatory activities than plant-origin glucoside drugs, showing significant potentials for new glucosidic drug development.

**Supplementary Information:**

The online version contains supplementary material available at 10.1186/s12934-022-01935-w.

## Background

**Fig. 1:**
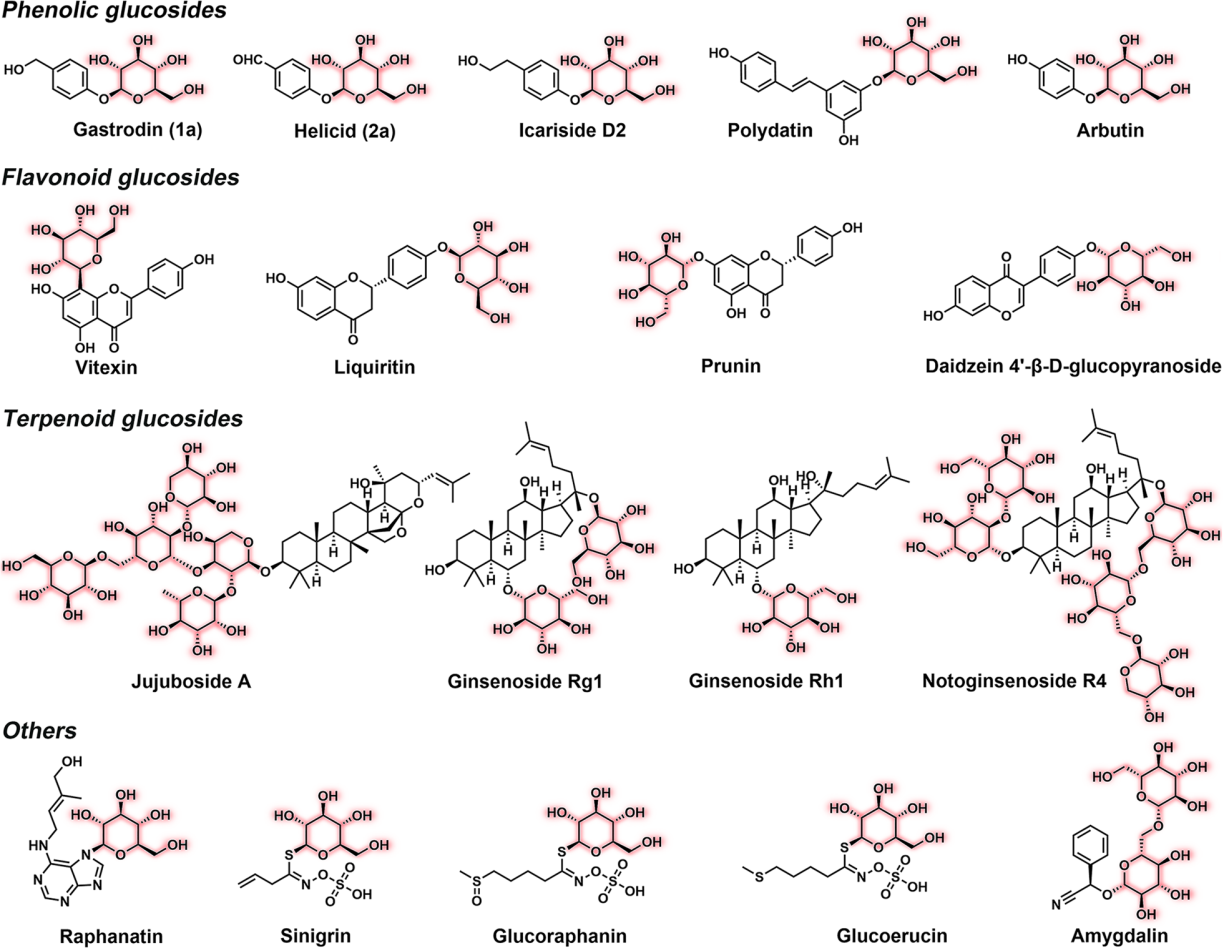
Representative phenolic, flavonoid, terpenoid glucosides and others

Glucosides (Fig. 1) comprised of non-sugar moiety (i.e., aglycon) and one or more sugars connected by the glucosidic bond are broadly distributed in plants [1]. According to the aglycon structures, the plant-derived glucosides mainly include flavonoid, terpenoid, phenolic glucosides, and others [2]. Among this important family of plant natural products, phenolic glucosides isolated from medicinal herbs have been attracting growing interests in healthcare owing to their considerable bioactivities, with some having been used in clinics [3, 4]. For instance, salidroside, one of the major ingredients of the herb *Rhodiola rosea*, exhibits significant therapeutic effects by protecting nerve and brain cells, thus being used for the treatment of binge eating [5, 6] and preventing damage from ischemia [7]. Gastrodin with *p*-hydroxybenzyl alcohol (**1**) as aglycon, originating from the traditional Chinese medicinal herb *Gastrodia elata*, has potent anti-inflammatory and antioxidation activities and can inhibit apoptosis pathways to alleviate brain hypoxia injury [8]. Helicid with *p*-hydroxybenzaldehyde (**2**) as aglycon, the main active constituent from the seeds of the medicinal plant *Helicia nilagirica*, has been reported to show sedative, analgesic, and hypnotic effects [9, 10]. Owing to these important bioactivities, these phenolic glucosides have been used as over-the-counter (OTC) drugs to treat neurasthenic syndrome, vascular headaches, and trigeminal neuralgia.

The structural variety of aglycons dramatically expands the diversity of biological activities of glucosides. According to the type of glucosidic bonds, naturally occurring glucosides are divided into four categories including *O*-, *N*-, *C*-, and *S*-glucosides [11], among which *O*-glucosides represent the most abundant and important subfamily for biochemical, pharmaceutical and biomedical researches and applications [12]. Currently, almost all therapeutic *O*-glucosides are plant-extracted or chemically synthesized. However, due to insufficient herb resources, natural glucosides often cannot meet the fast-increasing demands. As to chemical synthesis, it usually requires tedious protection and deprotection steps to achieve glucosylation, thus leading to high cost and low yield [13]. Moreover, the chemical approaches are generally restricted by poor regio- and stereoselectivity; and a variety of toxic reagents are often required, leading to serious environmental concerns [14]. Taken together, the preparation of variant *O*-glucosides in a sustainable, cost-effective, and environment-friendly manner is urgently demanded, but remains challenging.

In the era of synthetic biology, a growing number of biosynthetic systems for *O*-glucosides based on in vitro enzymatic cascades and in vivo metabolic engineering have been developed. For example, the microbial cell factories for salidroside, gastrodin, and icariside D2 have been constructed by integrating different uridine diphosphate (UDP) glucosyltransferases (UGT) and the fine-tuned aglycon supplying pathways [15–18]. Various biocatalytic systems for unnatural *C*-glucosides [19, 20] and *O*-glucosides [21–23] have also been established by taking advantage of the substrate promiscuity of UGTs. However, the previous studies have been focused on product titer improvement for natural *O*-glucosides. With regard to unnatural *O*-glucosides, the scope of aglycons has been limited to flavonoids and terpenoids. The use of alkylphenol derivatives as aglycons for systematic biosynthesis of unnatural *O*-glucosides is rarely reported.

Aiming to diversify the structures of phenolic *O*-glucosides to provide more drug candidates, in this study, we characterized two efficient UGTs with complementary chemo- and regioselectivity including UGT85A1 [15] from *Arabidopsis thaliana* for glucosylation of the alcoholic hydroxyl group and RrUGT3 [24] from *R. rosea* for glucosylation of the phenolic hydroxyl group. Taking advantage of an alkylphenol bio-oxidation system and these two UGTs, twenty-four phenolic *O*-glucosides, as well as two *N*- and three *S*-glucosides, were biosynthesized by a number of engineered microbial cell factories. Several unnatural glucosides demonstrated significant prolyl endopeptidase inhibitory and anti-inflammatory activities, showing significant application potentials.

## Results

### Screening and functional characterization of candidate UGTs

Some UGTs can tolerate a broad spectrum of substrates, thus having been used as biocatalysts for production of various glucosylated derivatives [21, 25]. To gain UGTs with a high activity for glucosylation of phenolic aglycons, we compared eight UGTs from plants or bacteria, which were previously identified to have considerable substrate promiscuity and catalytic efficiency towards phenolic substrates. These UGTs include: UGT85A1 from *A. thaliana* and UGT72B14 from *Rhodiola sachalinensis*, applied to produce salidroside [15, 26]; RrUGT3 from *R. rosea*, reported for efficient production of icariside D2 [18]; RrUGT33 and RrUGT17 from *R. rosea*, characterized as the key UGTs for biosynthesis of tyrosol glucosides in *Rhodiola* genus [24]; UGT73B6 from *R. sachalinensis* and its mutant UGT73B6F389S (UGT73B6FS), used in synthesis of many aromatic glucosides [27]; and YjiC from *Bacillus licheniformis*, having the capability to synthesize many types of glucosides [15, 28].

To improve the heterologous expression in *Escherichia coli*, all the eight UGT genes were synthesized with their codon-optimized accordingly. These codon-optimized genes were sub-cloned into the plasmid pET28b with appropriate primers (Additional file 1: Table S1). Then, the recombinant expression vectors were individually transformed into *E. coli* BL21(DE3) for protein expression (Additional file 1: Table S2). Upon induction and over-expression, the *N*-terminal His_6_-tagged UGT proteins were purified to homogeneity using Ni–NTA agarose resin. Specifically, six (UGT85A1, RrUGT3, RrUGT17, UGT73B6, UGT73B6FS and UGTYjiC) out of eight UGTs were well purified, while the rest two UGTs (RrUGT33 and UGT72B14) were mostly distributed in precipitation with only low concentrations of soluble proteins in supernatant (Additional file 1: Fig. S1).

**Fig. 2:**
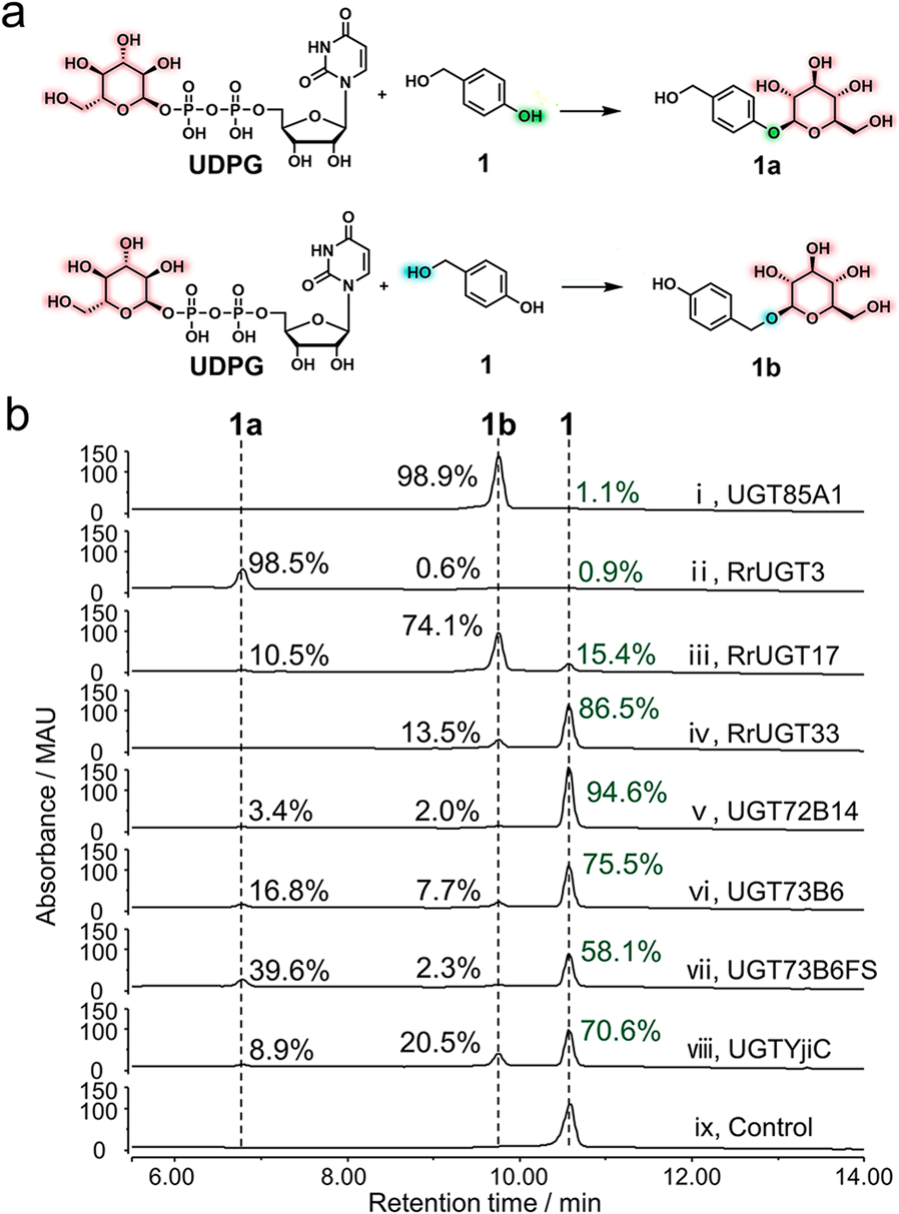
Screening of candidate UGTs. **a** The reaction schemes for glucosylation of *p*-hydroxybenzyl alcohol (**1**). **b** HPLC analysis of the glucosylation reactions catalyzed by eight UGTs, individually, with UDP-glucose as sugar donor and **1** as sugar receptor (aglycon). i–viii: Reactions with different UGTs; ix: Control without enzyme. The percentage numbers in green represent the ratio of unreacted substrates

We first used *p*-hydroxybenzyl alcohol (**1**), which possesses both phenolic and alcoholic hydroxyl groups to be potentially glucosylated, as a representative substrate to screen the glucosylation activity of the above-mentioned eight UGTs. With UDP-glucose (UDPG) as the sugar donor, the in vitro glucosylation reactions mediated by each UGT were carried out at 30 °C for 2 h. As a result, all the eight UGTs exhibited varying glucosylation activities towards **1** with the conversion ratios ranging from 5.4 to 99.1% (Fig. 2). Comparatively, UGT85A1, RrUGT3 and RrUGT17 showed higher activities than other UGTs with the conversion ratios of 98.9%, 99.1% and 84.6%, respectively (Fig. 2b traces i, ii and iii). Of note, the three UGTs demonstrated distinct regioselectivity towards **1**. Specifically, RrUGT3 predominantly glucosylated the phenolic hydroxyl group of **1** to generate the glucoside **1a** as the main product (Fig. 2b trace ii). By contrast, UGT85A1 and RrUGT17 favored the alcoholic hydroxyl of **1**, while UGT85A1 showed higher selectivity than RrUGT17, producing only the alcoholic glucoside **1b** (Fig. 2b traces i and iii). Considering the complementary regiospecific glucosylation patterns and the high activity of RrUGT3 and UGT85A1, these two UGTs were selected for the subsequent production of glucosides.

### Diversification of phenolic aglycons

**Fig. 3:**
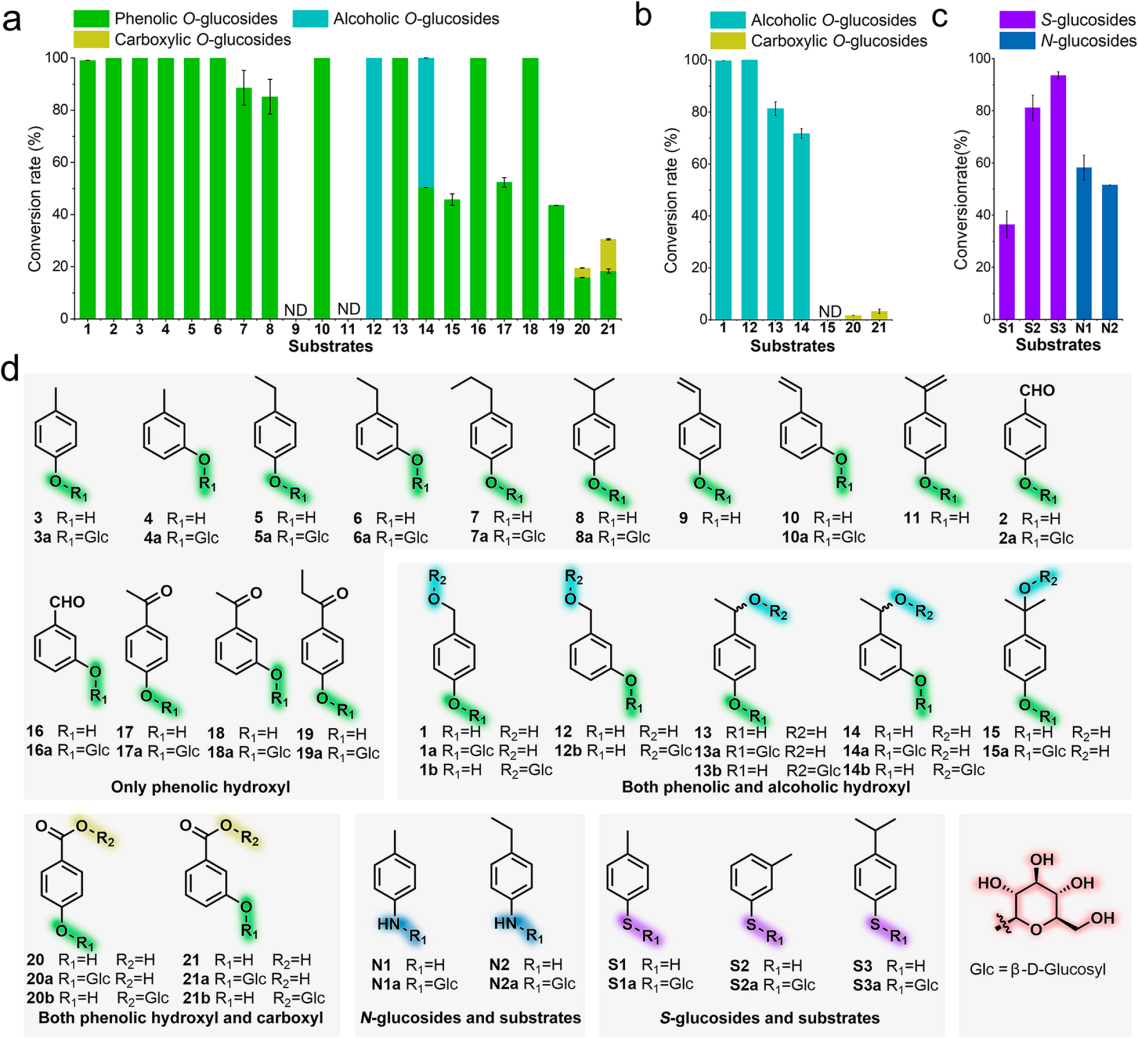
Substrate promiscuity of RrUGT3 and UGT85A1. **a** Conversions of different aglycons by RrUGT3. **b** Conversions of different aglycons by UGT85A1. **c** Conversions of *N*- and *S*-aglycons by RrUGT3. **d** Structures of substrates and their corresponding glucosylated products. *ND* no detected

Alkylphenols and their oxidative derivatives (with modifications on the alkyl group) are important aglycons for the biosynthesis of plant bioactive *O*-glucoside drugs (*e.g.*, gastrodin, salidroside and helicid) [17, 24, 29] or active pharmaceutical ingredients [30]. In general, alkylphenols are readily accessible chemicals; however, many of their oxidative derivatives are commercially unavailable or very expensive. Recently, we developed a unique “chemomimetic” bio-oxidation system [31], which is evolved from the *p*-cresol biodegradation pathway of the Gram-positive bacterium *Corynebacterium glutamicum*. This system could be used to efficiently and selectively functionalize the aliphatic C-H bonds in a range of *p*- and *m*-alkylphenols (Additional file 1: Fig. S2), providing a practical approach for generating alkyl-oxidized phenolic aglycons. Thus, in this study we prepared twelve oxidized derivatives (**1**, **2** and **12**–**21**) starting from nine commercially available alkylphenols (**3**–**11**) using this biocatalytic system (Fig. 3d). Structurally, compounds **2**, **3**–**11** and **16**–**19** bear only the phenolic hydroxyl group; while some of the oxidative derivatives possessing both phenolic and alcoholic hydroxyl groups (**1** and **12**–**15**) or carboxyl groups (**20** and **21**). All these twenty-one compounds constitute a potential library providing diverse phenolic aglycons for investigating and biosynthesis of plant and plant-like *O*-glucosides.

### Biosynthesis of glucosides based on the substrate promiscuity of RrUGT3 and UGT85A1

The library consisting of twenty-one phenolic compounds (**1**–**21**) were used as the aglycons to investigate the substrate promiscuity and regioselectivity of RrUGT3 and UGT85A1 (Fig. 3). To avoid using the expensive nucleotide sugar donor UDPG and to facilitate the production of glucoside products in large quantities, we established an eco-friendly and cost-effective in vivo biotransformation approach using the *E. coli* BL21(DE3) strains that heterologously express RrUGT3 or UGT85A1.

We first used the engineered *E. coli* BL21(DE3) strain harboring pCDFDuet-1-*RrUGT3* (named as GF-1, Additional file 1: Table S2) to transform **1**–**21** individually. Considering that these small phenolic compounds should be able to pass through the bacterial membrane, the substrates were directly fed into the GF-1 cultures for biotransformation. Except for **9** and **11**, which were not stable and precipitated upon substrate feeding, all the rest phenolic substrates were glucosylated by RrUGT3 (Fig. 3a, d) with the conversion ratios ranging from 19.5% to 100.0% (more than half of compounds were completely converted into the corresponding glucosides, Additional file 1: Figs. S3–S21). Structure elucidation of the purified products by high-resolution mass spectrometry (HRMS) (Additional file 1: Fig. S22) and nuclear magnetic resonance (NMR) spectroscopy (Additional file 1: Figs. S23–S63) showed that a majority of products (**1a**–**8a**, **10a**, **13a**–**21a**) contained the expected phenolic *O*-glucosidic bond. However, for the substrate **14** containing a benzylic hydroxyl group at *m*-position, RrUGT3 lost the selectivity as the two products (**14a** and **14b**) respectively containing a phenolic or alcoholic *O*-glucosidic bond were almost equally generated (Fig. 3a, d and Additional file 1: Figs. S14, S22, S44–S45, S64–S65). Strikingly, the selectivity of RrUGT3 was completely reversed for **12** (Fig. 3a, d and Additional file 1: Figs. S12, S22, S66–S70), which has a similar *m*-alcoholic hydroxyl group as **14**. Moreover, RrUGT3 was able to transfer a glucose moiety to the carboxyl group of **20** and **21**, giving rise to the glucosyl ester products (**20b** and **21b**) (Fig. 3a, d and Additional file 1: Figs. S20–S22, S71–S80). Taken together, these results indicate that, although RrUGT3 prefers the phenolic hydroxyl group, the position of the potentially reactive alcoholic hydroxyl group might deceive RrUGT3, thus changing the chemo- or regioselectivity of this enzyme.

Considering that UGT85A1 could specifically recognize alcoholic hydroxyl group in the screening assays (Fig. 2), the *E. coli* BL21(DE3) strain expressing UGT85A1 (named as GF-2, Additional file 1: Table S2) was next utilized for biotransformation of the substrates containing an alcoholic hydroxyl group (**1**, **12**–**15**) or carboxyl group (**20**, **21**). Except **15**, UGT85A1 converted all the tested substrates with conversion ratios ranging from 1.7% to 100.0% (Fig. 3b, d and Additional file 1: Figs. S81–S86). Structurally, the products of **1** and **12**–**14** were all alcoholic *O*-glucosides (Fig. 3b, d and Additional file 1: Figs. S22, S64–S70, S87–S96). The conversion rate of **14** (71.8%) was slightly lower than that of **13** (81.4%), suggesting that the substitution at the *m-*position of substrate might have a negative effect on the catalytic efficiency of UGT85A1. For compounds **20** and **21** with a carboxyl group, UGT85A1 exhibited weak catalytic activities, but exclusively yielded the ester glucosides (**20b** and **21b**) (Fig. 3b, d and Additional file 1: Figs. S22, S71–S80, S85–S86). Of note, UGT85A1 did not show any activity to the phenolic hydroxyl group of these testing substrates since no products with a phenolic glucosidic bond were detected. In addition, the in vitro enzymatic assays of UGT85A1 showed very low conversion ratios (0–16.3%) towards the substrates with only one phenolic hydroxyl group (Additional file 1: Fig. S97). These results indicate that UGT85A1 exhibits high preference to the substrates with alcoholic hydroxyl group.

It is worth noting that all glucoside products are mainly distributed outside bacterial cells (Additional file 1: Figs. S3–S21, S81–S86) based on HPLC analysis of fermentation broth and cell extract. Due to the high polarity and aqueous solubility, the common organic extraction approach failed for these glucosides. Thus, we examined two types of macroporous resins (AB-8 and S-8) for product purification and found that the former performed better (Additional file 1: Fig. S98). Using the AB-8 resin, the glucoside products were enriched with their yields varying from 43.8 to 92.2% (Additional file 1: Table S3), and finally purified by preparative HPLC. As results, twenty-four *O*-glucosides were purified and structurally elucidated by HRMS (Additional file 1: Fig. S22) and NMR analyses (Additional file 1: Figs. S23–S80, S87–S96). Unsurprisingly, the structural analysis indicated the same *β*-stereochemistry of the conjugated glucose in all glucosides.

### Biosynthesis of glucosides through combining a developed alkylphenol oxidation system

As described above, the “chemomimetic” alkylphenol bio-oxidation system [31] provides an applicable approach for generating alkyl-oxidized phenolic aglycons. Thus, we sought to further establish an enzyme cascade to directly produce glucosides from more accessible alkylphenols (rather than the alkyl-oxidized phenolic compounds) by combining the alkylphenol oxidation and glucosylation systems.

In the bio-oxidation system, the aliphatic oxidation is mainly mediated by the core P450 biocatalyst CreJ. Since redox partner proteins have a great impact on the catalytic efficiency of P450, the endogenous redox partners CreE (ferredoxin reductase) and CreF (ferredoxin) were replaced by the surrogate but high-efficiency redox partners *Sel*Fdr0978 and *Sel*Fdx1499 from the cyanobacterium *Synechococcus elongatus* PCC 7942 [32–34], in order to improve the oxidation capacity of the alkylphenol oxidation system. Thus, the plasmid pRSFDuet-1-*CreH-CreI-CreJ-SelFdR0978-SelFdx1499-CreD* expressing all the proteins of the bio-oxidation system (CreH, CreI, CreJ, *Sel*FdR0978, *Sel*Fdx1499 and CreD) and the plasmid expressing an appropriate UGT gene (the plasmid pCDFDuet-1-*RrUGT3* for RrUGT3 or pCDFDuet-1-*UGT85A1* for UGT85A1) were co-transformed into *E. coli* BL21(DE3), yielding the strains GF-3 (containing the plasmids pRSFDuet-1-*CreH-CreI-CreJ-SelFdR0978-SelFdx1499-CreD* and pCDFDuet-1-*RrUGT3*) and GF-4 (containing the plasmids pRSFDuet-1-*CreH-CreI-CreJ-SelFdR0978-SelFdx1499-CreD* and pCDFDuet-1-*UGT85A1*) (Additional file 1: Table S2).

**Fig. 4:**
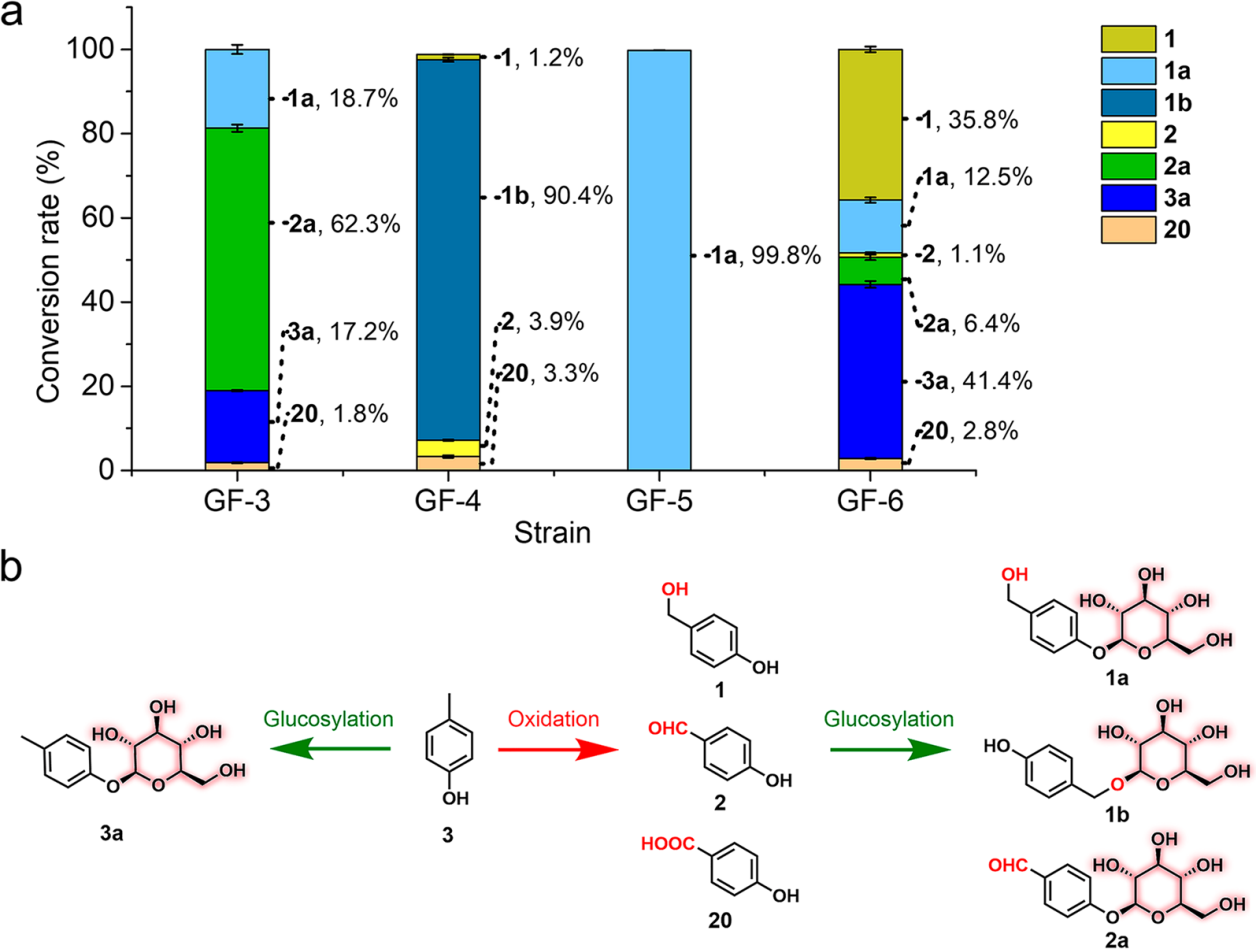
Biotransformation of **3** and **1** by different engineered *E. coli* strains. **a** Bioconversion rates. **b** Cascade biotransformation scheme

Considering that the oxidation products of **3** (*i.e.*, **1** and **2**) are the aglycons for the glucoside drugs gastrodin (**1a**) and helicid (**2a**), we chose **3** as an initial substrate to synthesize these bioactive glucosides. As expected, through fermentation and biotransformation in batch shaking flasks, the strain GF-3 (expressing RrUGT3) converted **3** into three glucosides including **1a**, **2a** and **3a** with the corresponding yields of 18.7%, 62.3% and 17.2% respectively (Fig. 4), indicating that the bio-oxidation and glucosylation cascade was successfully established. For the strain GF-4 (expressing UGT85A1), a major glucoside product **1b** (90.4% yield) was produced (Fig. 4). Notably, due to the low activity of UGT85A1 towards the carboxyl group, most of the end oxidation product **20** (3.3% yield) remained unglucosylated (Fig. 4).

The activities of glucosylation, as well as alkylphenol biotransformation of the strains GF-1 ~ 4, are dependent on their respective plasmids. To overcome the stability and heterogeneity problems associated with plasmids and eliminate the requirement of antibiotic addition, the strains with the bio-oxidation related genes and UGT gene integrated into the genome were further constructed. We first constructed the genome integration strain GF-5 (Additional file 1: Table S2), which is a counterpart of GF-1. In brief, taking advantage of a fast and robust iterative genome-editing CRISPR/Cas9 system based on the Rock-Paper-Scissors strategy [35], the gene encoding RrUGT3 was successfully integrated into the genome of *E. coli* BL21(DE3) at the *ushA* gene locus (in-frame deletion), knockout of which was reported to increase the supply of UDPG [15]. Upon feeding of **1**, GF-5 exhibited a similar activity (yield 99.8%) as the corresponding plasmid-based strain GF-1 (Fig. 4).

Motivated by this result, besides the *RrUGT3* gene, we further integrated all the genes including *CreH*, *CreI*, *CreJ*, *SelFdR0978*, *SelFdx1499* and *CreD* required for alkylphenol oxidation into the *ushA* locus (immediately downstream *RrUGT3*), giving rise to the strain GF-6 (Additional file 1: Table S2). Compared with its plasmid-based counterpart GF-3, GF-6 was also able to produce glucosides **1a** and **2a** from **3**, but with relatively lower efficiency (yields 12.5% and 6.4%, respectively), and certain amounts of unglucosylated compounds **1**, **2**, **20** remained (Fig. 4). The lower productivity might result from the limited protein expression levels due to the low copy number (only one copy) of genes in the genome-integration strain compared with the strain containing self-replicating plasmids with the multi-copy number (10 ~ 100).

### Evaluation of prolyl endopeptidase inhibitory and anti-inflammatory activities of glucosides

**Fig. 5:**
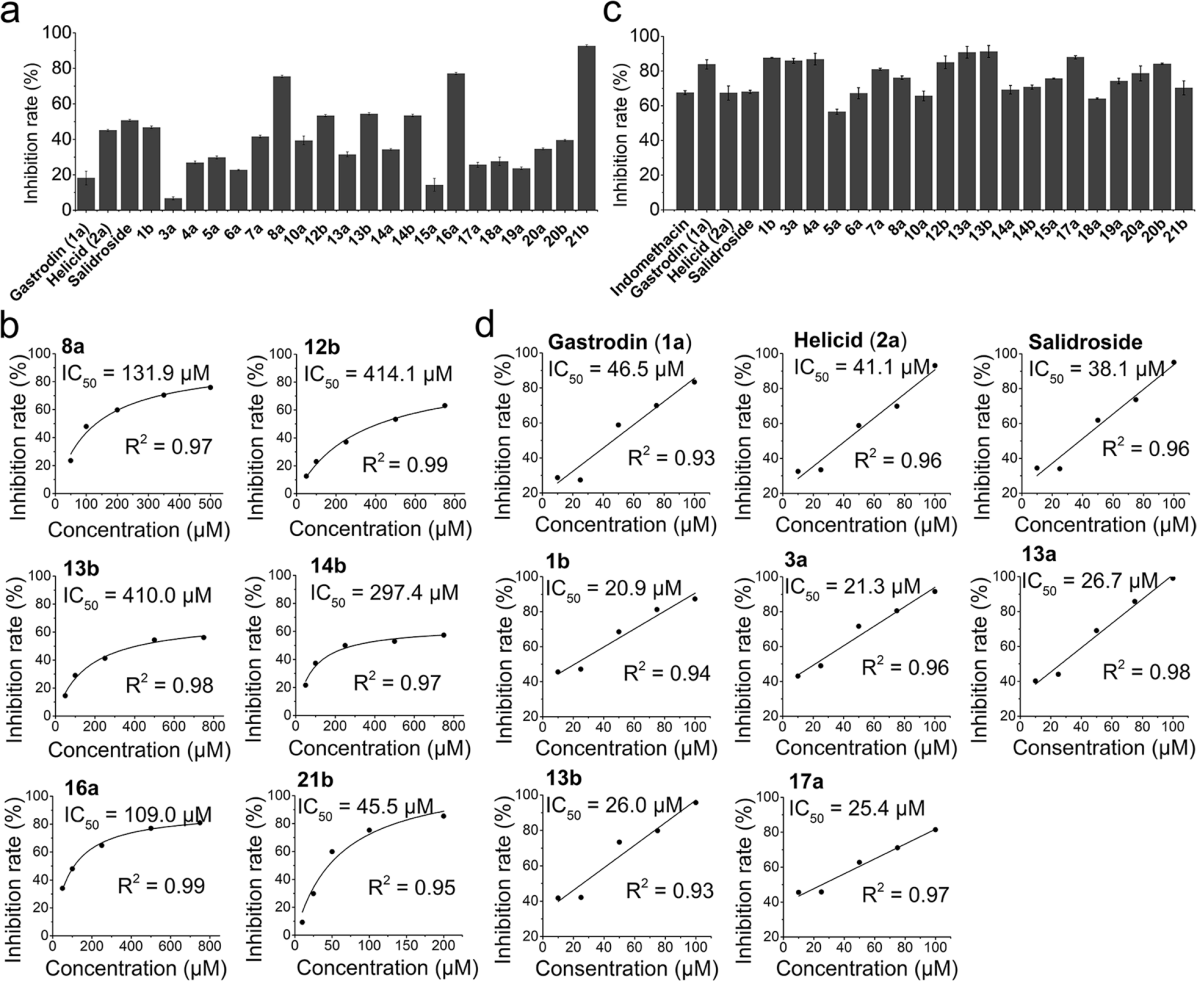
The PEP inhibitory and anti-inflammatory activities of glucosides. **a** PEP inhibitory rates of different glucosides. **b** Determination of the IC_50_ values of PEP inhibition. **c** Inhibition of NO release with the tested compounds. Indomethacin as positive control. **d** Determination of the IC_50_ values of anti-inflammatory activity

Prolyl endopeptidase (PEP) is a cytosolic enzyme involved in a number of important physiological processes such as degradation of certain peptide hormones and neuropeptides [36]. Some phenolic glucosides have been shown to demonstrate promising activities as PEP inhibitors [37–39]. Thus, we screened the PEP inhibitory activities of the glucosides biosynthesized in this study (Fig. 5a). Remarkably, six compounds (**8a**, **12b**, **13b**, **14b**, **16a** and **21b**) displayed a PEP inhibition rate of over 50% at 500 µM, and all of them showed better activities than those of typical drugs salidroside, gastrodin, and helicid. Glucoside **21b** exhibited the highest inhibitory activity (92.7%) with an IC_50_ value of 45.5 μM, while **8a** and **16a** displayed IC_50_ values of 131.9 and 109.0 μM, respectively (Fig. 5b).

The phenolic glucoside drugs gastrodin, salidroside, and helicid have extensively been reported to show anti-inflammatory activities [40–42]. The biosynthesized glycosidic products were further evaluated for their anti-inflammatory activities using the RAW 264.7 cell model [43]. We firstly examined the cytotoxicity of these glucosides towards human cells by CCK8 method, confirming that all of them were non-toxic (Fig. S99). Then following bioactivity assays were carried out. Based on the detection of inhibition rate of NO release, a number of the tested compounds, including **1b**, **3a**, **4a**, **12b**, **13a**, **13b**, **17a** and **20b** exhibited stronger NO release inhibitory activity than the anti-inflammatory drug indomethacin and the glucoside OTC drugs gastrodin, salidroside and helicid, at the concentration of 100 μM (Fig. 5c). Notably, glucosides **1b**, **3a**, **13a**, **13b** and **17a** showed much lower IC_50_ values (20.9, 21.3, 26.7, 26.0 and 25.4 µM, respectively) than those of gastrodin (**1a**, 46.5 µM), helicid (**2a**, 41.1 µM) and salidroside (38.1 µM) (Fig. 5d).

### Exploration of other activities of UGTs

The two UGTs, RrUGT3 and UGT85A1, exhibit high activity and selectivity for the phenolic and alcoholic hydroxyl group in phenolic aglycons, respectively. However, both enzymes are not good at glucosylating the carboxylic group. In order to efficiently synthesize carboxylic glucosides, alternative UGTs need to be screened [44]. For the two carboxylic compounds with low conversion efficiency, **20** and **21**, we re-screened our UGT library. Interestingly, UGT73B6FS showed much better catalytic activity for these two substrates than both RrUGT3 and UGT85A1, and the ester products **20b** and **21b** were significantly accumulated (yields 26.0% and 26.9%) (Additional file 1: Figs. S100–S101).

Besides catalyzing the formation of *O*-glycosidic bonds, some highly promiscuous UGTs also have the *S*- and *N*-glycosylation activities [20, 21, 45]. Among the tested substrates, RrUGT3 showed better aglycon promiscuity than UGT85A1. Thus, we further explored the possibility of using RrUGT3 to produce *S*-, and *N*-glucosides. The five selected aglycons were **S1**, **S2** and **S3** with a sulfhydryl group, and **N1** and **N2** with an amino group (Fig. 3d). To our delight, in vitro enzymatic reactions showed that all these aglycons could be converted by RrUGT3 with conversion ratios ranging from 66.3 to 88.2% (Additional file 1: Fig. S102). In order to obtain these glycoside products, we further carried out biotransformation experiments with strain GF-1 expressing RrUGT3. The results revealed that all the selected aglycons were efficiently transformed into the corresponding *S*-glucosides (**S1a**, **S2a** and **S3a**) and *N*-glucosides (**N1a** and **N2a**) with the yields ranging from 36.4% to 93.5% (Fig. 3c, d and Additional file 1: Figs. S22, S103–S126), demonstrating that RrUGT3 is a promising UGT for preparation of *S*- and *N*-glucosides.

## Discussion

Alkylphenols are priority environmental pollutants, which are toxic, xenoestrogenic, and/or carcinogenic to humans and wildlife [46, 47]. Meanwhile, the derivatized alkylphenols are important synthetic precursors for a great variety of antioxidants, pesticides, and pharmaceuticals [48]. The cell-based cascade biotransformation established in this study effectively combines the processes of alkylphenol oxidation and UGT-mediated glucosylation. This strategy, in a "turn waste into value" way, exhibits great potentials in both biological degradation of various environmentally hazardous alkylphenolic compounds to generate useful precursors and bioproduction of more valuable glycoside drugs (or drug candidates).

UGT85A1 and RrUGT3 show high efficiency and complementary regioselectivity towards a select number of oxidized alkylphenols. The different characteristics may imply "function divergent evolution" of these two UGTs. The phylogenetic analysis (Additional file 1: Fig. S127) demonstrates that UGT85A1 and RrUGT3 are clustered into distinct clades, in agreement with their functional differences. Understanding of the molecular mechanisms for substrate promiscuity and regioselectivity will undoubtedly benefit the future rational engineering of UGTs to generate more glucoside compounds with new structures and activities. In the sequence alignment with homologous proteins, the catalytic dyad residues (H17/D118 of RrUGT3 and H24/D126 of UGT85A1) and plant secondary product glucosyltransferase (PSPG) motif (W345-Q388 of RrUGT3 and W363-Q406 of UGT85A1) are highly conserved (Additional file 1: Fig. S128). Furthermore, the AlphaFold2-simulated [49] structures of RrUGT3 and UGT85A1 are generally conserved (Additional file 1: Fig. S129), similar to other reported GT1 family members [50–52]. However, the architectures of the sugar acceptor and donor binding pockets of the two UGTs are dissimilar (Additional file 1: Fig. S130). In RrUGT3, the sugar acceptor **1** is enclosed in an entirely buried binding pocket formed by the hydrophobic residues L119, F120, F184, P186 and A386, and hydrophilic residues H17, D118, E85 and Y314 (Additional file 1: Fig. S130A). In UGT85A1, the aglycon **1** is anchored in the catalytic pocket by the surrounding amino acids H24, D126, W147, T149, and Y157 (Additional file 1: Fig. S130B). Thus, it could be speculated that the different topologies of sugar acceptor binding pockets and the specific interactions between substrate and active site residues should be responsible for complementary regioselectivity of RrUGT3 and UGT85A1. However, the mechanistic details remain unclear until the structural information is available. Thus, we are seeking to solve the crystal structures of these two UGTs and conduct structure-directed mutagenesis analysis, to elucidate the molecular mechanisms in a due course.

A considerable number of phenolic glucosides isolated from medicinal herbs have demonstrated diverse bioactivities, among which PEP inhibitory and anti-inflammatory activities are of particular importance. PEP is associated with some neurological disorders, such as Alzheimer’s disease and Parkinson’s Syndrome [53–55], thus having become an attractive drug target [56]. Inflammation is a complex biological response to various processes such as infection, tissue injury, cell death, cancer, ischemia, and degeneration [57, 58]. The development of potent PEP inhibitors and anti-inflammatory agents have been attracting growing attentions from both academia and pharmaceutical industries in recent years. Notably, many of the glycosidic products biosynthesized in this study demonstrated better PEP inhibitory and anti-inflammatory activities than the typical OTC drugs salidroside, gastrodin, and helicid. This strongly suggests the significant medicinal potential of these glucosides in the treatment of neurological and inflammatory diseases.

Many glucosides containing a carboxylic ester bond are the active ingredients of plant herbs. For instance, amarogentin, isolated from *Gentiana rigescens* Franch, is a good lead compound to develop new drugs for treating neurodegenerative diseases [59]. Forsythoside, originated from *Marrubium alysson*, acts as an inhibitor of several inflammatory mediators [60]. Some natural *S*- and *N*-glycosides also exhibit significant pharmacological activities, such as the anti-carcinogenic glucoraphanin [61, 62], the antioxidant glucoerucin [63], and the anticancer queuosine [64, 65]. The successful biosynthesis of carboxylic, *N*-, and *S*-glucosides in this study demonstrates the abilities of the plant-derived UGTs for promiscuous substrate recognition. It is anticipated that when the library of alkylphenolic aglycon substrates is expanded, more diverse glucosidic/glycosidic products could be produced by adopting the strategy developed in this study, which would hold great potential for screening of more biological activities.

## Conclusions

In summary, we characterized two highly efficient UGTs, UGT85A1 and RrUGT3, with distinct regioselectivity. A group of *O*-glucosides were efficiently synthesized by cell-based biotransformation using the two UGTs. Some of these unnatural glucosides exhibited promising PEP inhibitory or anti-inflammatory activities. The cascade biosynthetic systems of *O*-glucosides starting from alkylphenols and the additional versatile capacity to transfer glucosyl sugar towards *N*- or *S*-group further release the potential for glucoside-based drug development through biocatalytic approaches.

## Methods

### Strains, plasmids, and culture conditions

Strains and plasmids used in this study are listed in Additional file 1: Table S2. *E. coli* DH5α was used as a host for gene cloning and plasmid construction using Luria–Bertani (LB) agar plates or LB liquid media. *E. coli* BL21(DE3) was used for protein expression, purification and biotransformation and cultured in LB or Terrific Broth (TB: 1.2% tryptone, 2.4% yeast extract, 0.94% K_2_HPO_3_, 0.22% KH_2_PO_3_, 4% glycerol) media. All *E. coli* strains were grown at 37 °C unless otherwise specified. When required, appropriate antibiotics were added to the broth.

### Molecular manipulation, biochemical reagents, and chemicals

Gene cloning, plasmid transformation, agarose gel electrophoresis, and other standard techniques of molecular cloning were performed according to general protocols [66].

Primers were synthesized by Sangon Biotech (Shanghai, China) and are listed in Additional file 1: Table S1. Genes were synthesized by BGI Genomics (Shenzhen, China). E.Z.N.A.™ Plasmid Miniprep Kit (Omega Biotek, Norcross, GA) was used for plasmids isolation. E.Z.N.A.™ Gel Extraction Kit (Omega Biotek, Norcross, GA) was used for DNA fragment purification. TSINGKE TSE030 T3 Super PCR Mix (Tsingke Biotechnology Co., Ltd., Beijing, China) was used for colony PCR. PrimeSTAR (Takara Bio) or Phanta Max Super-Fidelity DNA Polymerase (Vazyme Biotech, Nanjing, China) were used for other routine PCR amplification. ClonExpress Ultra One Step Cloning Kit (Vazyme Biotech, Nanjing, China) and Hieff Clone® Plus One Step Cloning Kit (Cat No. 10911ES20; Yeasen, Shanghai, China) was used for plasmid construction. Macroporous resins AB-8 (Solarbio Science & Technology, Beijing, China) and S-8 (Solarbio Science & Technology, Beijing, China) were employed for products purification. All substrates and authentic standard compounds were purchased from Shanghai Aladdin Biochemical Technology Co., Ltd, Shanghai Macklin Biochemical Co., Ltd, or Sigma-Aldrich.

### Protein expression, purification, and concentration determination

A single colony of *E. coli* BL21(DE3) harboring certain expression plasmid(s) was grown in 5 mL LB broth containing 50 μg/mL kanamycin and incubated at 37 °C, 220 rpm overnight. The overnight culture (5 mL) was inoculated to 500 mL fresh TB broth containing kanamycin and incubated at 37 ℃, 220 rpm. When the cell density reached OD_600_ of 0.6–0.8, IPTG was added to a final concentration of 0.2 mM and the cells continued to grow at 18 °C for 24 h. The cells were then harvested by centrifugation at 6000×*g*, 4 ℃. The cell pellets were stored at −80 ℃ for later use.

All procedures of protein purification were performed at 4 ℃. Briefly, 50 mL of lysis buffer (50 mM NaH_2_PO_4_, 300 mM NaCl, 10% glycerol, and 10 mM imidazole, pH 8.0) was used to resuspend the cell pellets by vortexing. After sonication (3 s on, 5 s off, 30 min in total), the crude cell lysate was centrifuged at 12,000×*g* for 30 min. The supernatant fraction was collected, to which 1 mL of Ni–NTA resin (Qiagen, Germany) was added, and the mixture was subsequently incubated at 4 ℃ on a gentle rotator for 30 min. The slurry was then loaded onto an empty column for protein purification. The resin was washed with 100 mL of wash buffer (50 mM NaH_2_PO_4_, 300 mM NaCl, 10% glycerol, and 20 mM imidazole, pH 8.0) until no proteins were detectable in the flow-through by Coomassie Brilliant Blue G250 assay. Target proteins bound to the Ni–NTA resin were eluted by 10 ml of elution buffer (50 mM NaH_2_PO_4_, 300 mM NaCl, 10% glycerol, and 250 mM imidazole, pH 8.0). The eluent was concentrated with Amicon Ultra centrifugal filter (30 kD, Merck KGaA, Darmstadt, Germany), 5000×*g* for 30–60 min. Next, the protein solution was loaded onto a pre-equilibrated PD-10 column (GE Healthcare, Buckinghamshire, UK) for buffer exchange with 5 mL desalting buffer (50 mM NaH_2_PO_4_, 10% glycerol, pH 7.5). Finally, the desalted protein fraction was aliquoted, flash-frozen by liquid nitrogen, and stored at -80 ℃. The concentrations of proteins were determined by Bradford method [67] using BSA as standard. 15% Precast-Glgel Tris–Glycine PAGE gel (Sangon Biotech, Shanghai, China) and X-stain SDS-PAGE fast staining reagent (AtaGenix Laboratories Co., Ltd., Wuhan, China) were used for SDS-PAGE assays.

### Enzymatic assays

For UGT screening, the in vitro enzymatic reaction system contained 0.5 mM substrate, 10 mM MgCl_2_, 1 mM UDPG, and 10 μM purified enzyme. All enzymatic assays were carried out in 100 μL of 50 mM Tris·HCl buffer (pH 7.5) at 30 ℃ for 2 h. Reactions were quenched with two volumes of methanol.

### Genome editing

The CRISPR/Cas9 technology was adopted for *E. coli* genome editing, which was carried out based on the reports of Li et al. [68] and Wang et al. [35] with some modifications. The *ushA* gene was chosen as a locus for heterogeneous gene integration. pPaper was firstly modified to delete the 20 bp target sequence, yielding the vector pPaper2. Then *RrUGT3* integrating plasmid pPaper2-*gRNA-ushA-RrUGT3* containing the upstream and downstream homologous arms (1 kb, respectively) of *ushA* was constructed. Similarly, plasmid pPaper2-*gRNA-ushA-CreH-CreI-CreJ-SelFdR0978-SelFdx1499-CreD-RrUGT3* for simultaneous integration of RrUGT3 and the genes (*CreH*, *CreI*, *CreJ*, *SelFdR0978*, *SelFdx1499*, and *CreD*) involved in alkylphenol bio-oxidation system, were constructed by multi-fragments recombination.

*E. coli* BL21(DE3) was first transformed with plasmid pCas. A single colony was picked and cultured. When the cell concentration reached OD_600_ of 0.3, l-arabinose was added into the broth to a final concentration of 10 mM to induce *λ* Red recombinase, and Cas9. The electrocompetent cells were prepared after another 2 h cultivation (OD_600_ of 0.6–0.8). Plasmids pPaper2-*gRNA-ushA-RrUGT3* and pPaper2-*gRNA-ushA-CreH-CreI-CreJ-SelFdR0978-SelFdx1499-CreD-RrUGT3* were then respectively transformed into the electrocompetent cells by electroporation with MicroPulser Electroporator (Bio-Rad) using the Ec1 pre-programmed setting. The transformed cells were spread on LB plates after incubation at 30 ℃ for 2 h and grew at 30 ℃ for 36 h. Colony PCR was performed to obtain correct single clones with successful integration. After the genome editing was completed, the plasmids were cured by 3–5 generations of continuous culturing and resistance verification.

### Biotransformation

Biotransformation of phenolic substrates was carried out in shaking flasks. *E. coli* BL21(DE3) strains were inoculated into LB broth with appropriate selective antibiotics and cultivated at 37 ℃ overnight. The seed culture was inoculated (1:100) into fermentation broth M9CA (4% glucose, casamino acid 0.2%, Na_2_HPO_4_ 0.68%, KH_2_PO_4_ 0.3%, NH_4_Cl 0.1%, NaCl 0.05%, MgSO_4_ 0.24%, CaCl_2_ 0.01%) and followed by cultivation at 37 ℃, 220 rpm on a rotary shaker. When OD_600_ reached 0.8, protein expression was induced by adding IPTG to a final concentration of 1 mM. Substrate (dissolved in DMSO) was simultaneously fed into the culture to a final concentration of 1 mM. The culture was then incubated at 30 ℃. Biotransformation with cell growth continued for an additional 48 h. After biotransformation, the supernatant of broth was collected by centrifugation at 5000×*g* for 5 min.

### Isolation and purification of glucosides

Macroporous adsorption resin was firstly used to prepare crude glucoside products. Before use, the macroporous resin was soaked into 95% ethanol for 24 h to desorb unnecessary impurities. Then the resin was poured into a glass column, followed by washing with 95% ethanol of 2 column volumes. Then distilled water (5 column volumes) was used to remove ethanol. The collected supernatant containing glucosides was loaded into the resin column with a flow rate of 1 BV/h. The maximum loading volume was fivefold the volume of the column. After absorption, 5 column volume of distilled water was used to wash the resin. Crude glucosides were then eluted with 40% ethanol. The eluent was concentrated by a rotary evaporator and dissolved in 5–20 mL methanol. Further purification was carried out through preparative HPLC. Final products were lyophilized into powder and stored at -80 ℃ for long-term preservation.

### Structure identification

Products gastrodin and helicid were confirmed by comparing the consistency of their retention time on HPLC with that of corresponding authentic commercial standards. Other glucosides were not commercially available and their structures were confirmed by HRMS recorded in the positive (or negative ionization) mode on an Impact HD QTOF Mass Spectrometer (Bruker) and NMR (AVANCE NEO, 600 MHz, Bruker) analysis.

### Analytical methods

Samples were analyzed on an Agilent 1260 or Thermo Infinity HPLC system with a photodiode array detector.

For UGT screening assays, enzymatic samples were separated on a YMC Triart-C18 column by using a linear mobile phase gradient ranging from 5% acetonitrile in 0.1% TFA aqueous solution to 70% acetonitrile in 0.1% TFA aqueous solution over 25 min. For assays of biotransformation and mutant activity determination, supernatant of fermentation broth or enzymatic samples were separated using a linear mobile phase gradient ranging from 10% acetonitrile in 0.1% TFA aqueous solution to 100% acetonitrile in 0.1% TFA aqueous solution over 25 min. The flow rate was set to 1 mL/min and the injection volume was 5–20 μL. Full-wavelength scans (210–400 nm) were recorded.

For purification of glucosides, preparative HPLC were used. Suitable conditions according to the polarity of each glucosides were explored. Purification was performed on a YMC Triart-C18 (20 × 250 mm) using isocratic elution method with fixed mobile phase (10–30% acetonitrile aqueous solution for different glucosides) at a flow rate of 19 mL/min. The injection volume was 200–1000 μL. Wavelength was set at 210 and 230 nm.

Substrate consumption and product formation were quantified by HPLC peak area integration using corresponding authentic compounds as standards (commercialized or prepared by this study).

### Prolyl endopeptidase (PEP) inhibitory assay

The inhibitory activity of glucosides to PEP was determined by a modified Yoshimoto T’s method [69]. Firstly, the activity of the purified PEP enzyme was measured using Z-Gly-Pro-4-nitroanilide (GPNA) as a substrate. The reaction mixture contained 1 mM GPNA, 1/10 (v/v) PEP solution (pre-diluted to an appropriate concentration) and 100 mM Tris–HCl buffer (pH 7.0). The reaction was incubated for 10 min at 37℃ and stopped by the addition of 3 volumes of 0.2 M Na_2_CO_3_ buffer. The reaction without the addition of PEP enzyme was carried out as the control. The formation of product 4-nitroanilide was measured at 410 nm. The activity of the PEP enzyme was calculated as follow:

1 $$Activity\,{\mkern 1mu} of{\mkern 1mu} PEP{\mkern 1mu} enzyme\,\,\left( {U/mL} \right){\mkern 1mu} = {\mkern 1mu} \Delta A{\mkern 1mu} \times {\mkern 1mu} n{\mkern 1mu} \times {\mkern 1mu} 10^{6} /\gamma$$

where n represents total dilution factor, *γ* represents the extinction coefficient constant of 8800 M^−1^·cm^−1^. For determination of PEP inhibitory activity of glucosides, the sample mixture contained 0.005 U/mL PEP, 0.2 mM GPNA, 500 μM glucoside, and 100 mM Tris–HCl buffer (pH 7.0). The control reaction was carried out without the addition of glucoside. All reaction mixtures were incubated at 30 ℃ and the absorption was measured at 410 nm wavelength with an interval of 1 min for a total of 15 min. Percent PEP inhibitory activity was calculated as follow:

2 $$Inhibition \, rate\, = \,\left( {{1} - Slop_{sample} /Slop_{control} } \right)\, \times \,{1}00\%$$

where Slop represents absorbance *versus* time. The IC_50_ values of the glucosides with inhibition rates greater than 50% were further determined with inhibitor concentrations varying from 10 to 1000 μM.

### Cytotoxic activity test

In the CCK8 assay, the cells were cultured in DMEM (High Glucose, Gibco, USA) containing 10% FBS (Gibco, USA) and 1% penicillin–streptomycin solution (Gibco, USA) under a humidified atmosphere of 5% CO_2_ at 37 ℃. The cells were treated with 0.25% Trypsin (Hyclone, USA) and then plated in 96-well plate at a density of 10,000 cells/well to incubate for 24 h at the above conditions. After that, the cells were treated with the synthesized compounds at a concentration of 10 μM for another 24 h (using 5 μg/mL adriamycin as positive control and blank DMSO as negative control). After that, the medium was gently replaced with 100 μL 10% CCK8 (MCE, USA) solution and further incubated for 1 h. The optical density of the final solution was measured on a Spectra Max Plus plate reader at a wavelength of 450 nm. All experiments were repeated three times in three wells of the microplate. The compound with a cell survival-rate below 60% was considered as cytotoxic.

### Anti-inflammatory assay

The RAW264.7 cells were cultured in the DMEM medium (Gibco, USA) supplemented with 10% fetal bovine serum (Hyclone, USA), 100 U/mL penicillin and 100 µg/mL streptomycin (Gibco, USA) at 37 ℃ in a humidified atmosphere containing 5% CO_2_. The cells were passaged every two days. The RAW264.7 cells were collected by the digestion of 0.25% trypsin (Gibco, USA), then were seeded into a 96-well plate with 2 × 10^4^ cells per well and incubated for 24 h in 200 µL media. The media were then discarded, and the cells were co-incubated with 1 µg/ml LPS (Sigma, USA) and a given concentration of the tested compounds or indomethacin (Sigma, USA) as a control in 100 µL newly added medium for 24 h. After that, the supernatants were collected and examined for NO production using Griess reagent (Beyotime, China). Four parallel assays were performed to eliminate the experimental errors.

## Supplementary Information

**Additional file 1.** Supplementary tables and figures

## Data Availability

All data generated or analyzed during this study are included in this published article [and its supplementary information files].

## Abbreviations

GPNA: Z-Gly-Pro-4-nitroanilide
HPLC: High performance liquid chromatography
HRMS: High resolution mass spectrometry
IPTG: Isopropyl *β*-D-thiogalactoside
LB: Luria–Bertani
NMR: Nuclear magnetic resonance
OTC: Over-the-counter
PCR: Polymerase chain reaction
PEP: Prolyl endopeptidase
PSPG: Plant secondary product glucosyltransferase
TB: Terrific broth
TFA: Trifluoroacetic acid
UDP: Uridine diphosphate
UDPG: UDP-glucose
UGT: UDP-glucosyltransferase
