# Supplementary material for: Diversification of phenolic glucosides by two UDP-glucosyltransferases featuring complementary regioselectivity
Source: Microb Cell Fact. 2022 Oct 10;21:208. doi: 10.1186/s12934-022-01935-w (PMC9549646; doi:10.1186/s12934-022-01935-w)
Supplement: Supplementary file 1 — Additional file 1. Supplementary tables and figures [file 12934_2022_1935_MOESM1_ESM.pdf]

Supplementary Information

Diversification of phenolic glucosides by two UDP-glucosyltransferases featuring complementary regioselectivity

Fei Guo[1, †], Xingwang Zhang[1, †], Cai You[1,3], Chengjie Zhang[1], Fengwei Li[1], Nan Li[6], Yuwei Xia[5], Mingyu Liu[1], Zetian Qiu[4], Xianliang Zheng[10], Li Ma[1], Gang Zhang[7], Lianzhong Luo[7], Fei Cao[8], Yingang Feng[3], Guang-Rong Zhao[4], Wei Zhang[1], Shengying Li[1,9], Lei Du[1,2*]

[1]State Key Laboratory of Microbial Technology, Shandong University, Qingdao, Shandong, 266237, China

[2]Shenzhen Branch, Guangdong Laboratory for Lingnan Modern Agriculture, Genome Analysis Laboratory of the Ministry of Agriculture, Agricultural Genomics Institute at Shenzhen, Chinese Academy of Agricultural Sciences, Shenzhen, 518000, China

[3]Shandong Provincial Key Laboratory of Synthetic Biology, CAS Key Laboratory of Biofuels, Qingdao Institute of Bioenergy and Bioprocess Technology, Chinese Academy of Sciences, Qingdao, Shandong, 266101, China

[4]Frontier Science Center for Synthetic Biology and Key Laboratory of Systems Bioengineering (Ministry of Education), School of Chemical Engineering and Technology, Tianjin University, Yaguan Road 135, Jinnan District, Tianjin, 300350, China

[5]Key Laboratory of Marine Drugs, Ministry of Education of China, School of Medicine and Pharmacy, Ocean University of China, Qingdao, Shandong, 266003, China

[6]Tianjin University of Science and Technology, College of Biotechnology, Tianjin, 300457, China

[7]Fujian Universities and Colleges Engineering Research Center of Marine Biopharmaceutical Resources, Xiamen Medical College, Xiamen, Fujian, 361023, China

[8]College of Pharmaceutical Sciences, Hebei University, Baoding, 071002, China

[9]Laboratory for Marine Biology and Biotechnology, Qingdao National Laboratory for Marine Science and Technology, Qingdao, Shandong, 266237, China

[10]Center For Biocatalysis and Enzyme Technology, Angel Yeast Co., LTD, Cheng Dong Avenue, Yichang, Hubei, 443003, China

[†]These authors contributed equally.

*Corresponding author: Lei Du. lei.du@sdu.edu.cn.

Table of Contents

Supplementary Tables..4

Supplementary Figures 11

References...81

Supplementary Tables

Table S1 Primers used in this study.

Primer	Sequence（5’-3’）	Description
P1	CTTTAATAAGGAGATATACCATG TCTGGCACCCCACA	For amplification of *RrUGT3* in construction of plasmid pCDFDuet-1-*RrUGT3*
P2	ATGATGGTGATGGCTGCTGCTTA ATGCTTCATAGAAGAAC	
P3	CTTTAATAAGGAGATATACCATG GGTTCTCAGATCATTCA	For amplification of *UGT85A1* in construction of plasmid pCDFDuet-1-*UGT85A1*
P4	ATGATGGTGATGGCTGCTGCTTA ATCCTGAGATTTTTGAC	
P5	TGGTGCCGCGCGGCAGCCATATG GGTTCTCAGATCATTCA	For amplification of *UGT85A1* in construction of plasmid pET28b (+)-*UGT85A1*
P6	TGTCCACCAGTCATGCTAGCTTAA TCCTGAGATTTTTGAC	
P7	TGGTGCCGCGCGGCAGCCATATG TCTGGCACCCCACACAT	For amplification of *RrUGT3* in construction of plasmid pET28b (+)-*RrUGT3*
P8	TGTCCACCAGTCATGCTAGCTTAA TGCTTCATAGAAGAAC	
P9	TGGTGCCGCGCGGCAGCCATATG GGCTCTCTGCCGTCCAC	For amplification of *RrUGT17* in construction of plasmid pET28b (+)-*RrUGT17*
P10	TGTCCACCAGTCATGCTAGCTTAA ACGCTAAACTGAACCA	
P11	TGGTGCCGCGCGGCAGCCATATG AGCCTGATTGAAAAACC	For amplification of *RrUGT33* in construction of plasmid pET28b (+)-*RrUGT33*
P12	TGTCCACCAGTCATGCTAGCTTAA CGGATATGTTTGGTTT	
P13	TGGTGCCGCGCGGCAGCCATATG GCTGGTTCTGGTACTGG	For amplification of *UGT72B14* in construction of plasmid pET28b (+)-*UGT72B14*
P14	TGTCCACCAGTCATGCTAGCTTAG TGTTTAACAGAAGAAC	
P15	TGGTGCCGCGCGGCAGCCATATG GGTTCTGAAACCCGTCC	For amplification of *UGT73B6* in construction of plasmid pET28b (+)-*UGT73B6*
P16	TGTCCACCAGTCATGCTAGCTTAA ACTTTTTTCAGTTTCA	
P17	TGGTGCCGCGCGGCAGCCATATG GGTTCTGAAACCCGTCC	For amplification of *UGT73B6FS* in construction of plasmid pET28b (+)-*UGT73B6FS*
P18	TGTCCACCAGTCATGCTAGCTTAA ACTTTTTTCAGTTTCA	
P19	TGGTGCCGCGCGGCAGCCATATG GGTCATAAACATATCGC	For amplification of *UGTYjiC* in construction of plasmid pET28b (+)-*UGTYjiC*
P20	TGTCCACCAGTCATGCTAGCTTAT TTTACACCTGCCGGTG	

Table S1 Primers used in this study (continued).

Primer	Sequence（5'-3'）	Description
P21	CTTTAATAAGGAGATATACCATGGGCAGCAGCCATCACCA	For amplification of *CreH-CreI-CreJ* and *CreE-CreF-CreD* in construction of plasmid pRSFDuet-1-*CreH-CreI-CreJ-CreE-CreF-CreD*
P22	TATGCGGCCGCAAGCTTGTCCTAGGCATGTGTATCCACCC	
P23	TATAAGAAGGAGATATACATATGTCTACTATTCATTTCAT	
P24	TTACCAGACTCGAGGGTACCTCATCGGATCGCATCCGTTC	
P25	TATAAGAAGGAGATATACATATGGCAACCTACAAGGTTAC	For amplification of *SelFdx1499*, *SelFdR0978* and *CreD* in construction of plasmid pRSFDuet-1-*CreH-CreI-CreJ-SelFdR0978-SelFdx1499-CreD*
P26	CACACTTGCGTTTCTGGCGTGTAGAGGTCTTCTTCTTTGT	
P27	ACGCCAGAAACGCAAGTGTGAGGTTGAATCATGTTGAATGCGAGTGTGGC	
P28	TCCAGGTCAACGGCTAGTTTGTAGGTTTCAACATGCCAAC	
P29	AAACTAGCCGTTGACCTGGAGGTTACCCGATGACTCGCAGTAATTTACC	
P30	TTACCAGACTCGAGGGTACCTCATCGGATCGCATCCGTTC	
P31	ATATTCCAGCGAGATCTTGGTGCAAAAC	For amplification of pPaper backbone in construction of plasmid pPaper2
P32	ATCTCGCTGGAATATTTTATCTGATTAATAAGATGAT	
P33	AGGTATAATACTAGTTATTATCGCGGCGACCCATAGTTTTAGAGCTAGAAATAGCAAGT	For amplification of gRNA of *ushA*, *up* arm of *ushA*, down arm of *ushA* and pPaper2 backbone in construction of plasmid pPaper2-*gRNA-ushA*
P34	CAGGACGTATCGGTATGGTTGGTCGACTCTAGAGAATTCA	
P35	AACCATACCGATACGTCCTG	
P36	TTTCGGAGGATCCACTTCTCTCCCTGACCTGAT	
P37	GAGAAGTGGATCCTCCGAAAGTGCCGGATGTTTG	
P38	GGTAATAGATCTAAGCTTCACCCGTGATTGAACTGGAAGG	
P39	GAAGCTTAGATCTATTACCCTG	
P40	ACTAGTATTATACCTAGGACTGAG	

Table S1 Primers used in this study (continued).

P41	ATCAGGTCAGGGAGAGAAGTGAAATTAATACGACTCACTATAGG	For amplification of *RrUGT3* in construction of plasmid pPaper2-*gRNA-ushA-RrUGT3*
P42	AAACATCCGGCACTTTCGGAGGATCCTTAATGCTTCATAGAAGAACGCCA	
P43	GTTCTTCTATGAAGCATTAACTTATGCGACTCCTGCATTA	For amplification of *CreH-CreI-CreJ-SelFdR0978-SelFdx1499-CreD* in construction of plasmid pPaper2-*gRNA-ushA-CreH-CreI-CreJ-SelFdR0978-SelFdx1499-CreD-RrUGT3*
P44	AACATCCGGCACTTTCGGAGGATCCTCATCGGATCGCATCCGTTC	
P45	GCGCACTGATCCCGATGGCCGA	For amplification of *RrUGT3(H17A)* in construction of plasmid pET28b (+)-*RrUGT3(H17A)*
P46	GGATCAGTGCGCCCATGCCCGGGCT	
P47	GTTGTTGCACTGTTCGGCACTGATGCATTC	For amplification of *RrUGT3(D118A)* in construction of plasmid pET28b (+)-*RrUGT3(D118A)*
P48	GAACAGTGCAACAACCAGAGAAACCAGGTT	
P49	GATCTGGCAGGCACTGATGCATTCGACCCG	For amplification of *RrUGT3(F120A)* in construction of plasmid pET28b (+)-*RrUGT3(F120A)*
P50	AGTGCCTGCCAGATCAACAACCAGAGAAAC	
P51	ACCGCATTCTCTGTACAAAGCCA	For amplification of *RrUGT3(Y314A)* in construction of plasmid pET28b (+)-*RrUGT3(Y314A)*
P52	AGAGAATGCGGTGCTGTTCGGGATTTGGT	
P53	CGTATCGCAACCCTGATCTCCCTGACCGTT	For amplification of *RrUGT3(E85A)* in construction of plasmid pET28b (+)-*RrUGT3(E85A)*
P54	CAGGGTTGCGATACGCGGATGAGCTGGAGA	
P55	CAAGGCGCAATCAACCCGATGATGCGTGTG	For amplification of *UGT85A1(H24A)* in construction of plasmid pET28b (+)-*UGT85A1(H24A)*
P56	GTTGATTGCGCCTTGAGCCGGATATGGAAC	
P57	GTATCTGCAGGTTGTATGAGCTTTACTCTG	For amplification of *UGT85A1(D126A)* in construction of plasmid pET28b (+)-*UGT85A1(D126A)*
P58	ACAACCTGCAGATACAATACAGCTTACCGG	
P59	CTGTTCGCAACCACCAGCGGCTGTGCGTTC	For amplification of *UGT85A1(W147A)* in construction of plasmid pET28b (+)-*UGT85A1(W147A)*
P60	GGTGGTTGCGAACAGAACCTCGGGAACACC	
P61	CTGCACTGCACTTTTATCTGTTCAT	For amplification of *UGT85A1(Y157A)* in construction of plasmid pET28b (+)-*UGT85A1(Y157A)*
P62	AGTGCAGTGCAGCCAGGAACGCACA	

Table S2 Strains and plasmids used in this study.

Strain	Host	Genotype/Description	Source
**GF-1**	*E. coli* BL21(DE3)	pCDFDuet-1-*RrUGT3*. Heterologous expression of *RrUGT3*	This study
**GF-2**	*E. coli* BL21(DE3)	pCDFDuet-1-*UGT85A1*. Heterologous expression of *UGT85A1*	This study
**GF-3**	*E. coli* BL21(DE3)	Harboring two plasmids, pRSFDuet-1-*CreH-CreI-CreJ-SelFdR0978-SelFdx1499-CreD* and pCDFDuet-1-*RrUGT3*. Heterologous expression of optimized alkyl phenol oxidation system and *RrUGT3*	This study
**GF-4**	*E. coli* BL21(DE3)	Harboring two plasmids, pRSFDuet-1-*CreH-CreI-CreJ-SelFdR0978-SelFdx1499-CreD* and pCDFDuet-1-*UGT85A1*. Heterologous expression of optimized alkyl phenol oxidation system and *UGT85A1*	This study
**GF-5**	*E. coli* BL21(DE3)	Δ*ushA*::*RrUGT3*	This study
**GF-6**	*E. coli* BL21(DE3)	Δ*ushA*::*CreH-CreI-CreJ-SelFdR0978-SelFdx1499-CreD-RrUGT3*	This study
**GF-7**	*E. coli* DH5α	pET28b (+). Backbone vector for gene expression	Lab stock
**GF-8**	*E. coli* BL21(DE3)	pET28b (+)-*UGT85A1*. Heterologous expression of *UGT85A1*	This study
**GF-9**	*E. coli* BL21(DE3)	pET28b (+)-*RrUGT3*. Heterologous expression of *RrUGT3*	This study
**GF-10**	*E. coli* BL21(DE3)	pET28b (+)-*RrUGT17*. Heterologous expression of *RrUGT17*	This study
**GF-11**	*E. coli* BL21(DE3)	pET28b (+)-*RrUGT33*. Heterologous expression of *RrUGT33*	This study
**GF-12**	*E. coli* BL21(DE3)	pET28b (+)-*UGT72B14*. Heterologous expression of *UGT72B14*	This study
**GF-13**	*E. coli* BL21(DE3)	pET28b (+)-*UGT73B6*. Heterologous expression of *UGT73B6*	This study

Table S2 Strains and plasmids used in this study (continued).

Strain	Host	Genotype/Description	Source
**GF-14**	*E. coli* BL21(DE3)	pET28b (+)-*UGT73B6FS*. Heterologous expression of *UGT73B6FS*	This study
**GF-15**	*E. coli* BL21(DE3)	pET28b (+)-*UGTYjiC*. Heterologous expression of *UGTYjiC*	this study
**GF-16**	*E. coli* DH5α	pRSFDuet-1. Backbone vector for expression of alkyl phenol oxidation system	Lab stock
**GF-17**	*E. coli* BL21(DE3)	pRSFDuet-1-*CreH-CreI-CreJ-CreE-CreF-CreD*. Heterologous expression of the original alkylphenol oxidation system	This study
**GF-18**	*E. coli* BL21(DE3)	pRSFDuet-1-*CreH-CreI-CreJ-SelFdR0978-SelFdx1499-CreD*. Heterologous expression of the optimized alkylphenol oxidation system	This study
**GF-19**	*E. coli* DH5α	pCDFDuet-1. Backbone vector for expression of UGTs	Lab stock
**GF-20**	*E. coli* BL21(DE3)	pCas. For expression of λ Red and Cas9	Wang J., et al., 2021 [1]
**GF-21**	*E. coli* BL21(DE3)	pPaper. Backbone vector for gene knockout	Wang J., et al., 2021 [1]
**GF-22**	*E. coli* DH5α	pPaper2. A modify vector from pPaper, with 20bp sequence deleted	This study
**GF-23**	*E. coli* DH5α	pPaper2-*gRNA-ushA*. For *ushA* knockout	This study
**GF-24**	*E. coli* DH5α	pPaper2-*gRNA-ushA-RrUGT3*. For *ushA* knockout and *RrUGT3* integration	This study
**GF-25**	*E. coli* DH5α	pPaper2-*gRNA-ushA-CreH-CreI-CreJ-SelFdR0978-SelFdx1499-CreD-RrUGT3*. For *ushA* knockout and *CreH-CreI-CreJ-SelFdR0978-SelFdx1499-CreD-RrUGT3* integration	This study
**GF-26**	*E. coli* BL21(DE3)	pET28b (+)-*RrUGT3(H17A)*. For expression of *RrUGT3(H17A)* (a mutant of *RrUGT3*)	This study
**GF-27**	*E. coli* BL21(DE3)	pET28b (+)-*RrUGT3(D118A)*. For expression of *RrUGT3(D118A)* (a mutant of *RrUGT3*)	This study

Table S2 Strains and plasmids used in this study (continued).

Strain	Host	Genotype/Description	Source
**GF-28**	*E. coli* BL21(DE3)	pET28b (+)-*RrUGT3(F120A)*. For expression of *RrUGT3(F120A)* (a mutant of *RrUGT3*)	This study
**GF-29**	*E. coli* BL21(DE3)	pET28b (+)-*RrUGT3(Y314A)*. For expression of *RrUGT3(Y314A)* (a mutant of *RrUGT3*)	This study
**GF-30**	*E. coli* BL21(DE3)	pET28b (+)-*RrUGT3(E85A)*. For expression of *RrUGT3(E85A)* (a mutant of *RrUGT3*)	This study
**GF-31**	*E. coli* BL21(DE3)	pET28b (+)-*UGT85A1(H24A)*. For expression of *UGT85A1(H24A)* (a mutant of *UGT85A1*)	This study
**GF-32**	*E. coli* BL21(DE3)	pET28b (+)-*UGT85A1(D126A)*. For expression of *UGT85A1(D126A)* (a mutant of *UGT85A1*)	This study
**GF-33**	*E. coli* BL21(DE3)	pET28b (+)-*UGT85A1(W147A)*. For expression of *UGT85A1(W147A)* (a mutant of *UGT85A1*)	This study
**GF-34**	*E. coli* BL21(DE3)	pET28b (+)-*UGT85A1(Y157A)*. For expression of *UGT85A1(Y157A)* (a mutant of *UGT85A1*)	This study

Table S3 Adsorption yield of glycosides on macroporous resin (AB-8).

**Glycosides**	**Adsorption yield (%)**
**1a**	55.5
**1b**	43.8
**2a**	67.6
**3a**	53.6
**4a**	90.2
**5a**	81.2
**6a**	55.0
**7a**	78.0
**8a**	58.5
**10a**	79.0
**12b**	57.4
**13a**	85.7
**13b**	83.8
**14a**	70.6
**14b**	71.9
**15a**	56.5
**16a**	80.6
**17a**	69.4
**18a**	68.6
**19a**	92.2
**20a**	58.3
**20b**	91.1
**21b**	58.3
**S1a**	78.7
**S2a**	80.0
**S3a**	91.5

Supplementary Figures

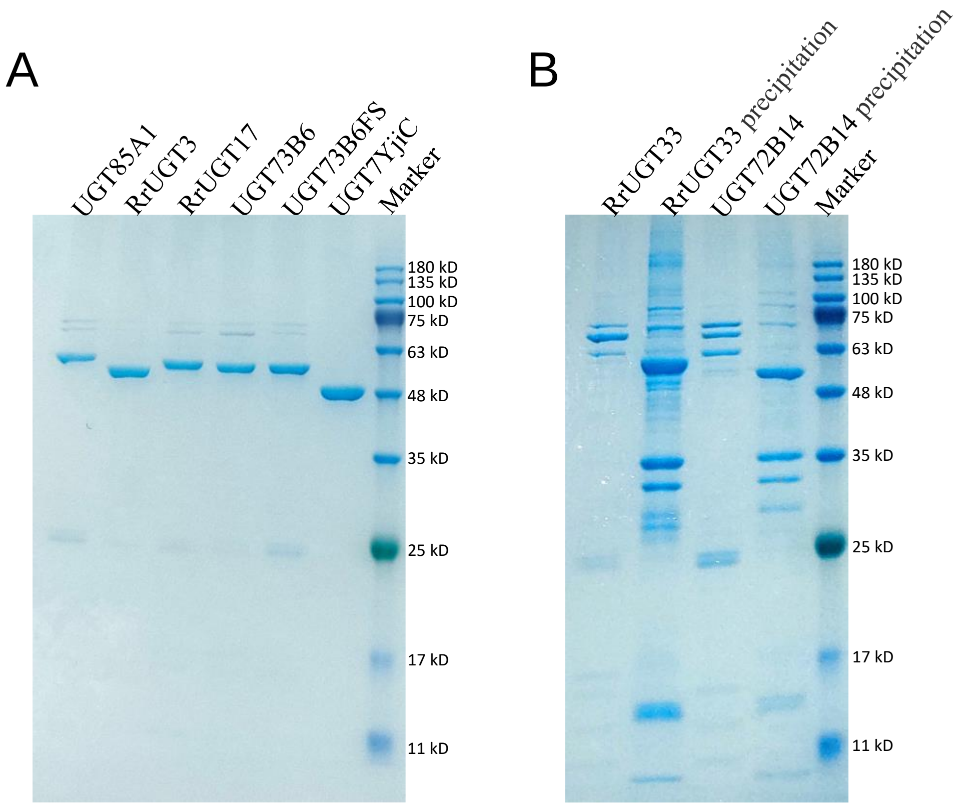

Fig. S1. SDS-PAGE analysis of purified 8 UGTs. (A) The six well expressed UGTs. UGT85A1, 55.0 kDa; RrUGT3, 51.9 kDa; RrUGT17, 54.4 kDa; RrUGT33, 55.7 kDa; UGT72B14, 51.6 kDa; UGT73B6, 53.9 kDa; UGT73B6FS, 53.9 kDa; UGTYjiC, 44.6 kDa. (B) The other two UGTs. RrUGT33, 55.7 kDa; UGT72B14, 51.6 kDa. RrUGT33 and UGT72B14 were mostly distributed in precipitation, with only low concentration of target protein in the purified solution.

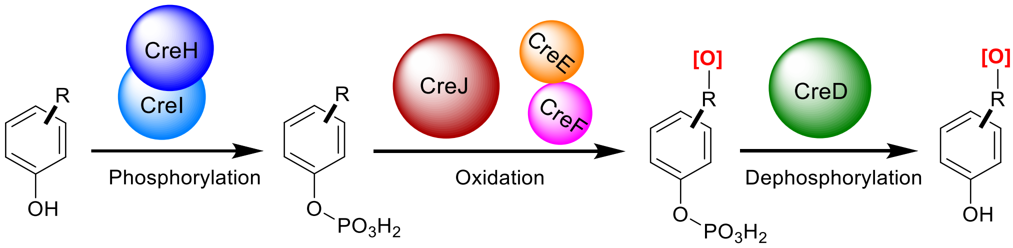

Fig. S2. Reaction scheme for alkylphenol oxidation. CreH, Phosphate synthase subunit; CreI, Phosphate synthase subunit; CreJ, Cytochrome P450; CreE, Ferredoxin reductase; CreF, Ferredoxin; CreD, Phosphohydrolase.

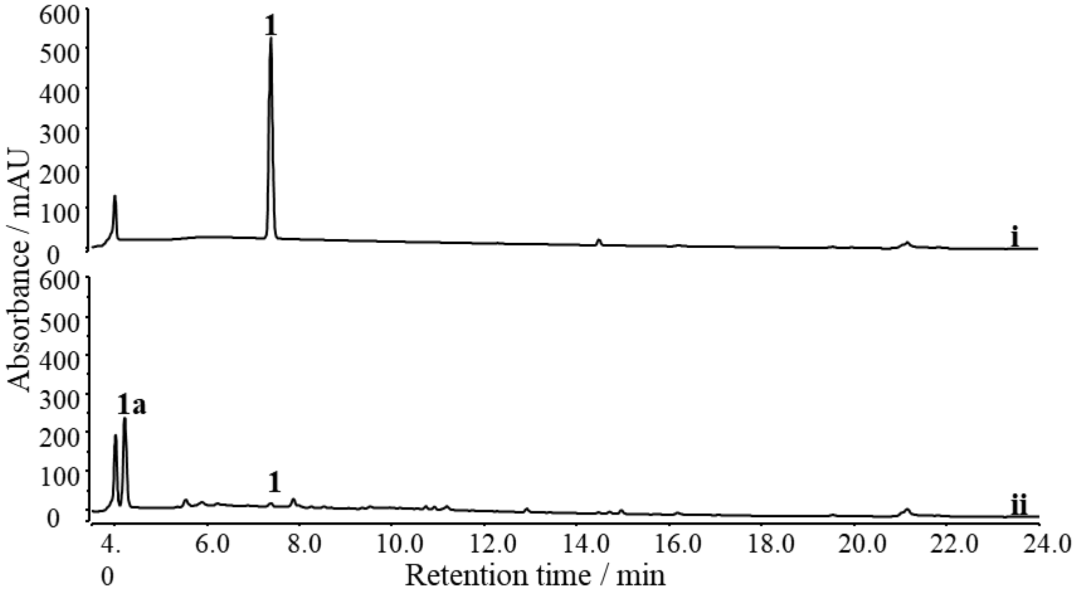

Fig. S3. Transformation of substrate 1 by *E. coli* BL21(DE3) containing RrUGT3.
i, standard substrate **1**; ii, biotransformation. The conversion efficiency is 99.02%.

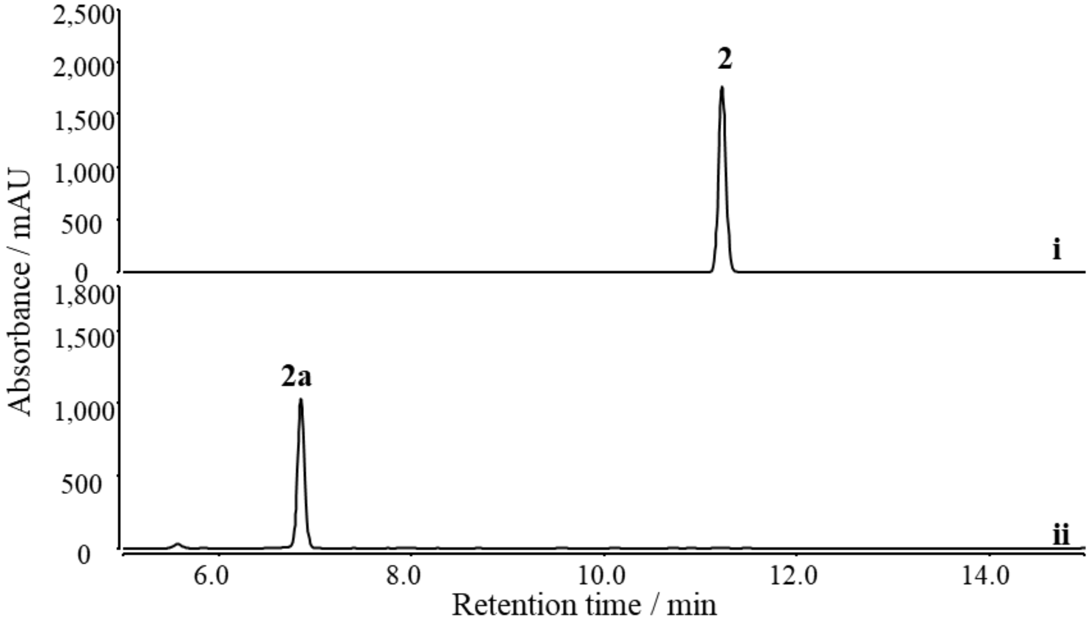

Fig. S4. Transformation of substrate 2 by *E. coli* BL21(DE3) containing RrUGT3.
i, standard substrate **2**; ii, biotransformation. The conversion efficiency is 100%.

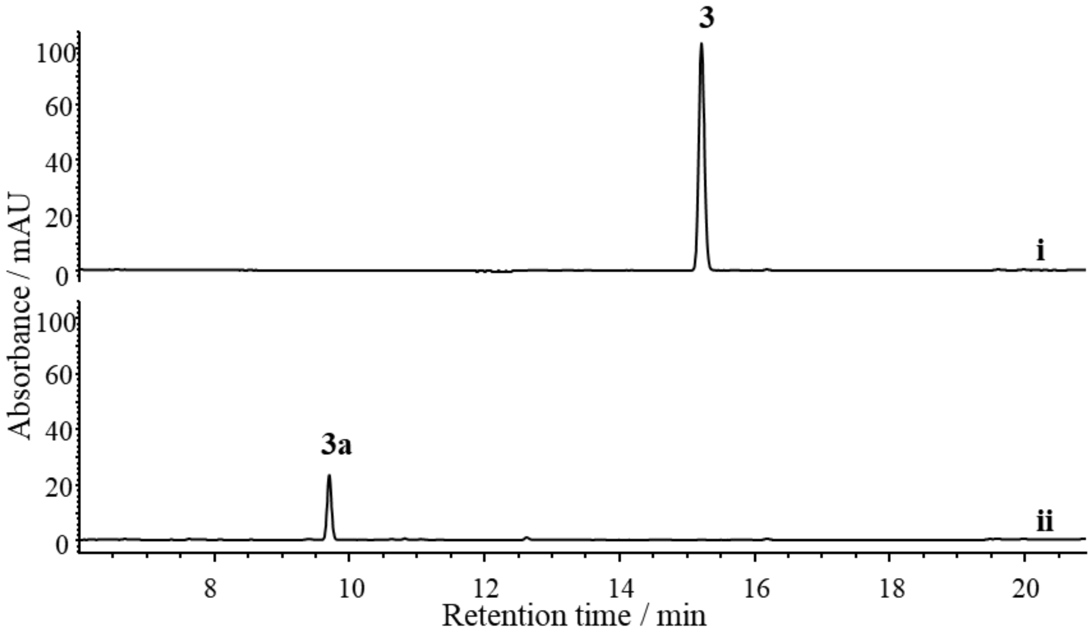

Fig. S5. Transformation of substrate 3 by *E. coli* BL21(DE3) containing RrUGT3. i, standard substrate **3**; ii, biotransformation. The conversion efficiency is 100%.

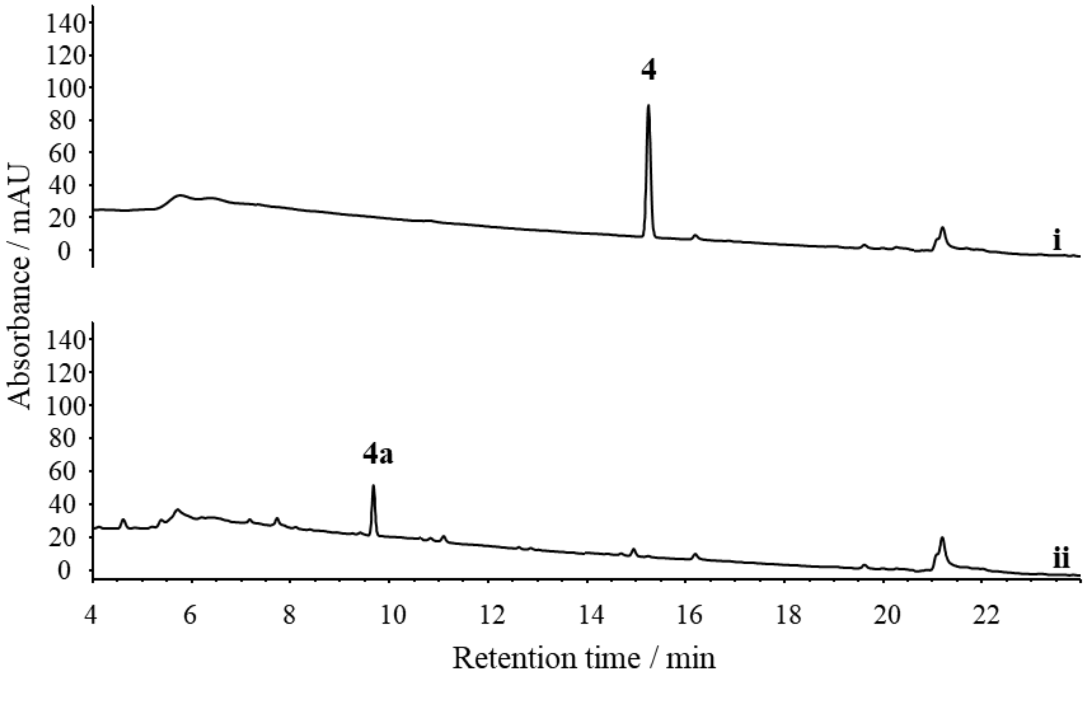

Fig. S6. Transformation of substrate 4 by *E. coli* BL21(DE3) containing RrUGT3. i, standard substrate **4**; ii, biotransformation. The conversion efficiency is 100%.

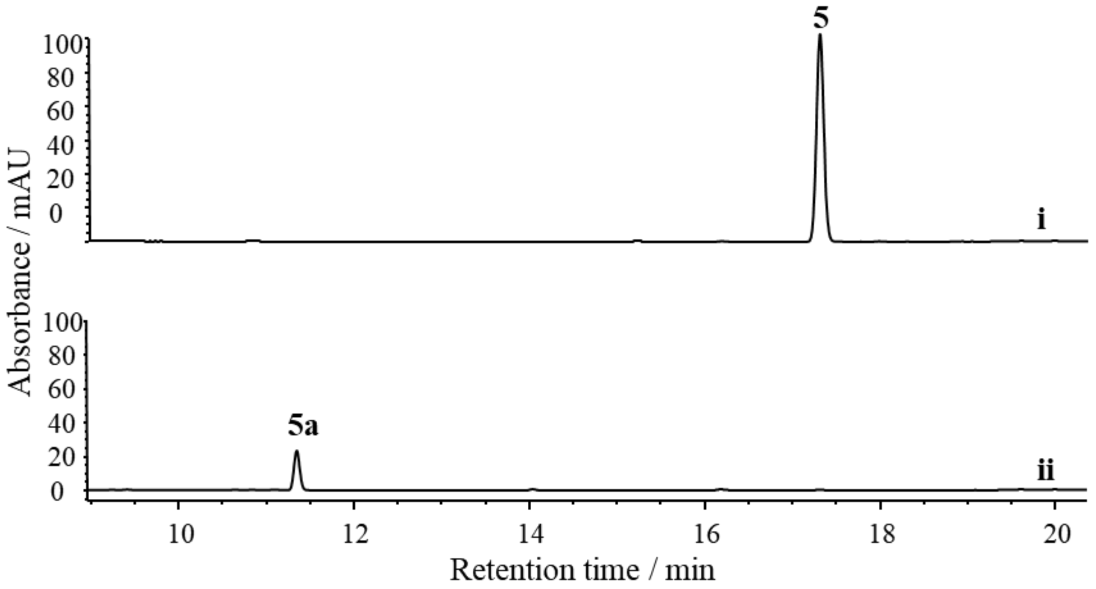

Fig. S7. Transformation of substrate 5 by *E. coli* BL21(DE3) containing RrUGT3. i, standard substrate **5**; ii, biotransformation. The conversion efficiency is 100%.

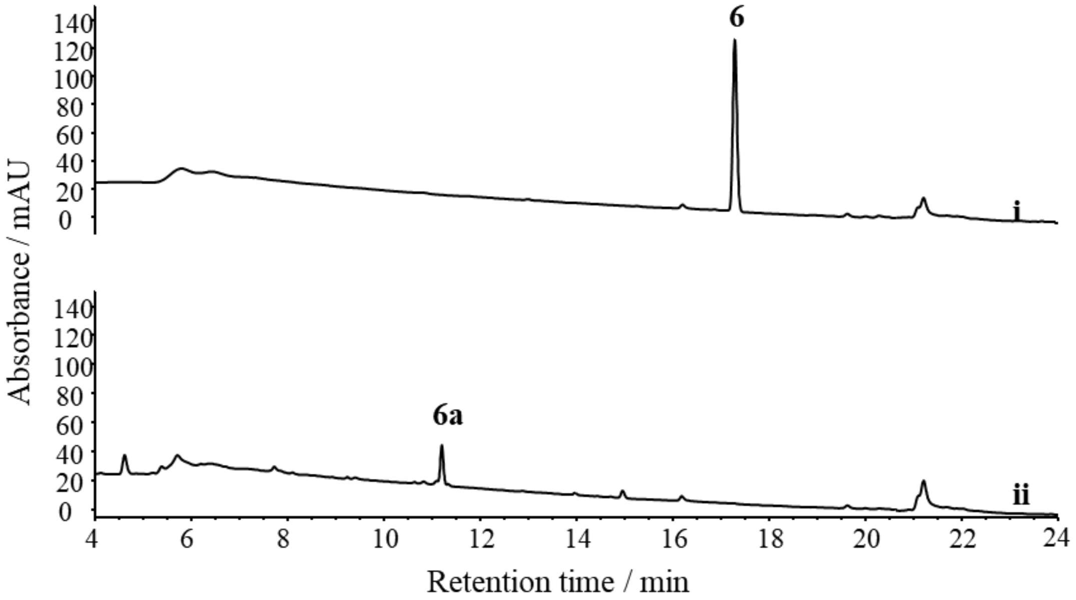

Fig. S8. Transformation of substrate 6 by *E. coli* BL21(DE3) containing RrUGT3. i, standard substrate **6**; ii, biotransformation. The conversion efficiency is 100%.

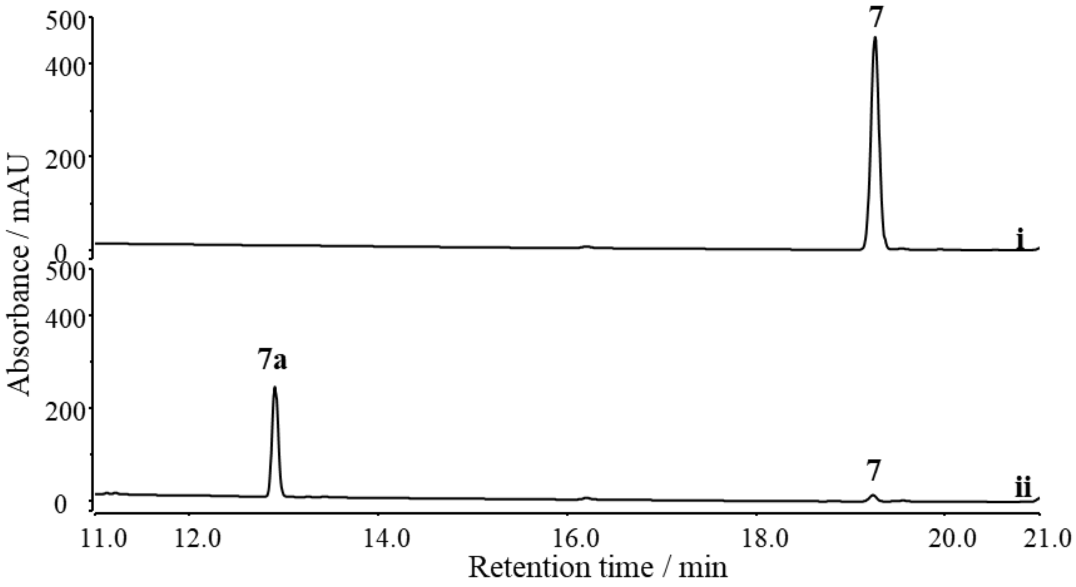

Fig. S9. Transformation of substrate 7 by *E. coli* BL21(DE3) containing RrUGT3. i, standard substrate **7**; ii, biotransformation. The conversion efficiency is 88.55%.

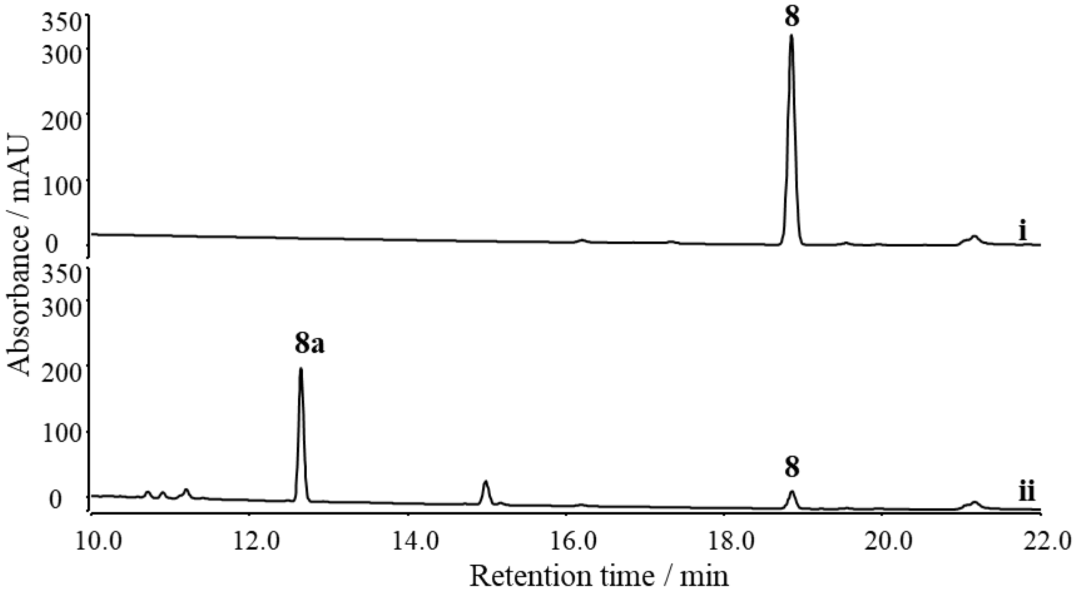

Fig. S10. Transformation of substrate 8 by *E. coli* BL21(DE3) containing RrUGT3. i, standard substrate **8**; ii, biotransformation. The conversion efficiency is 85.15%.

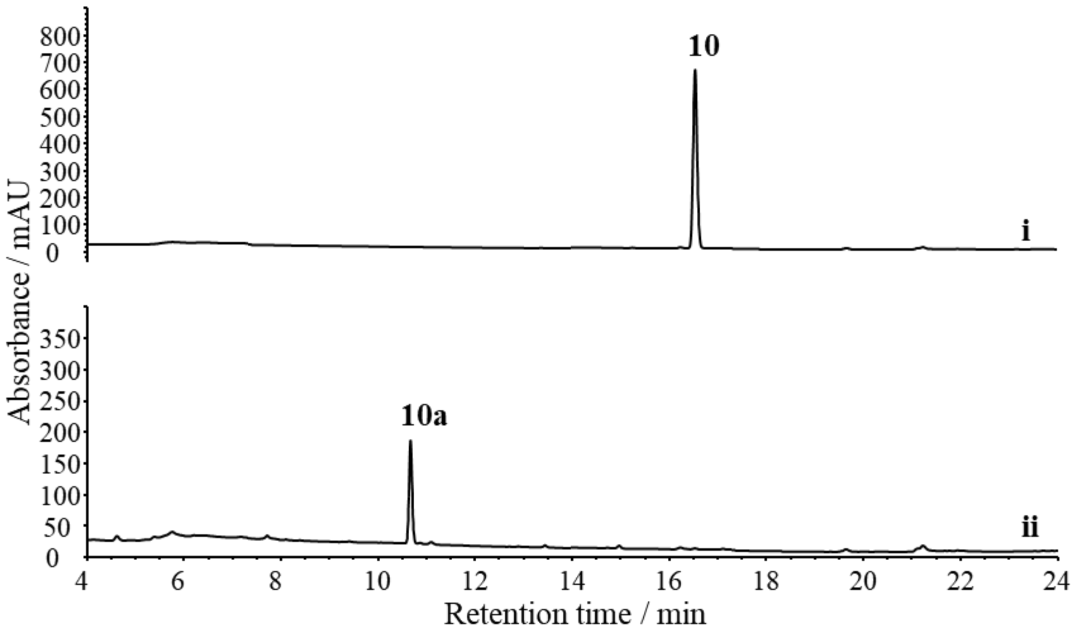

Fig. S11. Transformation of substrate 10 by *E. coli* BL21(DE3) containing RrUGT3. i, standard substrate **10**; ii, biotransformation. The conversion efficiency is 100%.

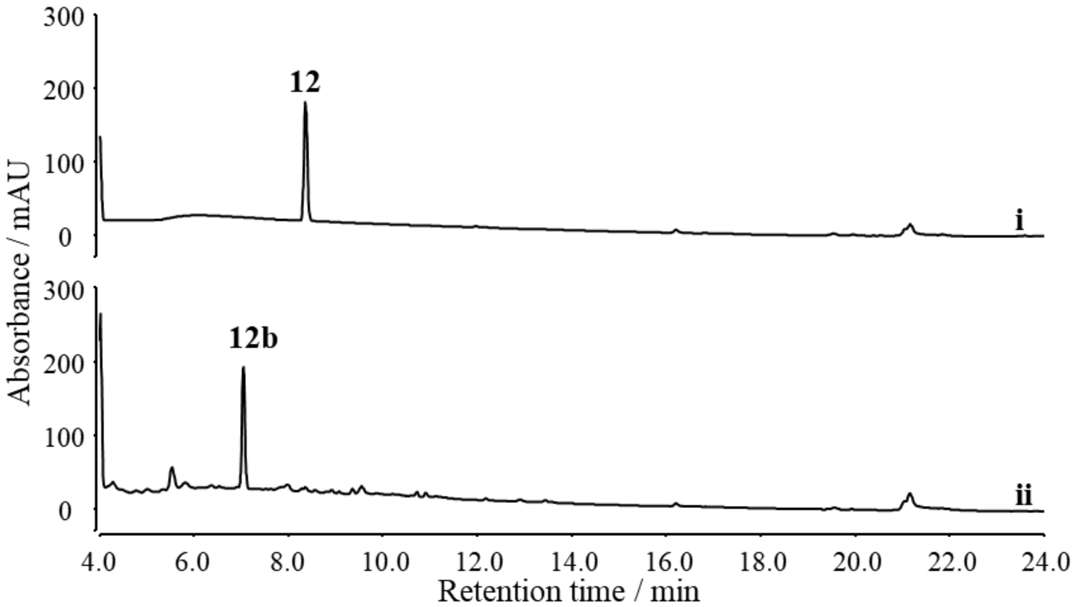

Fig. S12. Transformation of substrate 12 by *E. coli* BL21(DE3) containing RrUGT3. i, standard substrate **12**; ii, biotransformation. The conversion efficiency is 100%.

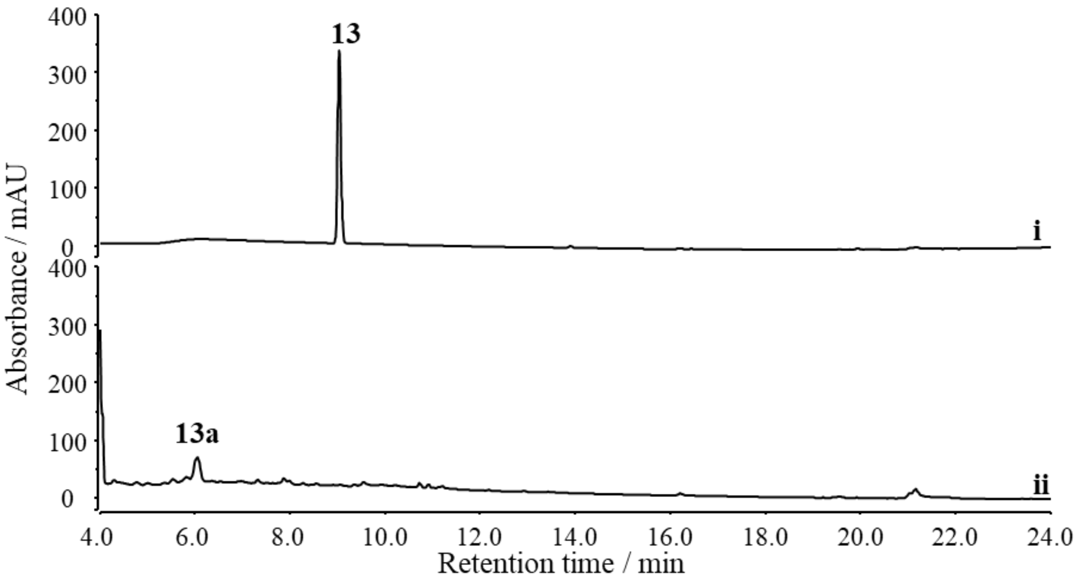

Fig. S13. Transformation of substrate 13 by *E. coli* BL21(DE3) containing RrUGT3. i, standard substrate **13**; ii, biotransformation. The conversion efficiency is 100%.

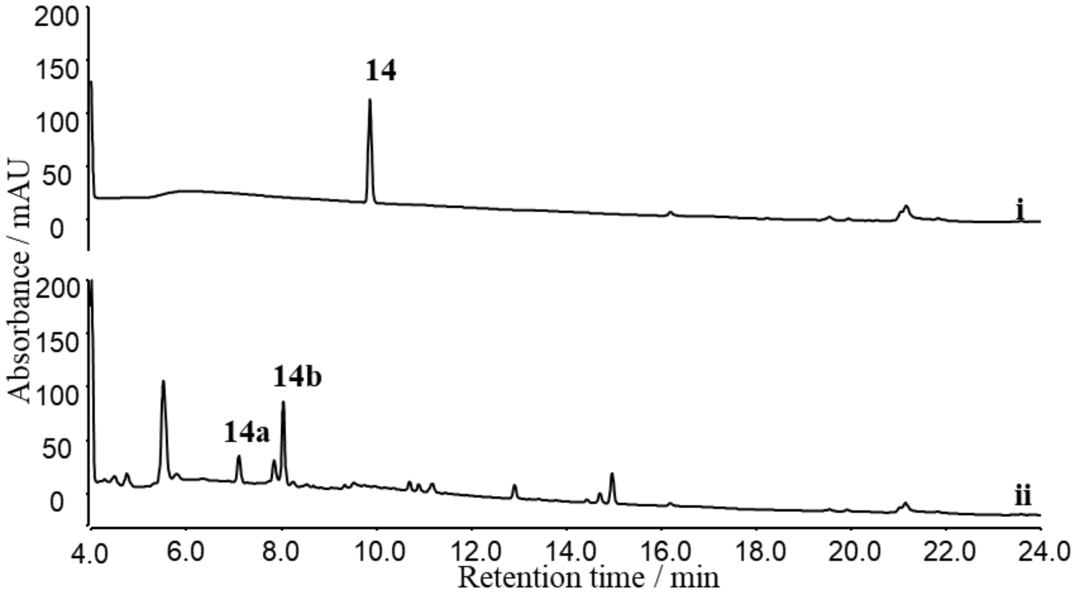

Fig. S14. Transformation of substrate 14 by *E. coli* BL21(DE3) containing RrUGT3. i, standard substrate **14**; ii, biotransformation. The conversion efficiency is 100%. Product **14a** accounted for 50.4%, and product **14b** accounted for 49.6%.

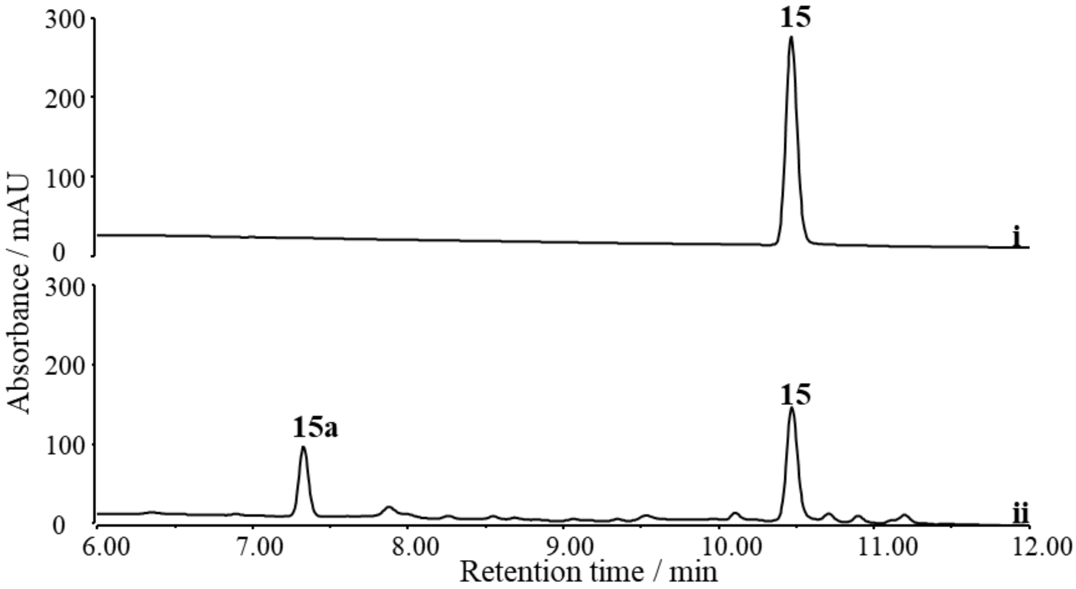

Fig. S15. Transformation of substrate 15 by *E. coli* BL21(DE3) containing RrUGT3. i, standard substrate **15**; ii, biotransformation. The conversion efficiency is 45.75%.

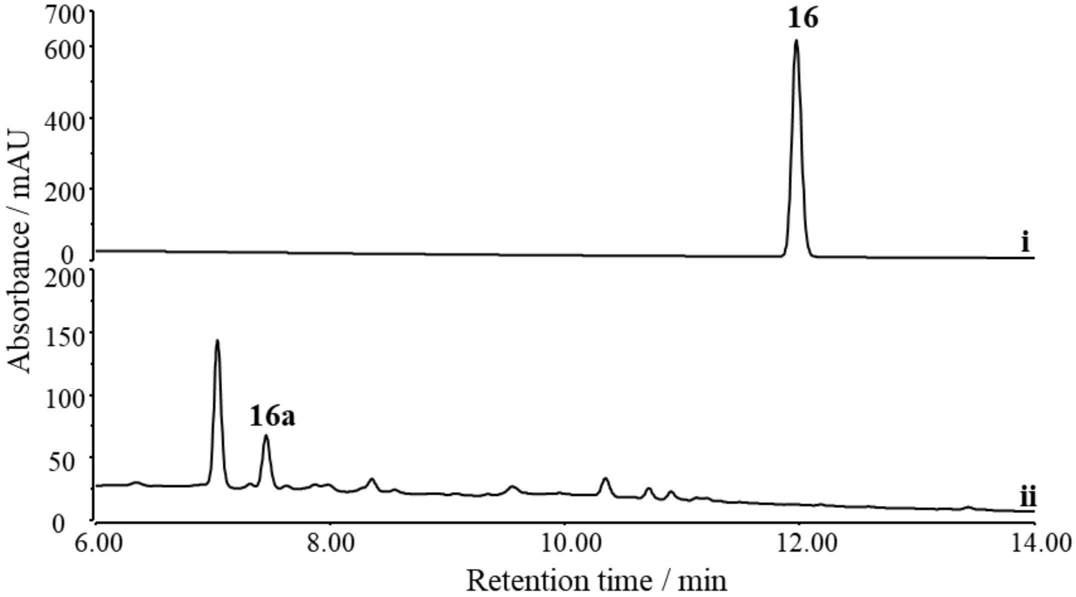

Fig. S16. Transformation of substrate 16 by *E. coli* BL21(DE3) containing RrUGT3. i, standard substrate **16**; ii, biotransformation. The conversion efficiency is 100%.

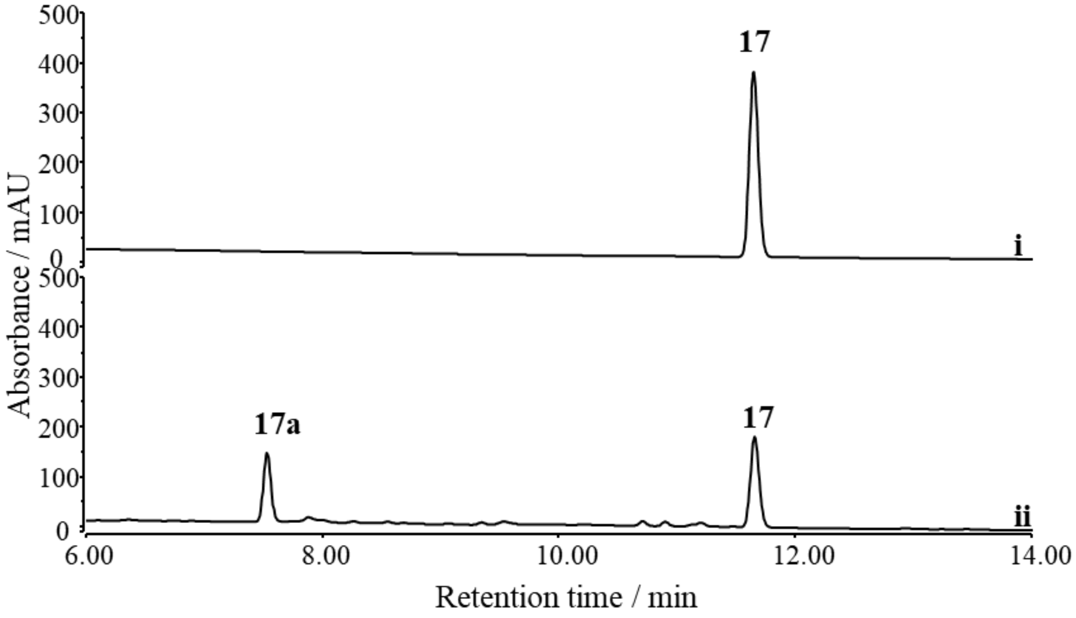

Fig. S17. Transformation of substrate 17 by *E. coli* BL21(DE3) containing RrUGT3. i, standard substrate **17**; ii, biotransformation. The conversion efficiency is 52.4%.

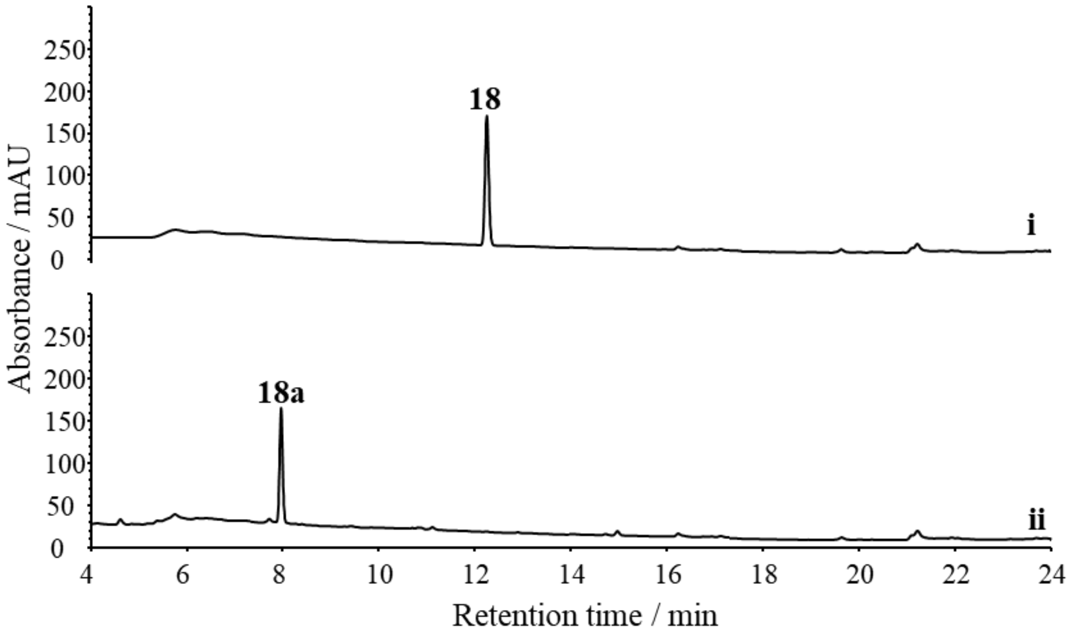

Fig. S18. Transformation of substrate 18 by *E. coli* BL21(DE3) containing RrUGT3. i, standard substrate **18**; ii, biotransformation. The conversion efficiency is 100%.

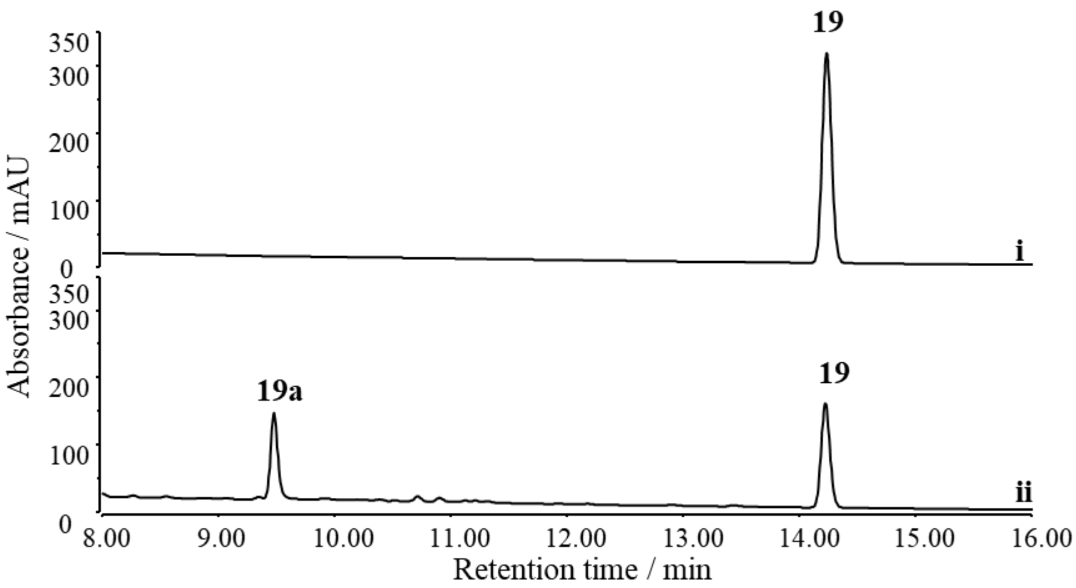

Fig. S19. Transformation of substrate 19 by *E. coli* BL21(DE3) containing RrUGT3. i, standard substrate **19**; ii, biotransformation. The conversion efficiency is 43.55%.

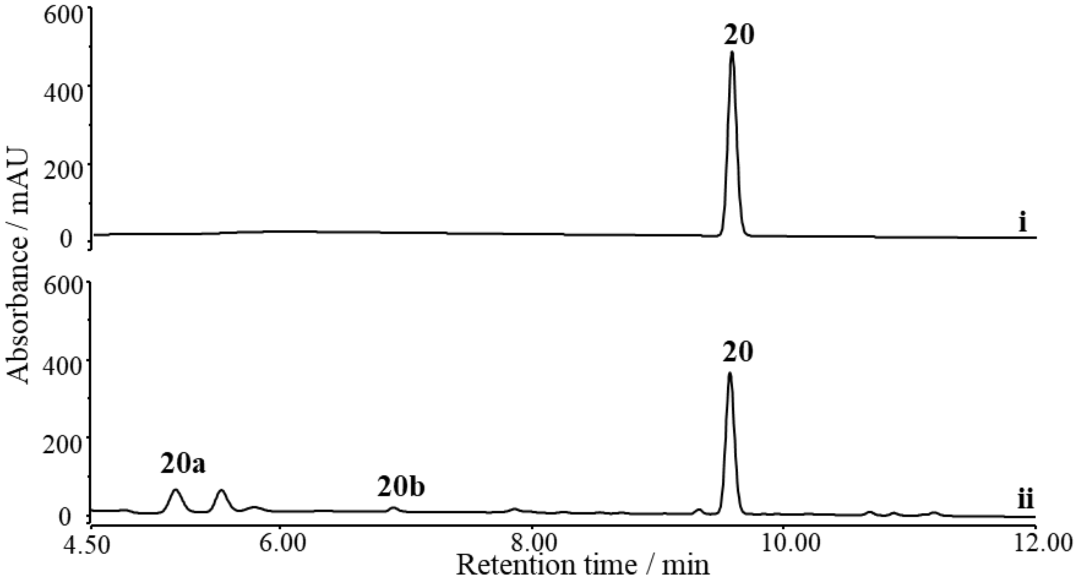

Fig. S20. Transformation of substrate 20 by *E. coli* BL21(DE3) containing RrUGT3. i, standard substrate **20**; ii, biotransformation. The conversion efficiency is 19.5%. Product **20a** accounted for 15.88%, and product **20b** accounted for 3.62%.

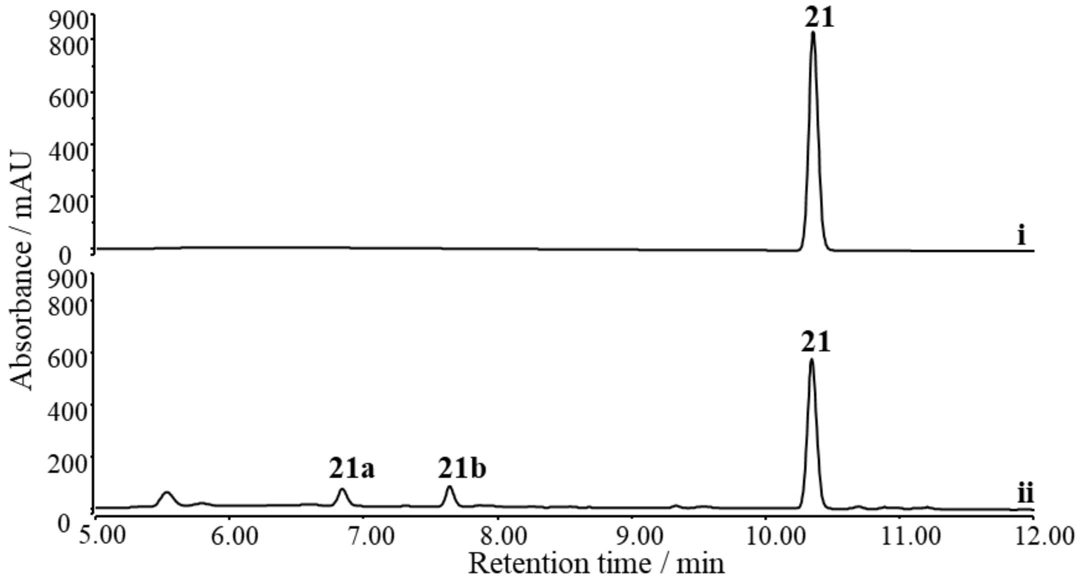

Fig. S21. Transformation of substrate 21 by *E. coli* BL21(DE3) containing RrUGT3. i, standard substrate **21**; ii, biotransformation. The conversion efficiency is 30.54%. Product **21a** accounted for 18.39%, and product **21b** accounted for 12.15%.

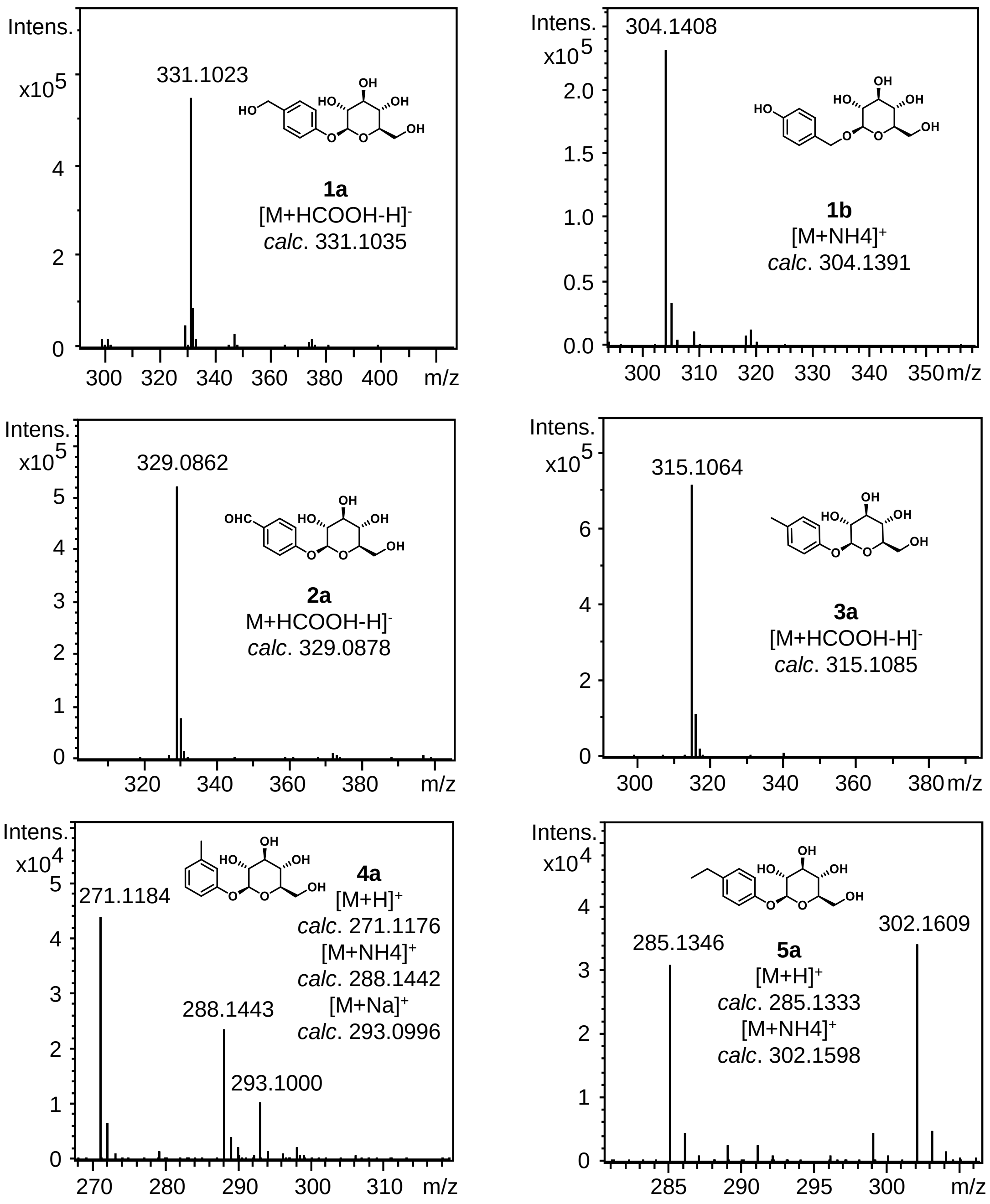

Fig. S22. HRMS spectra of glucosides.

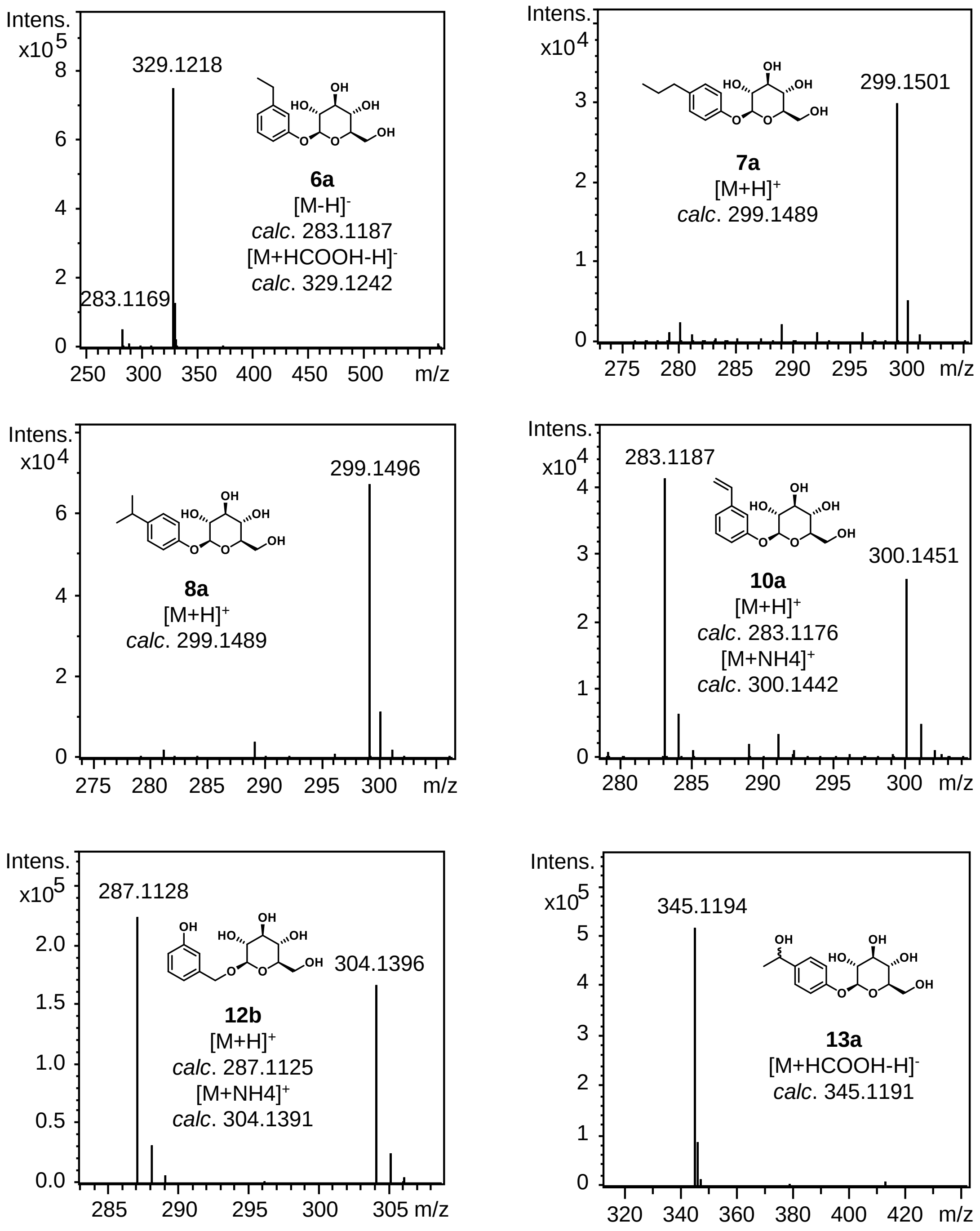

Continue of Fig. S22.

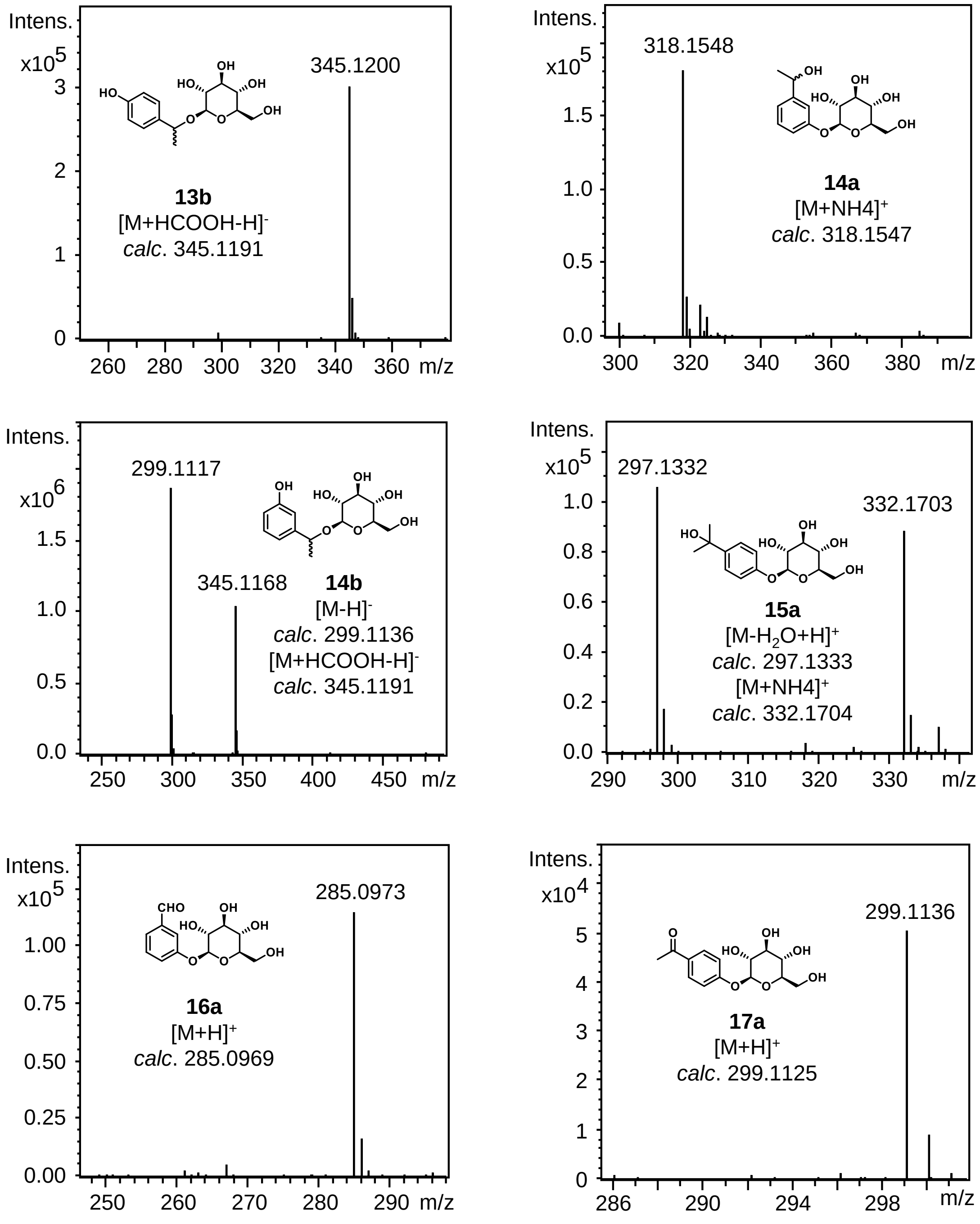

Continue of Fig. S22.

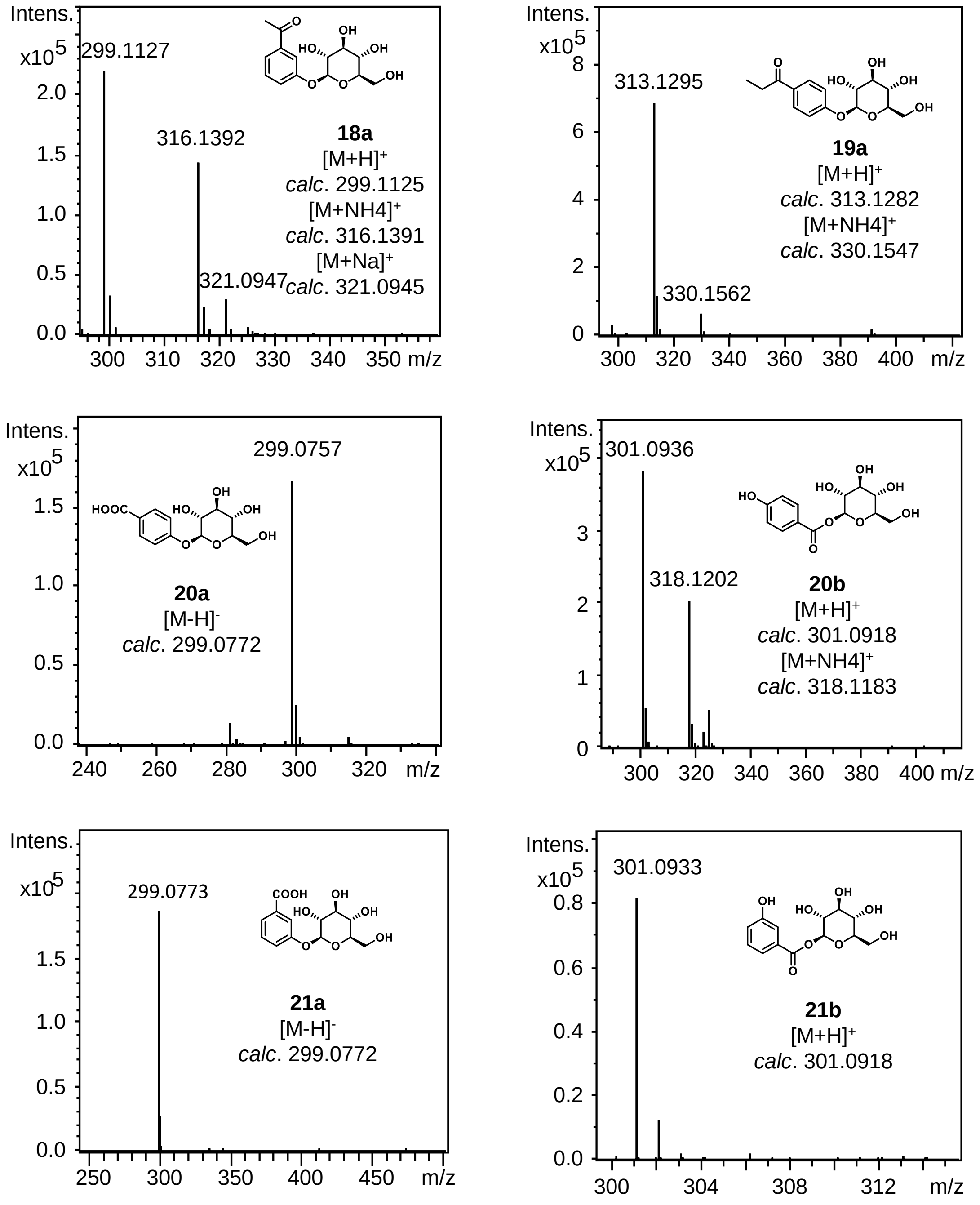

Continue of Fig. S22.

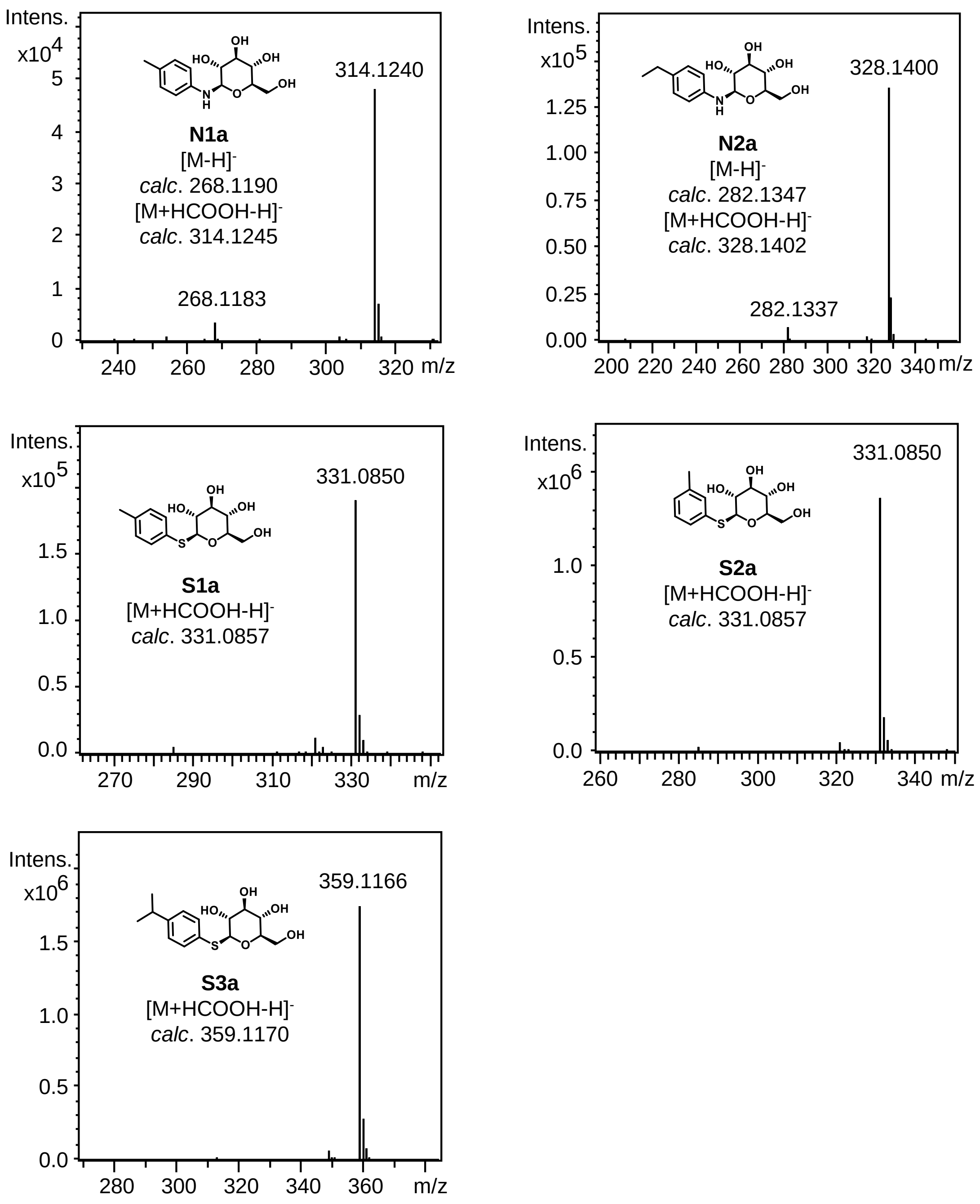

Continue of Fig. S22.

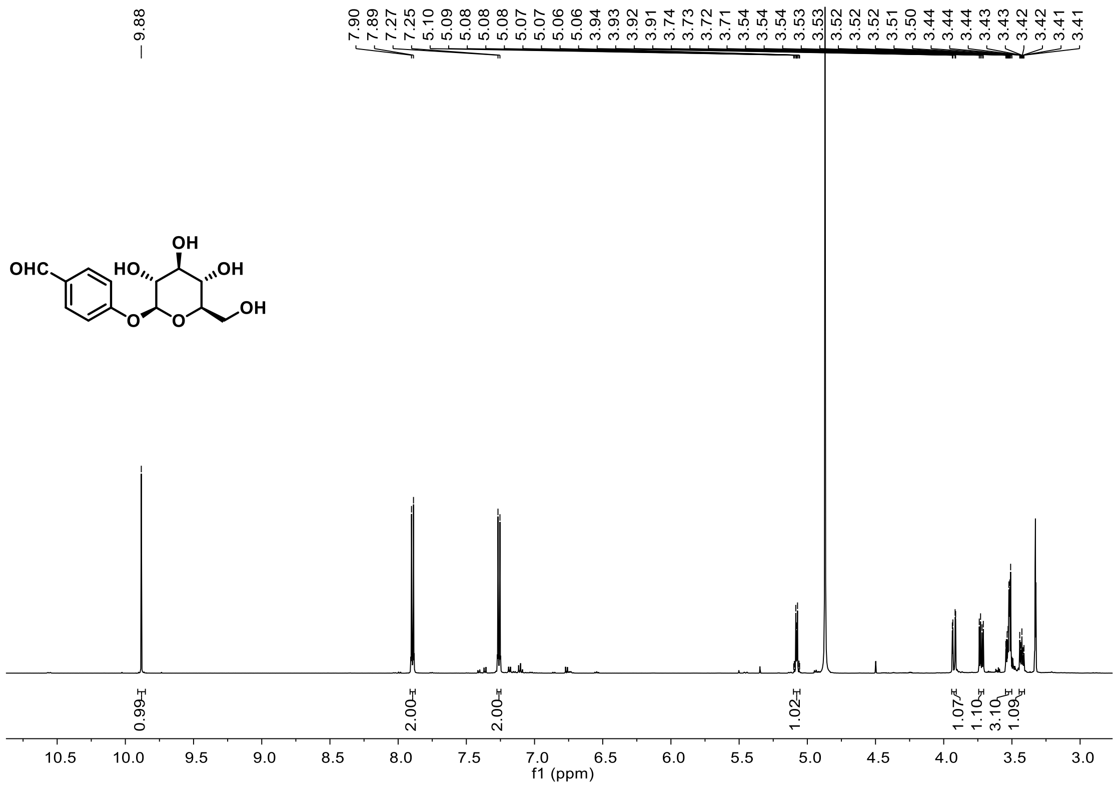

Fig. S23. [1]**H NMR spectrum of 2a in CD3OD.**

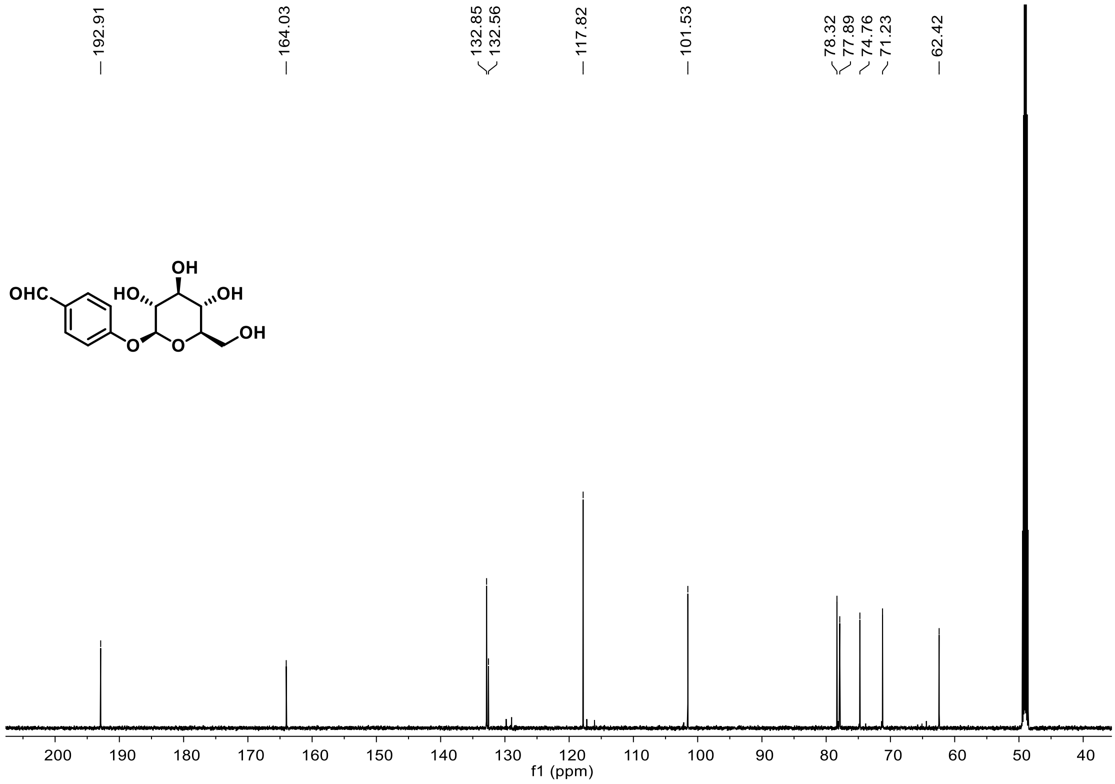

Fig. S24. [13]**C NMR spectrum of 2a in CD3OD.**

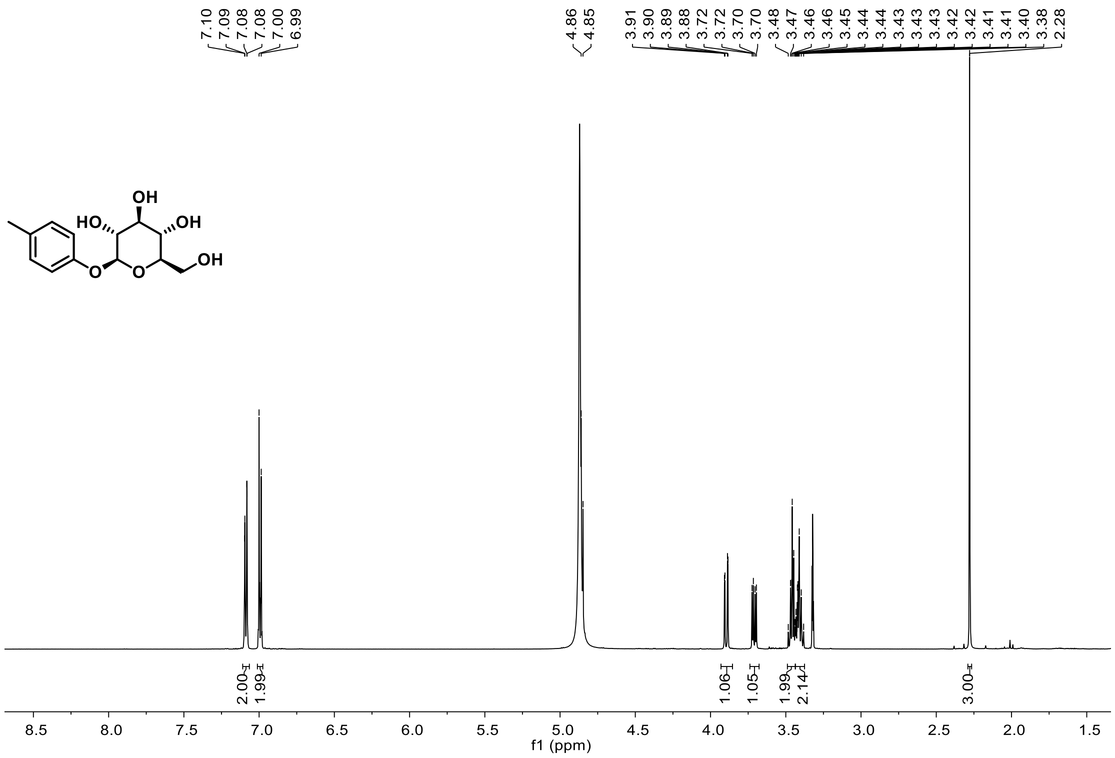

Fig. S25. ¹H NMR spectrum of 3a in CD_3OD.

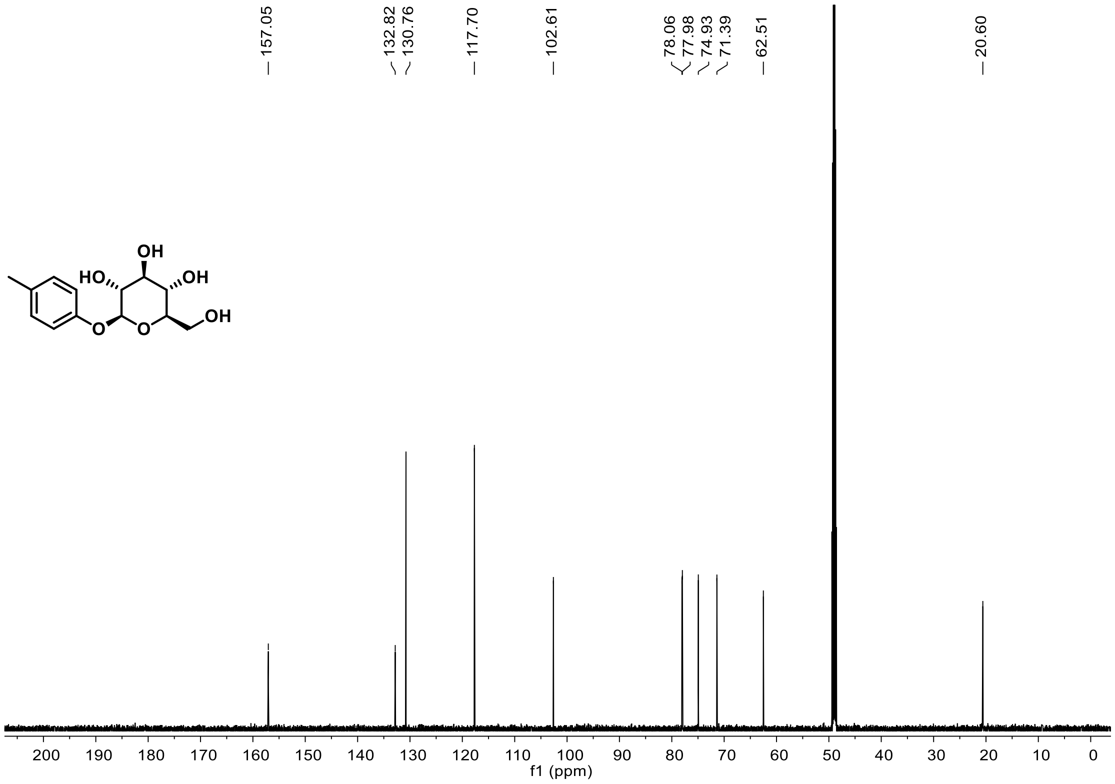

Fig. S26. ¹³C NMR spectrum of 3a in CD_3OD.

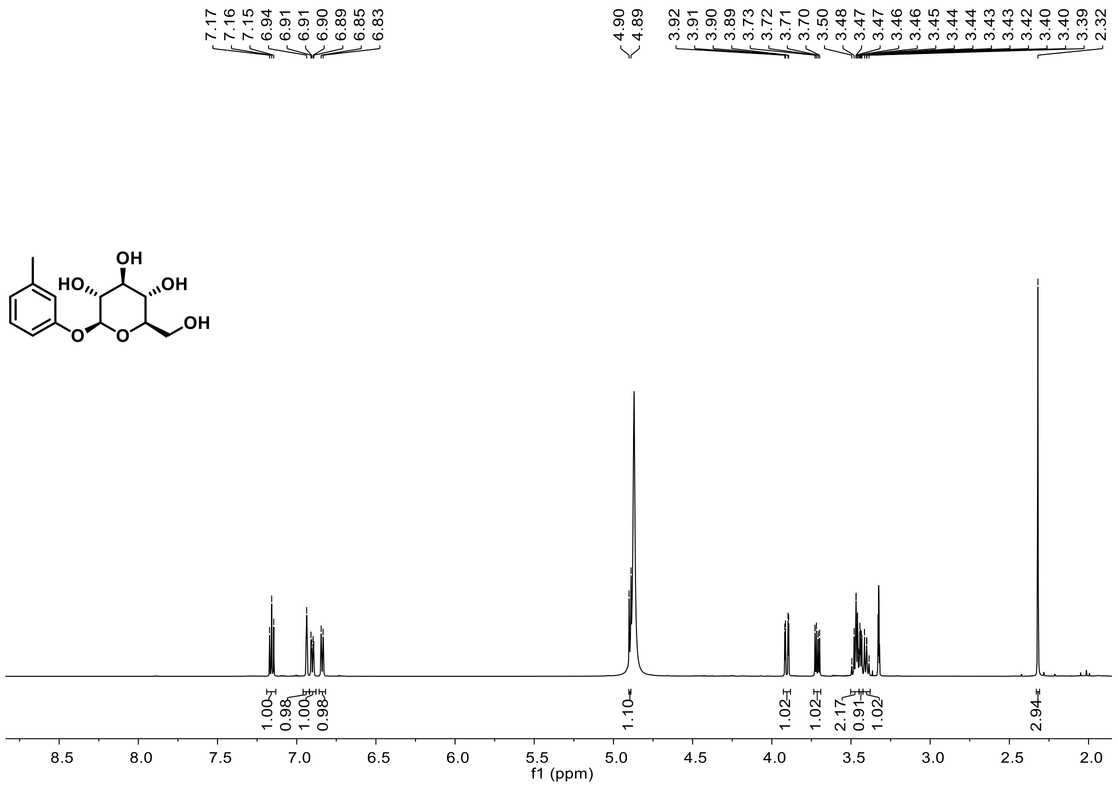

Fig. S27. ^{1}**H NMR spectrum of 4a in** CD_3OD**.**

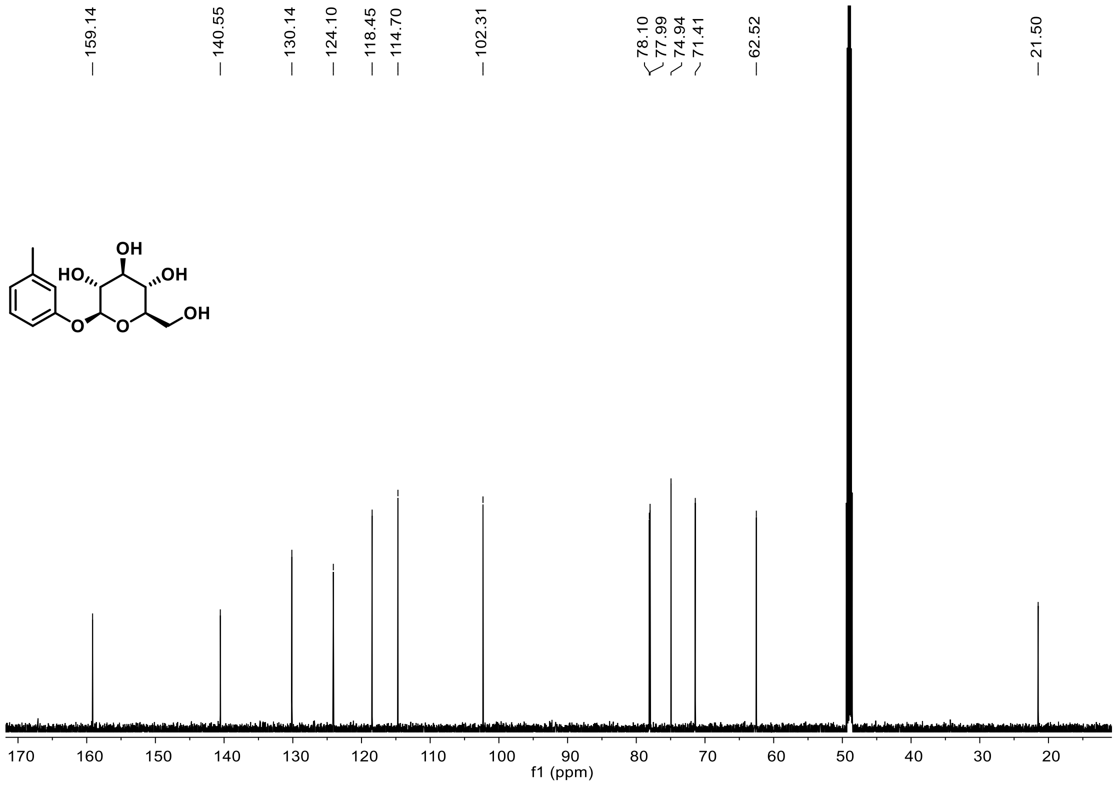

Fig. S28. ^{13}**C NMR spectrum of 4a in** CD_3OD**.**

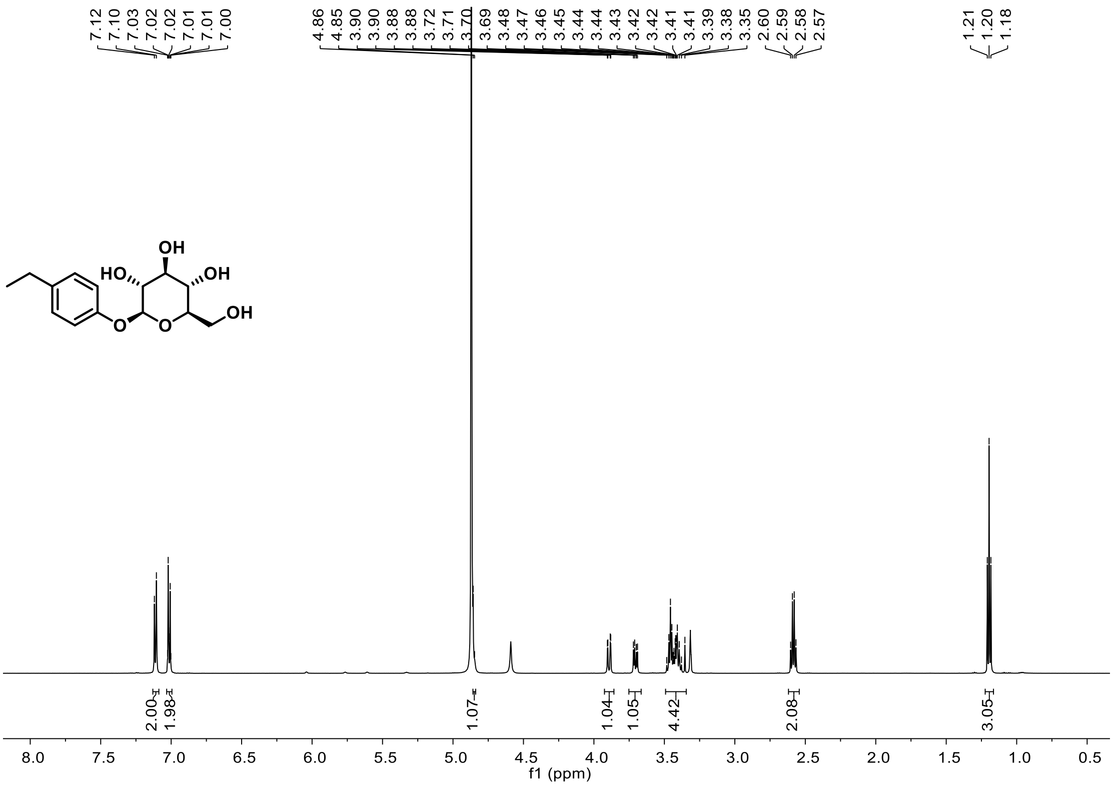

Fig. S29. ^{1}H NMR spectrum of 5a in CD_3OD.

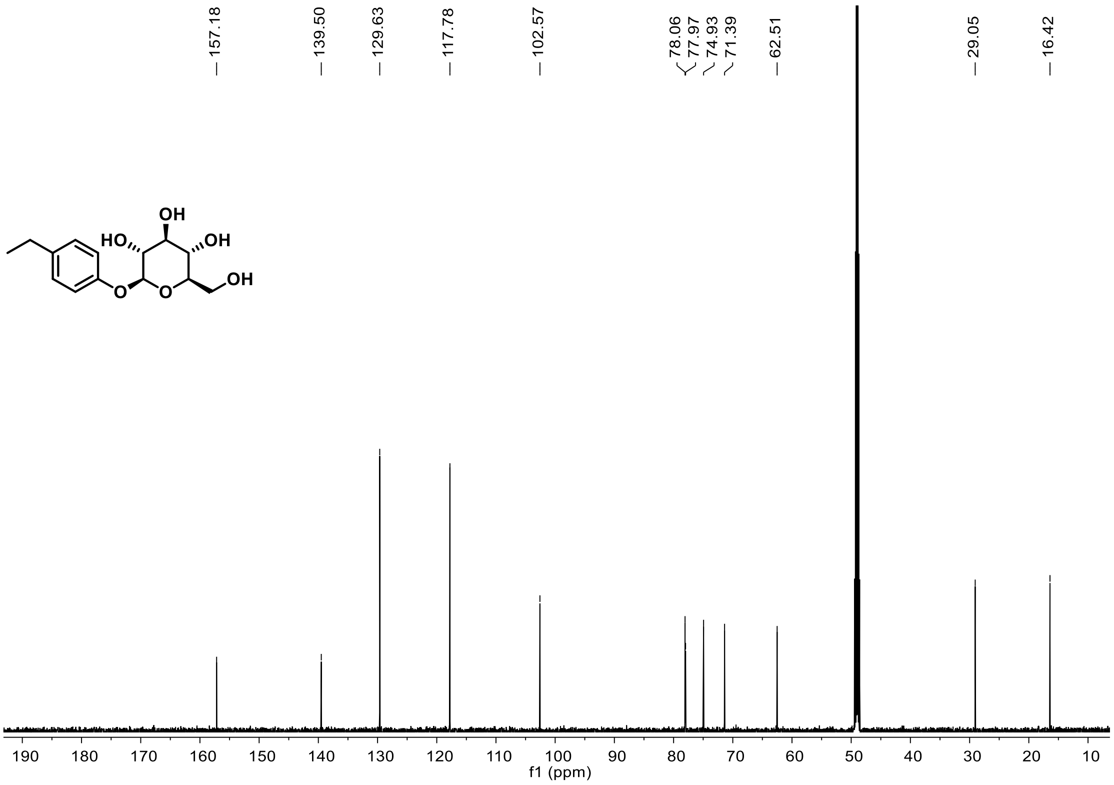

Fig. S30. ^{13}C NMR spectrum of 5a in CD_3OD.

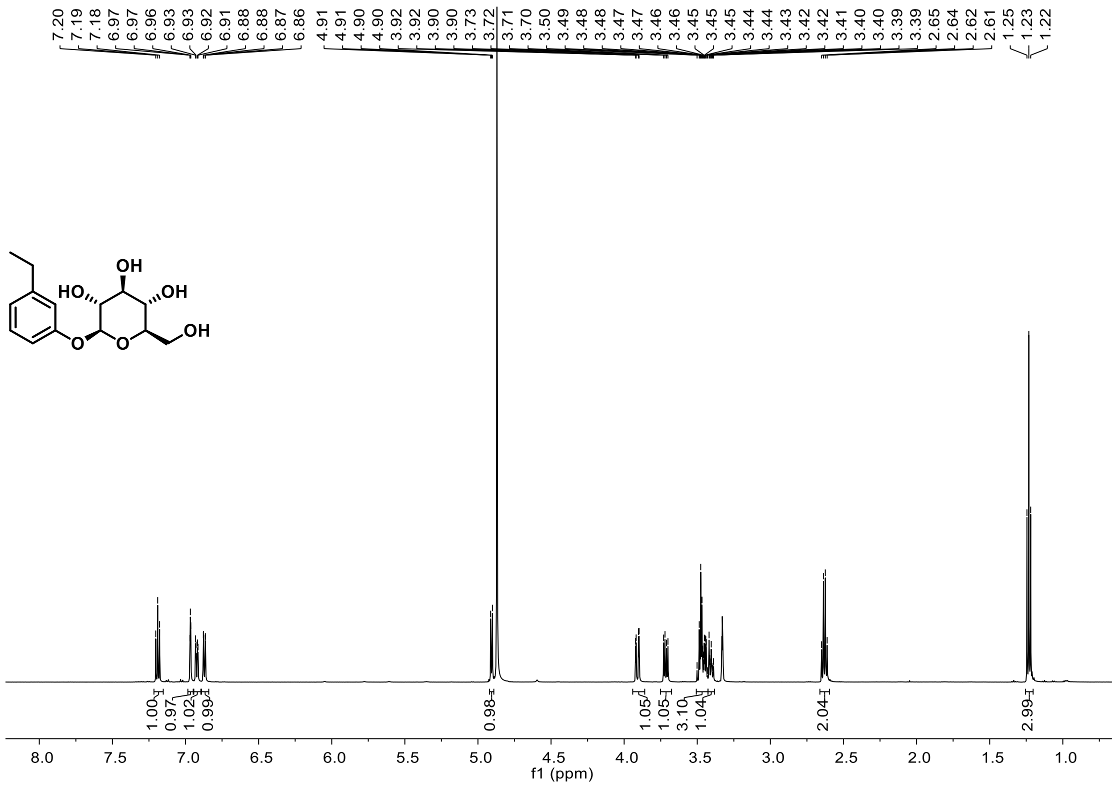

Fig. S31. ^{1}H NMR spectrum of 6a in CD3OD.

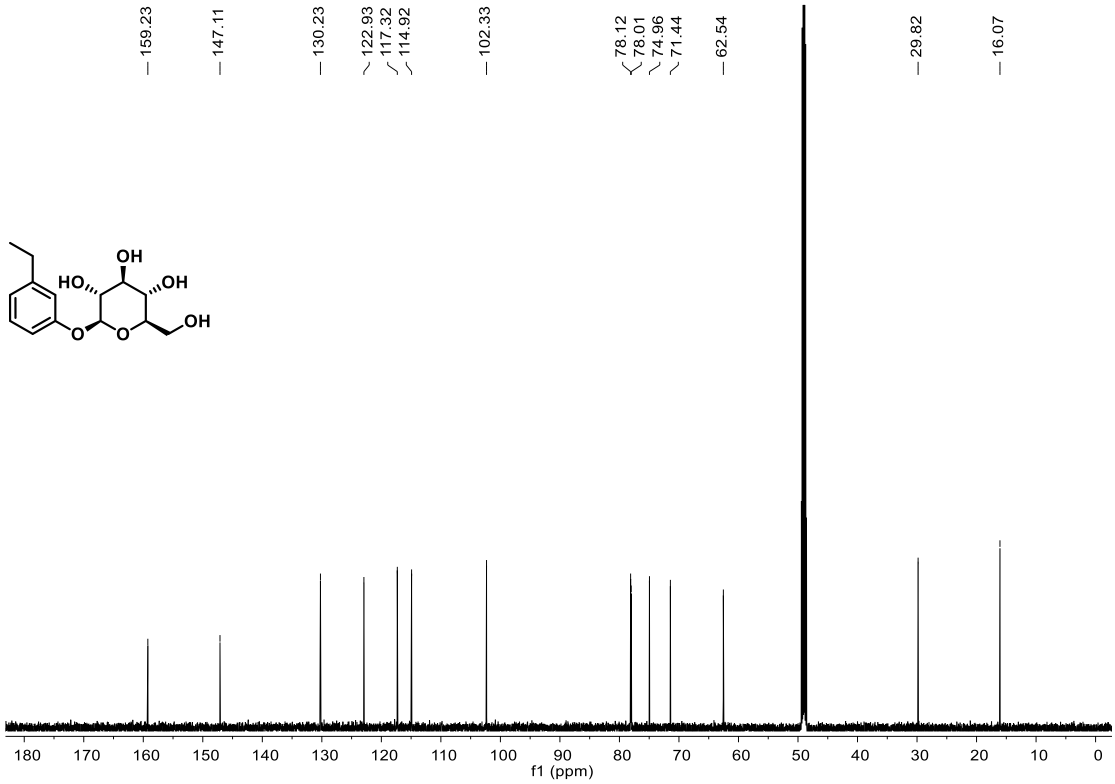

Fig. S32. ^{13}C NMR spectrum of 6a in CD3OD.

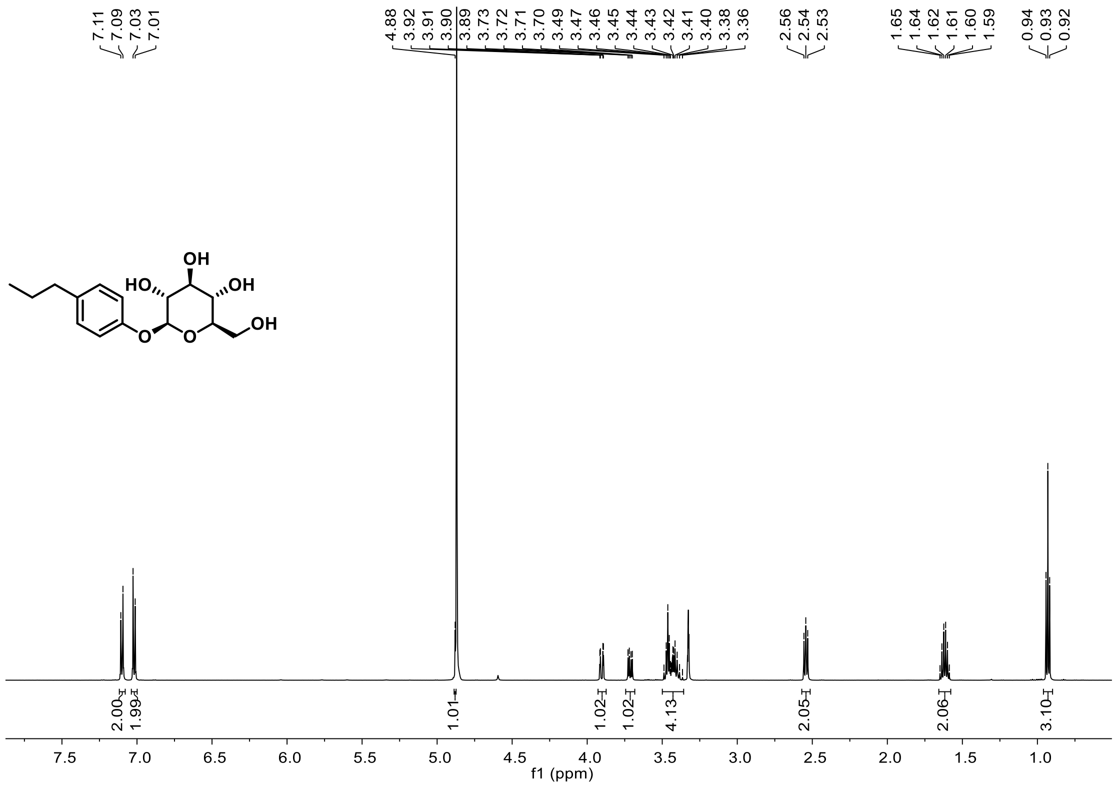

Fig. S33. ^{1}**H NMR spectrum of 7a in CD$_3$OD.**

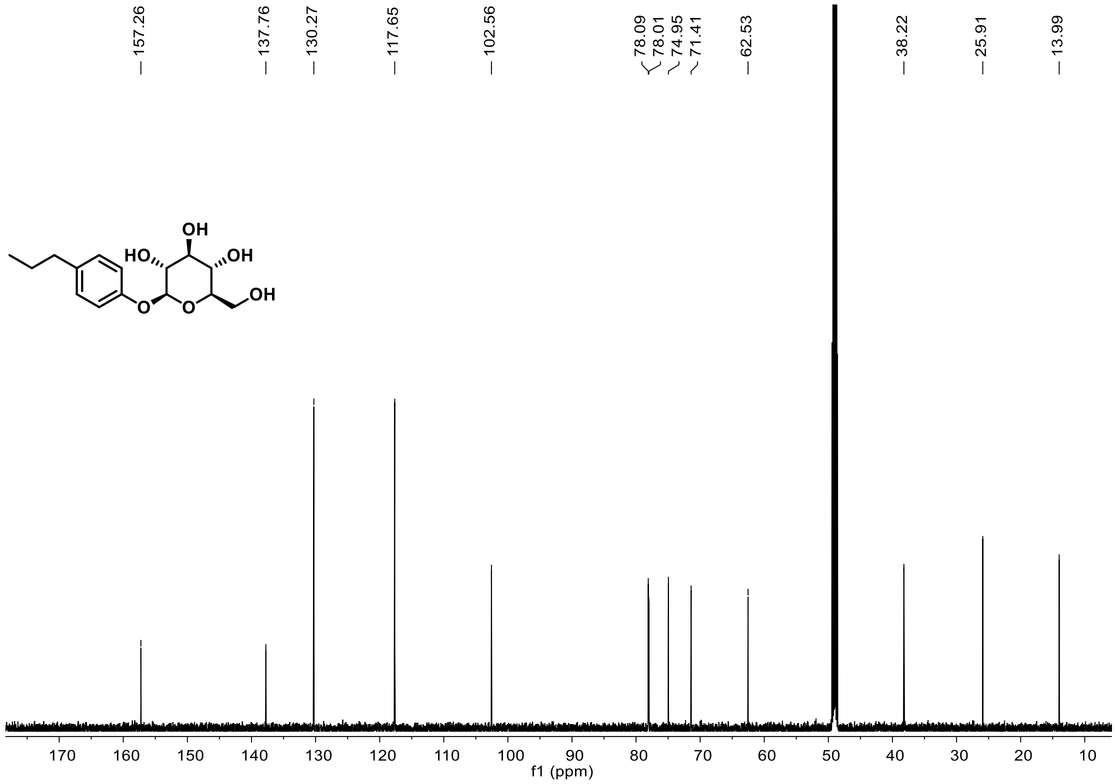

Fig. S34. ^{13}**C NMR spectrum of 7a in CD$_3$OD.**

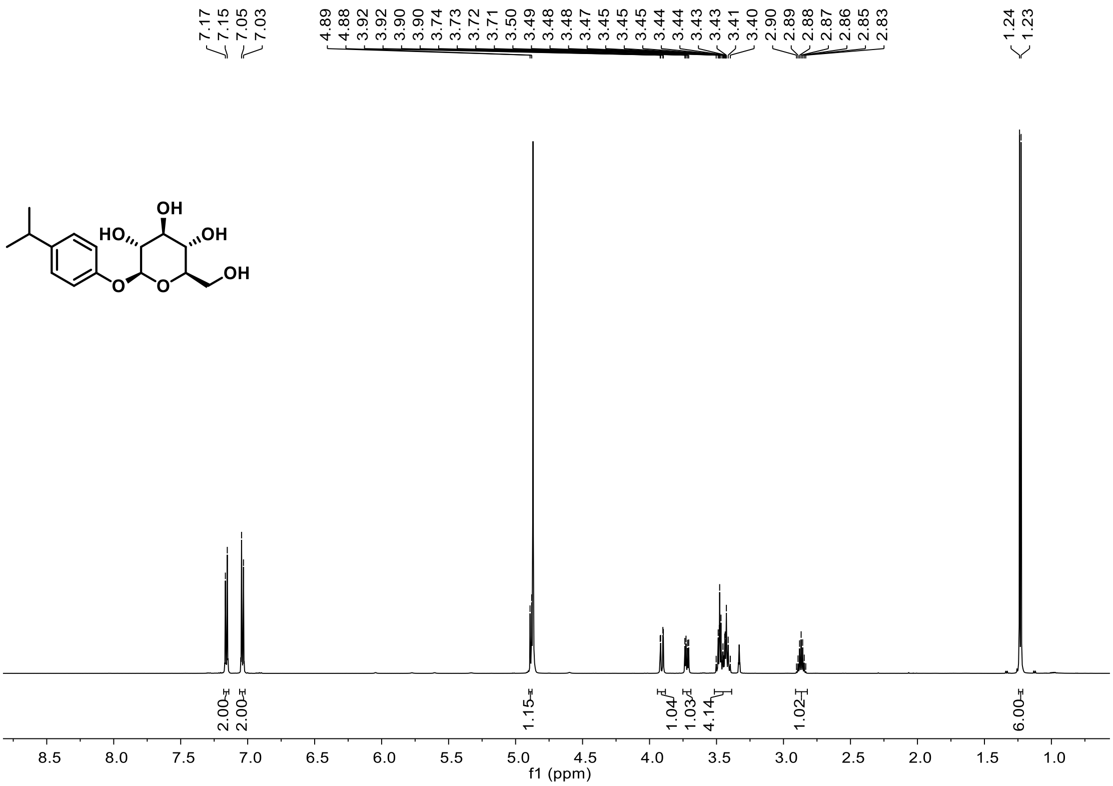

Fig. S35. ^{1}H NMR spectrum of 8a in CD_3OD.

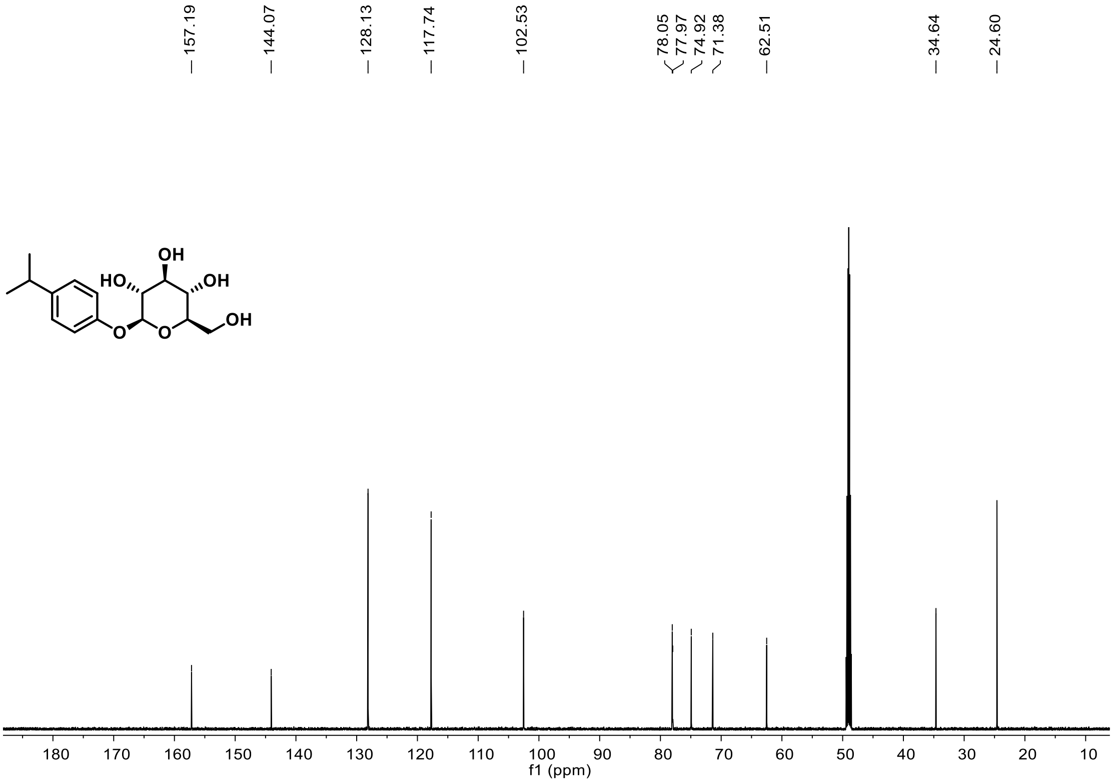

Fig. S36. ^{13}C NMR spectrum of 8a in CD_3OD.

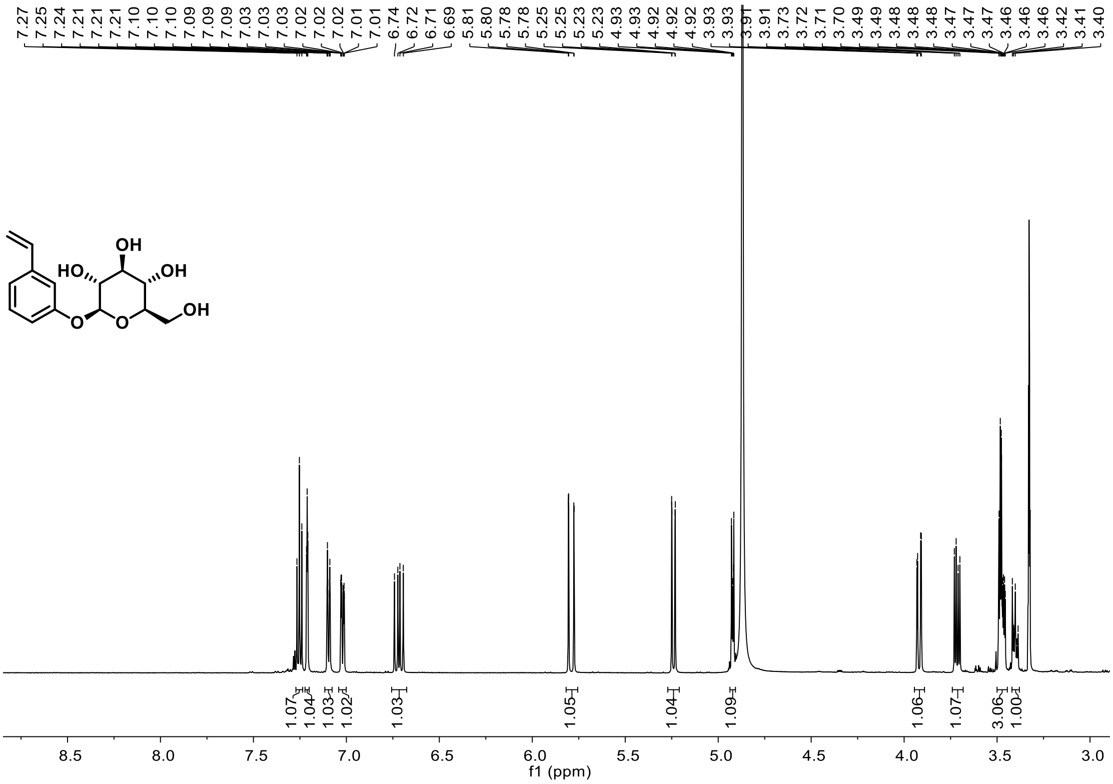

Fig. S37. ^{1}H NMR spectrum of 10a in CD3OD.

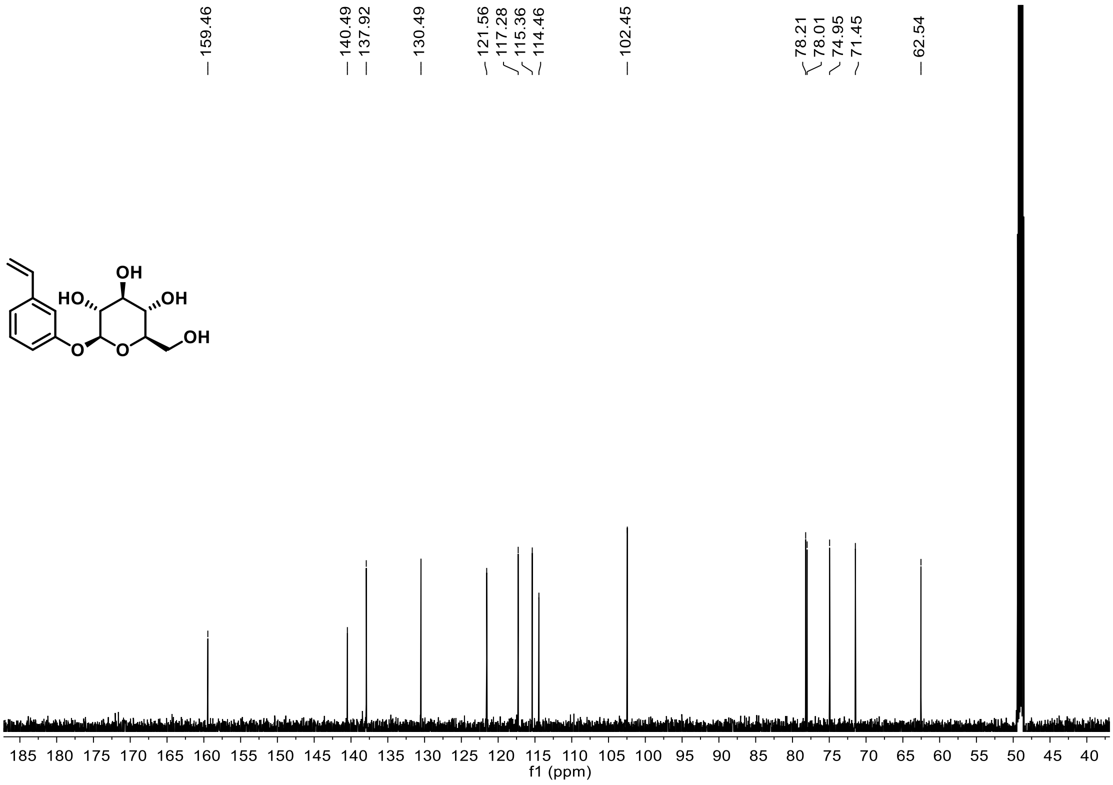

Fig. S38. ^{13}C NMR spectrum of 10a in CD3OD.

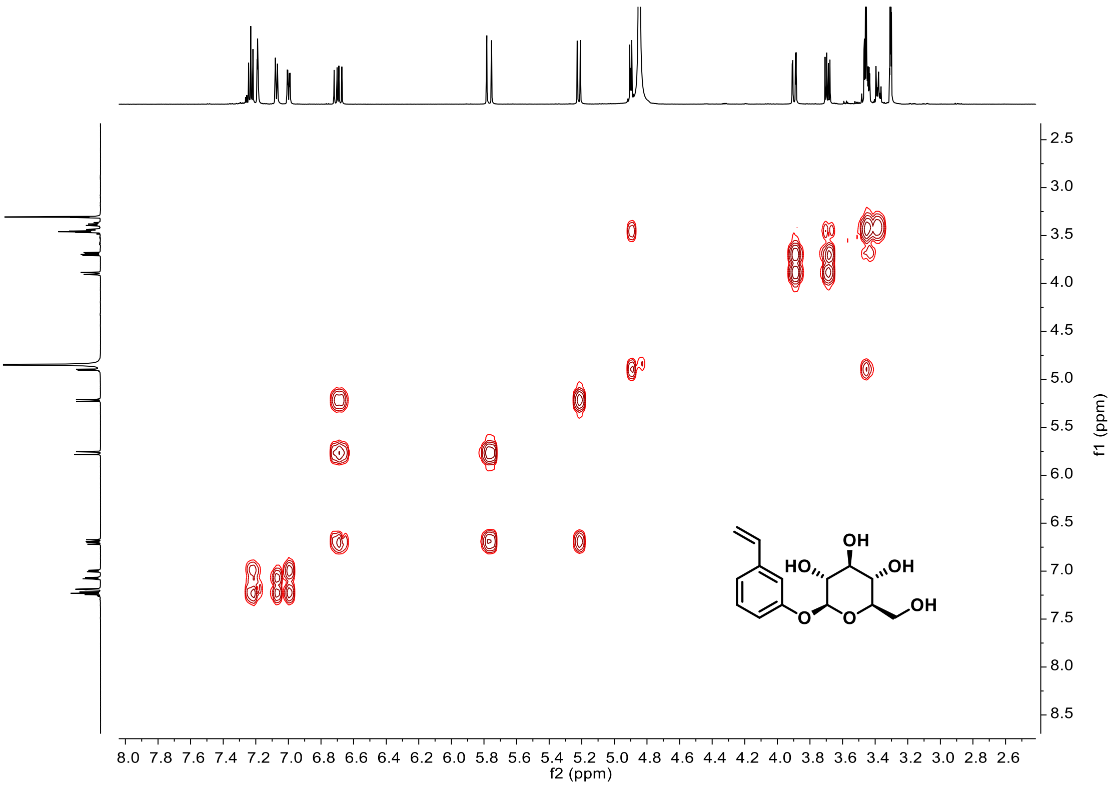

Fig. S39. COSY NMR spectrum of 10a in CD_3OD.

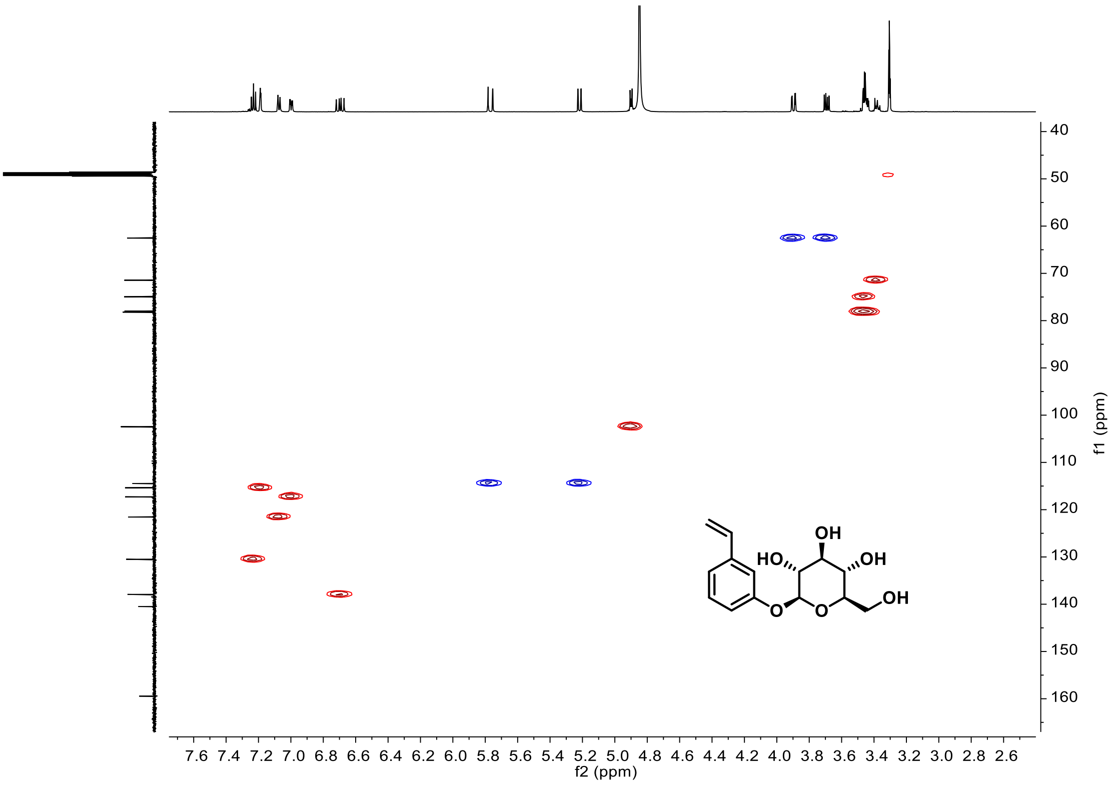

Fig. S40. HSQC NMR spectrum of 10a in CD_3OD.

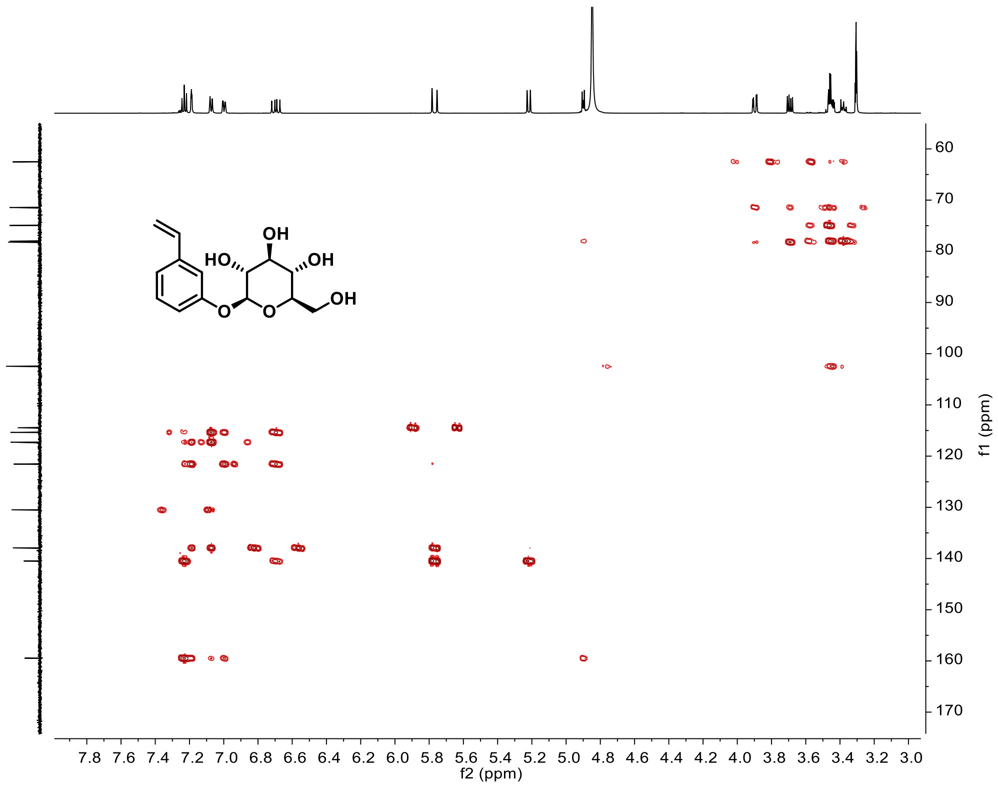

Fig. S41. HMBC NMR spectrum of 10a in CD3OD.

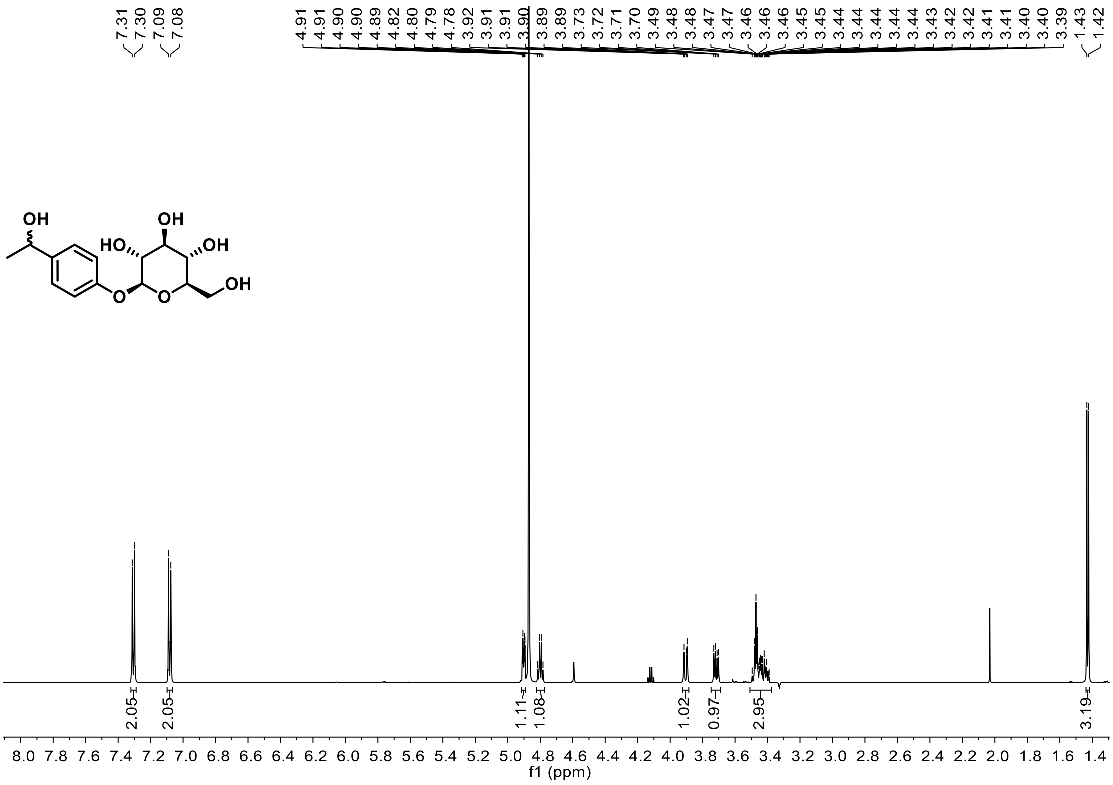

Fig. S42. ^{1}H NMR spectrum of 13a in CD3OD.

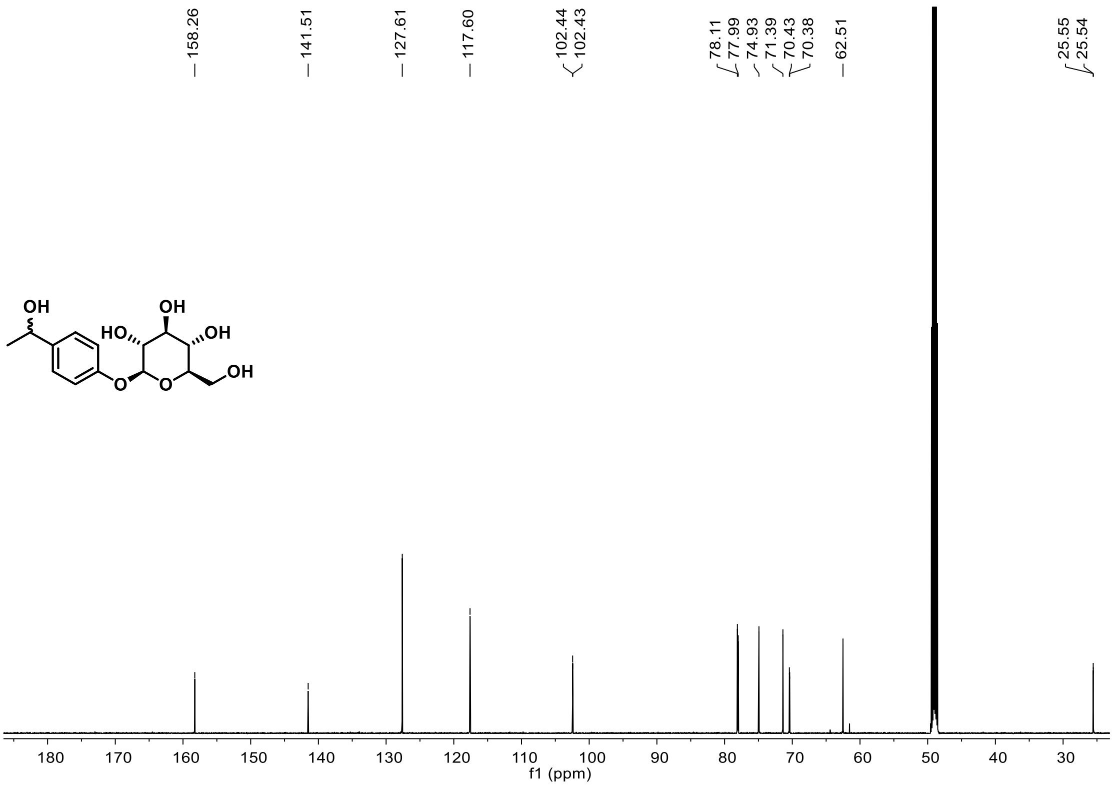

Fig. S43. ^{13}**C NMR spectrum of 13a in CD**$_3$**OD.**

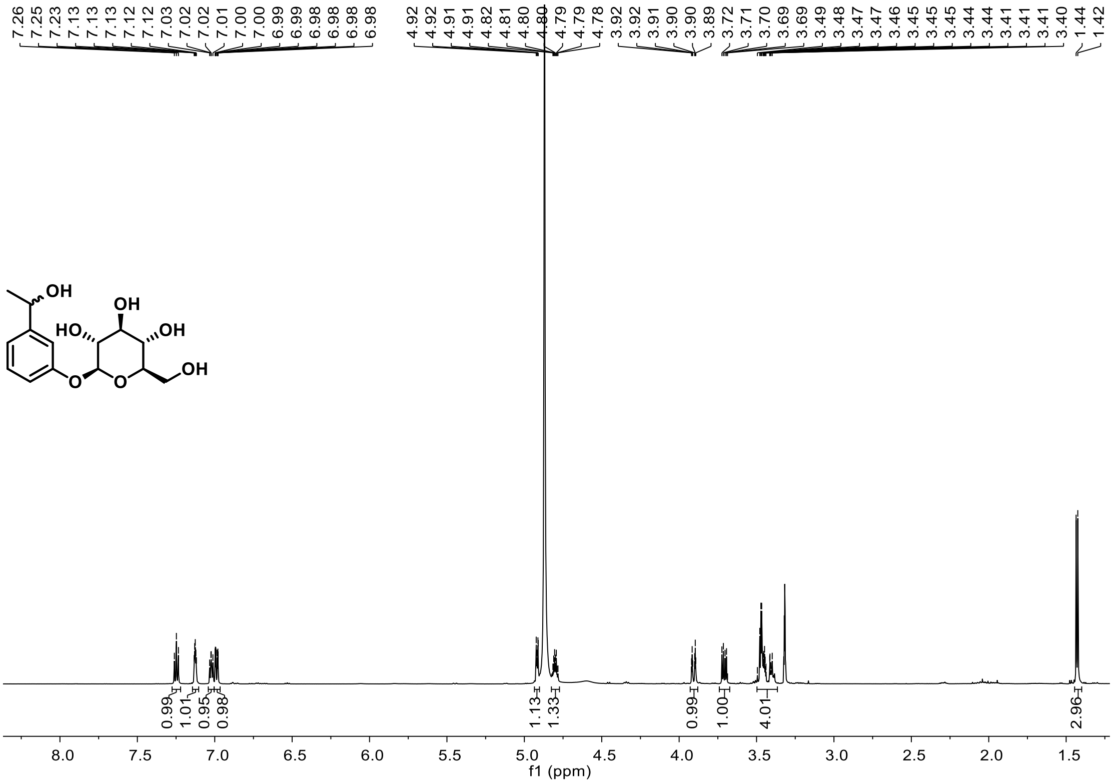

Fig. S44. ^{1}**H NMR spectrum of 14a in CD**$_3$**OD.**

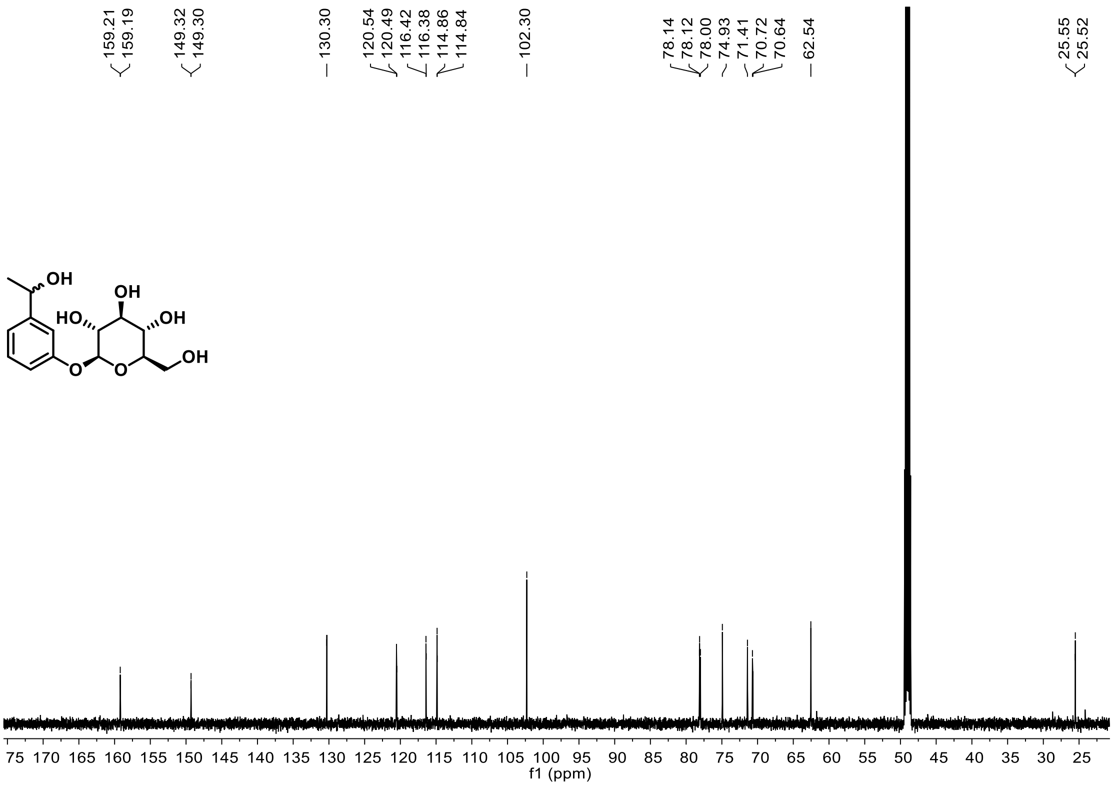

Fig. S45. ^{13}C NMR spectrum of 14a in CD_3OD.

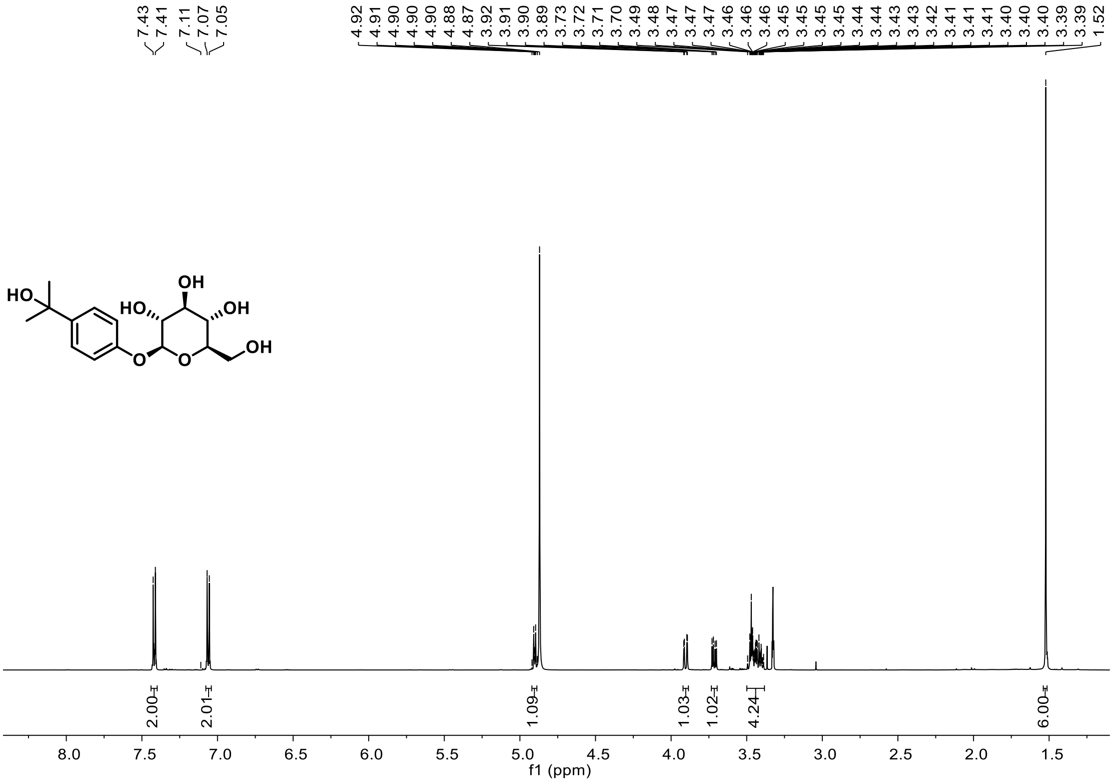

Fig. S46. ^{1}H NMR spectrum of 15a in CD_3OD.

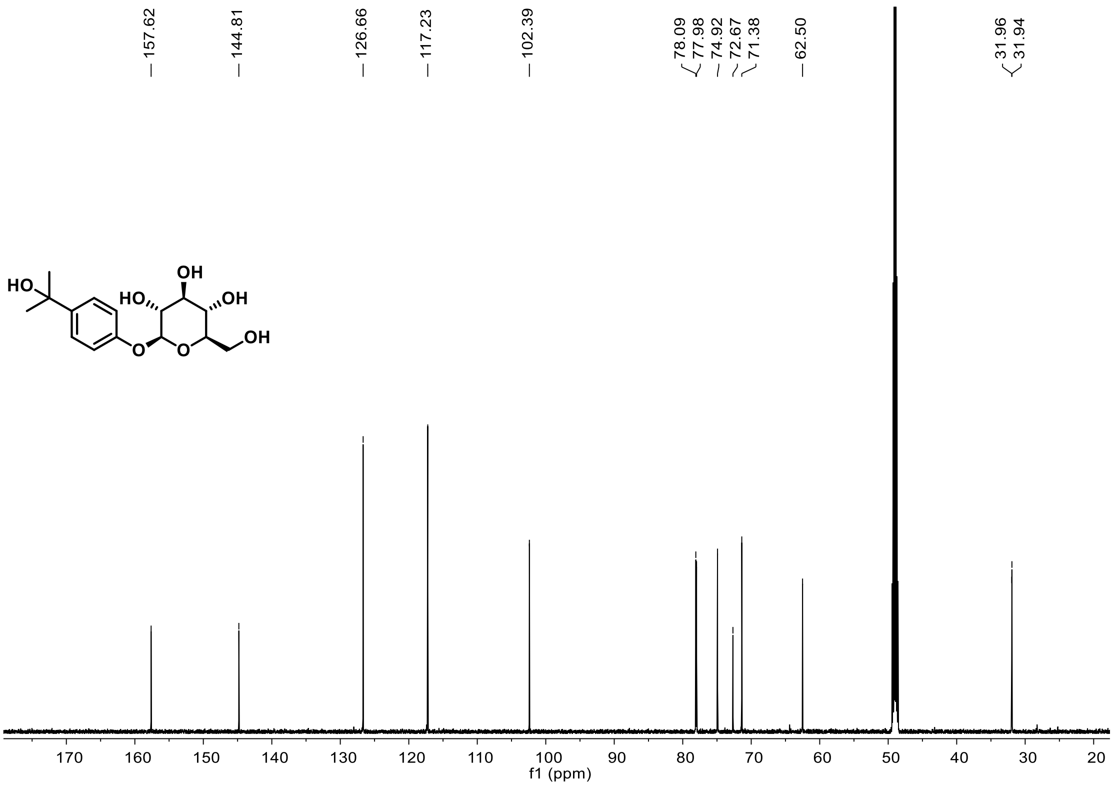

Fig. S47. ^{13}C NMR spectrum of 15a in CD_3OD.

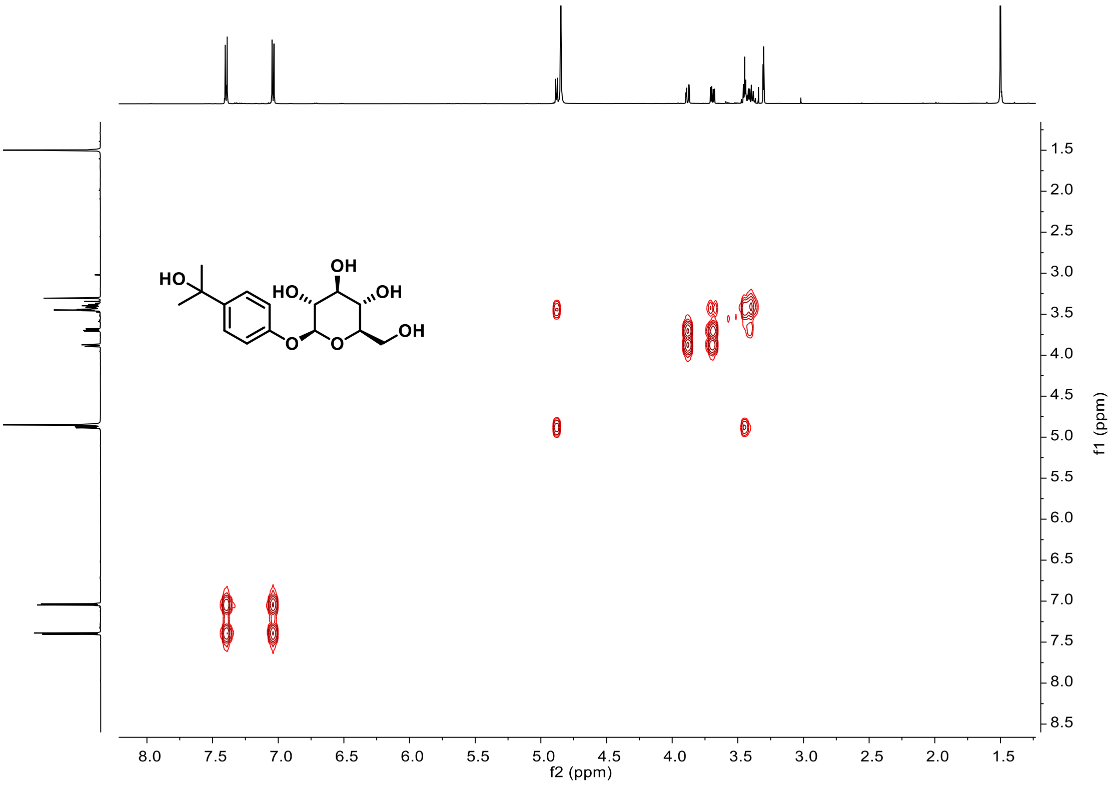

Fig. S48. COSY NMR spectrum of 15a in CD_3OD.

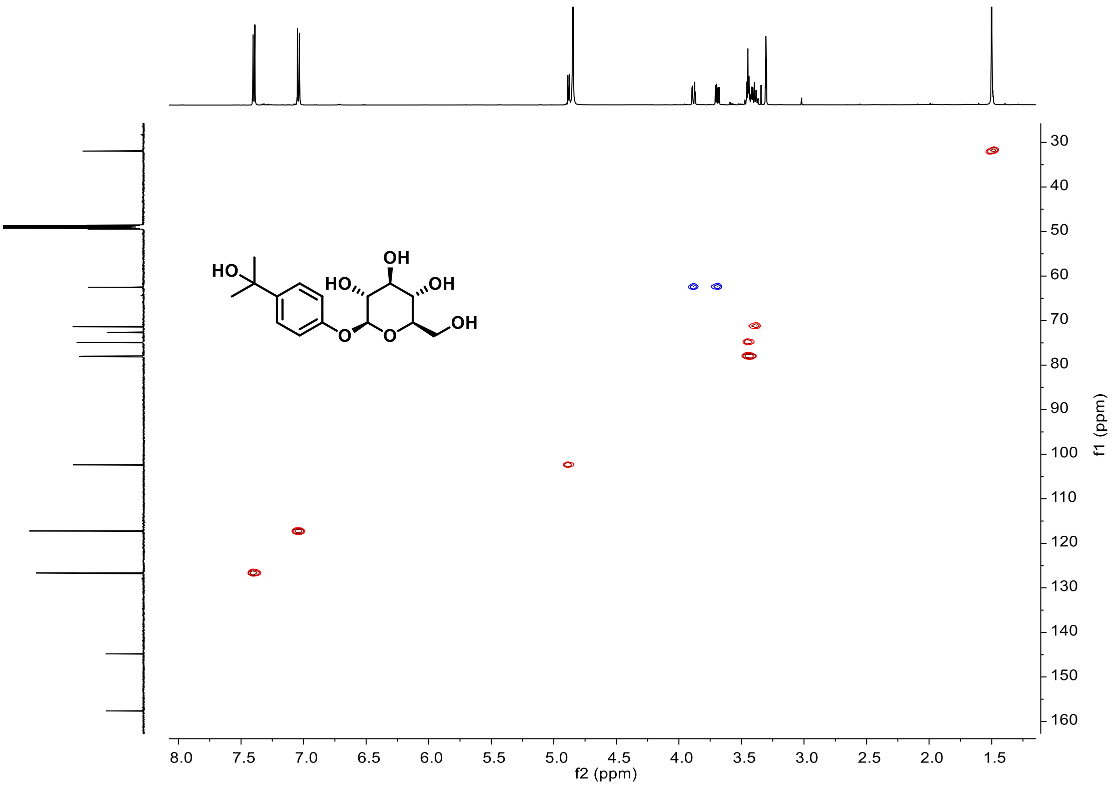

Fig. S49. HSQC NMR spectrum of 15a in CD3OD.

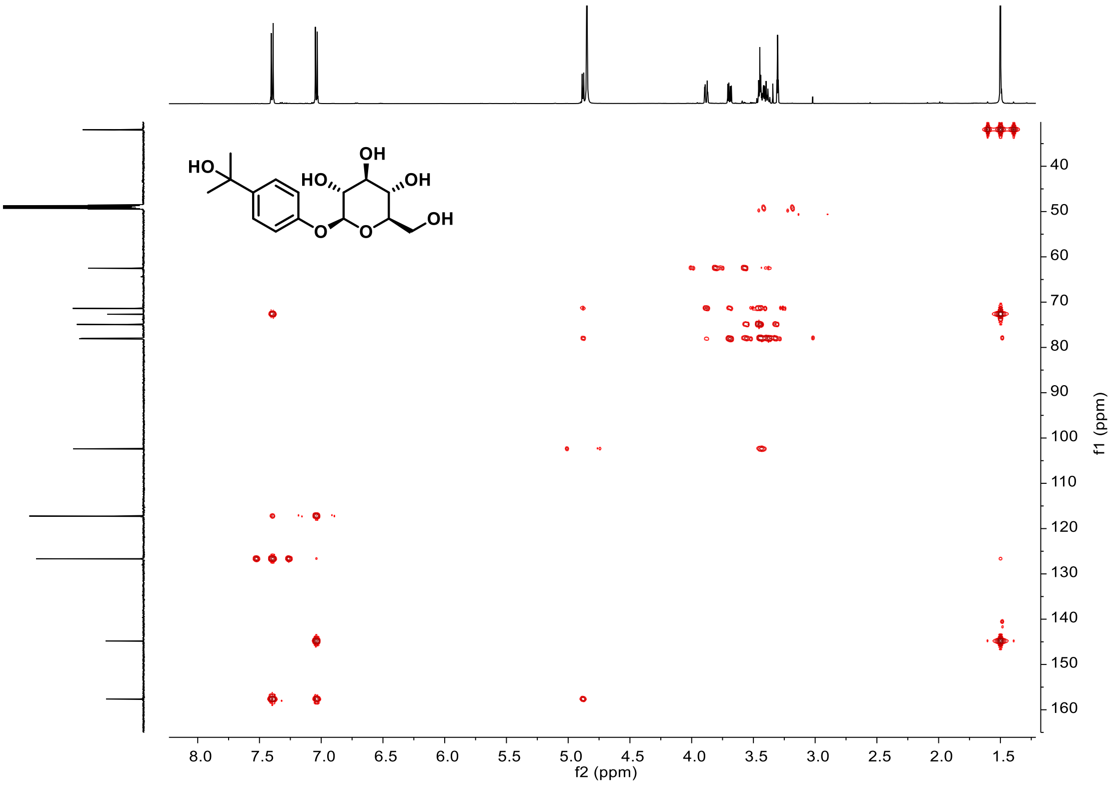

Fig. S50. HMBC NMR spectrum of 15a in CD3OD.

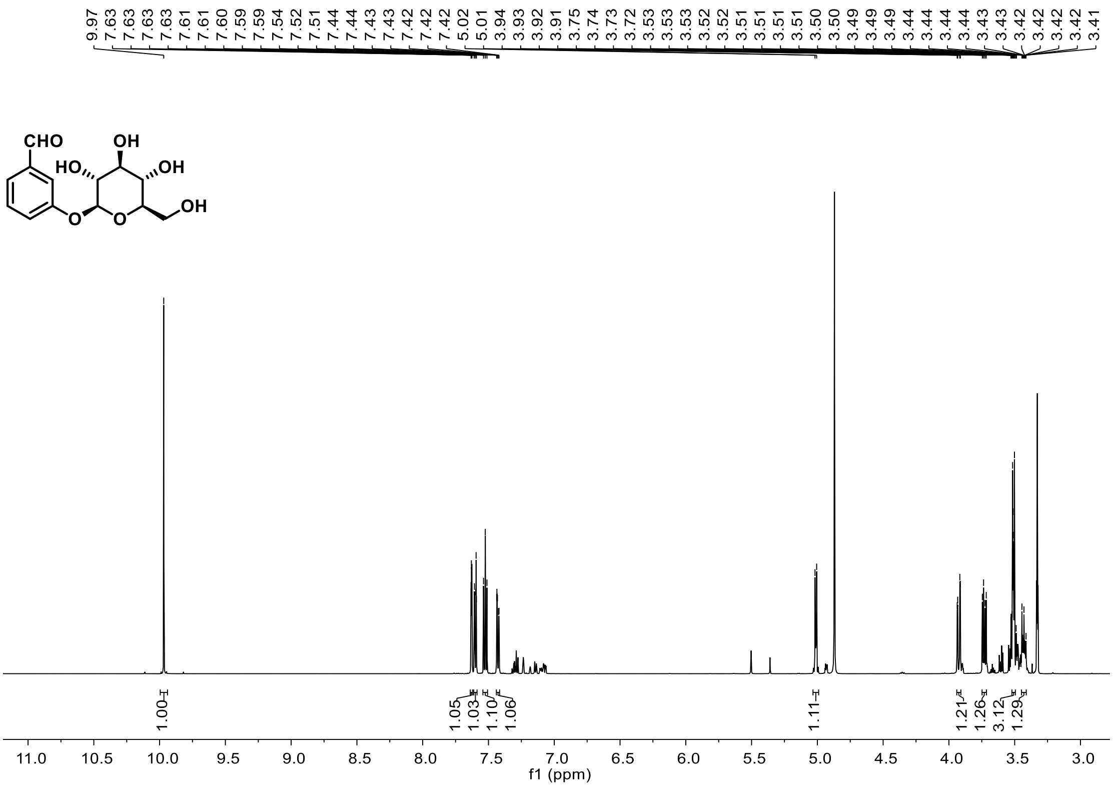

Fig. S51. ^{1}H NMR spectrum of 16a in CD3OD.

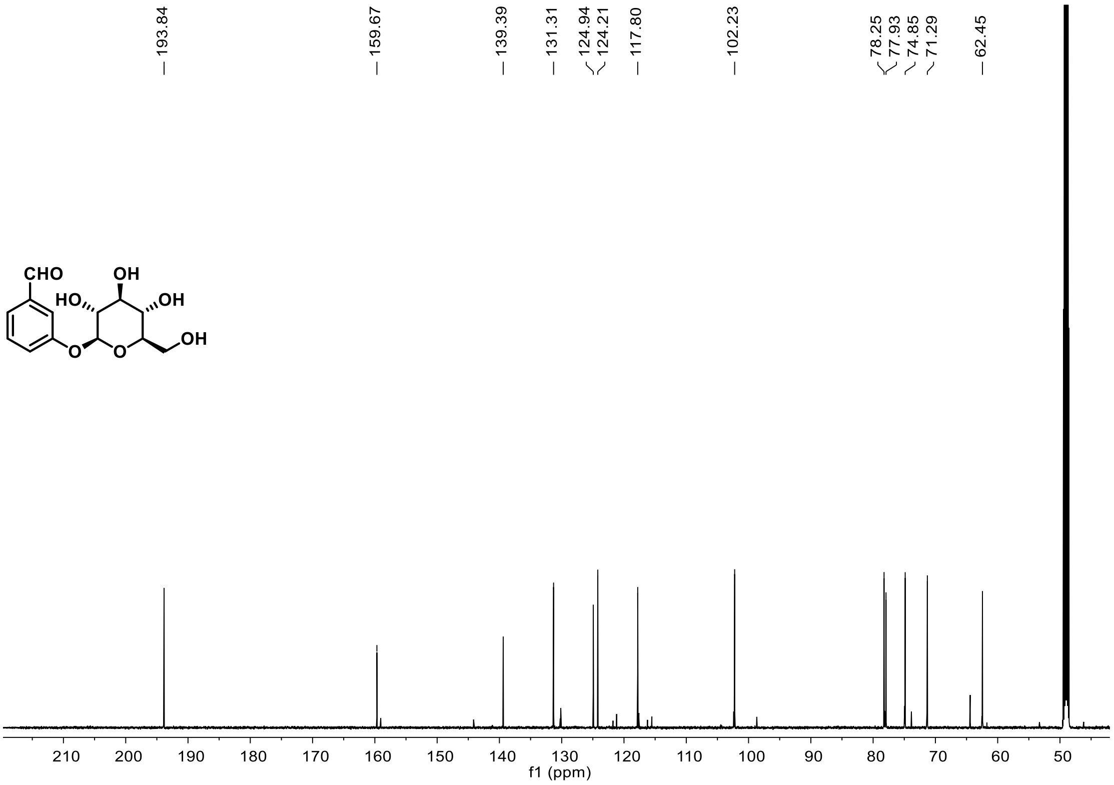

Fig. S52. ^{13}C NMR spectrum of 16a in CD3OD.

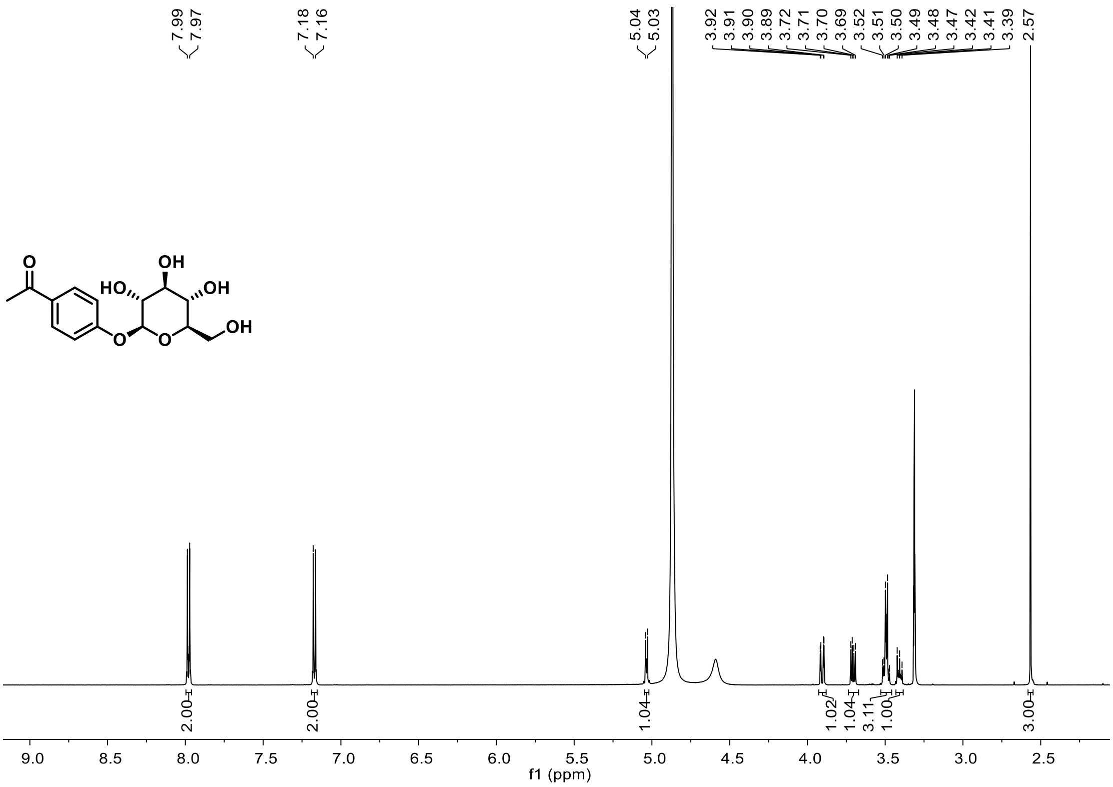

Fig. S53. ^{1}H NMR spectrum of 17a in CD$_3$OD.

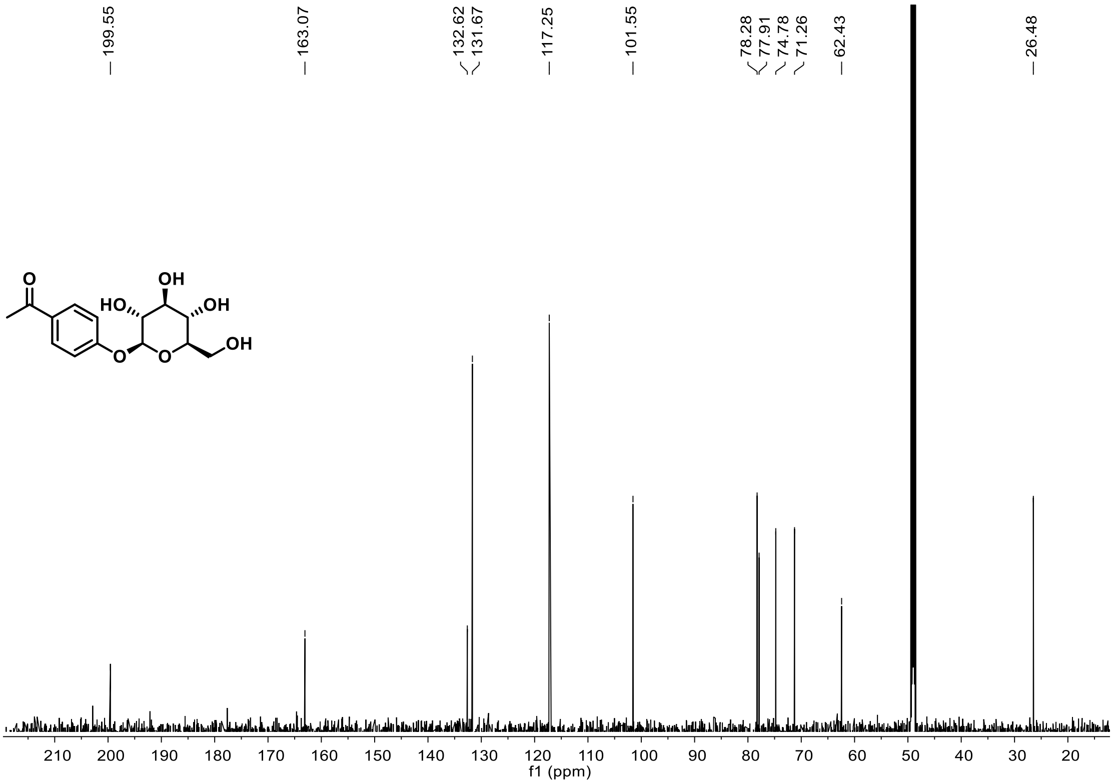

Fig. S54. ^{13}C NMR spectrum of 17a in CD$_3$OD.

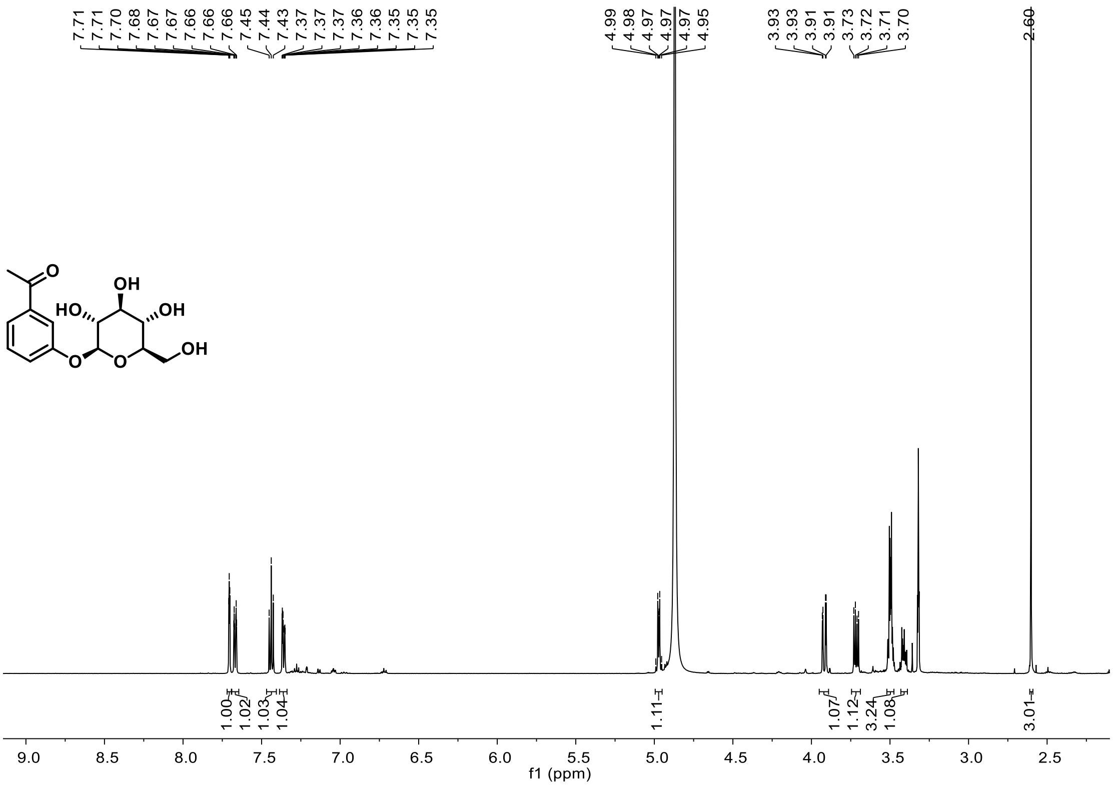

Fig. S55. ^{1}**H NMR spectrum of 18a in CD$_3$OD.**

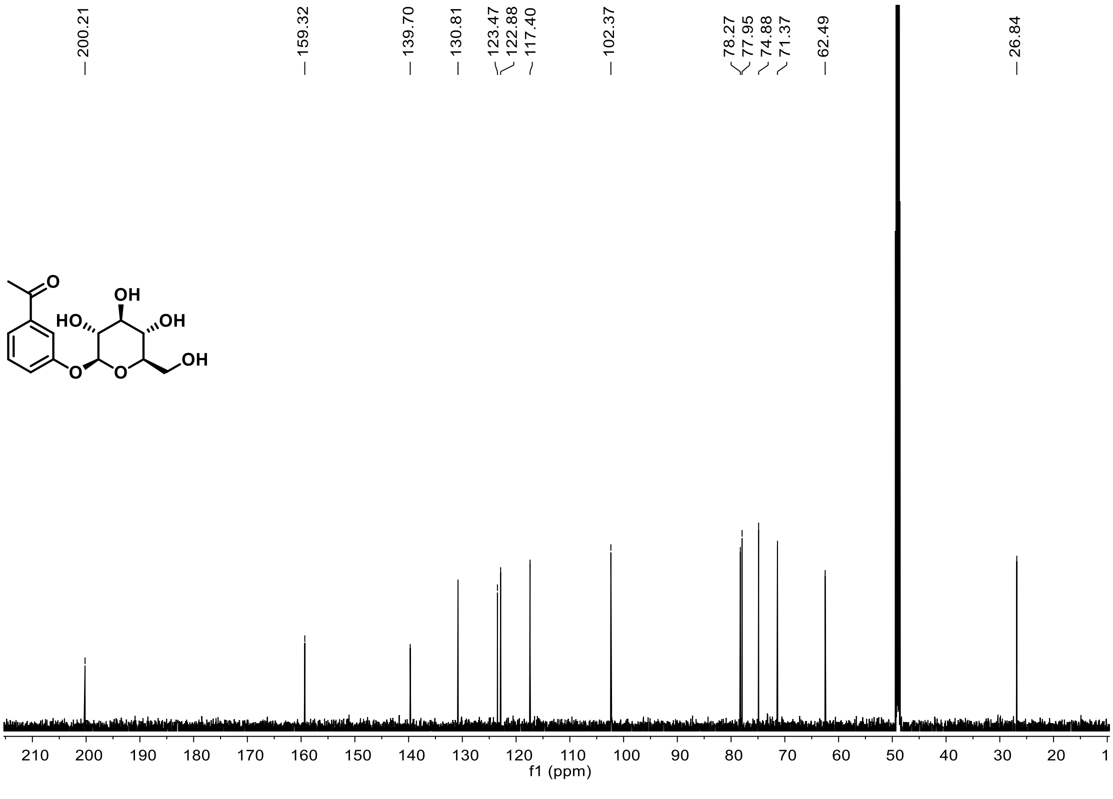

Fig. S56. ^{13}**C NMR spectrum of 18a in CD$_3$OD.**

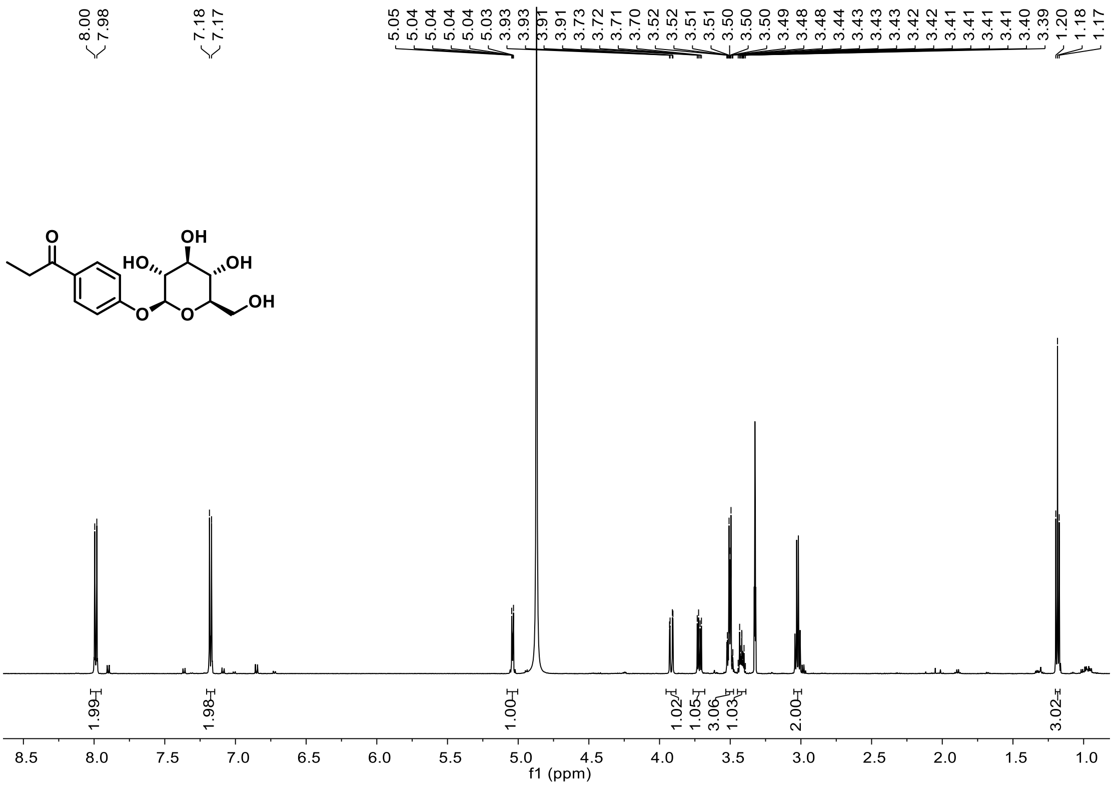

Fig. S57. ^{1}H NMR spectrum of 19a in CD_3OD.

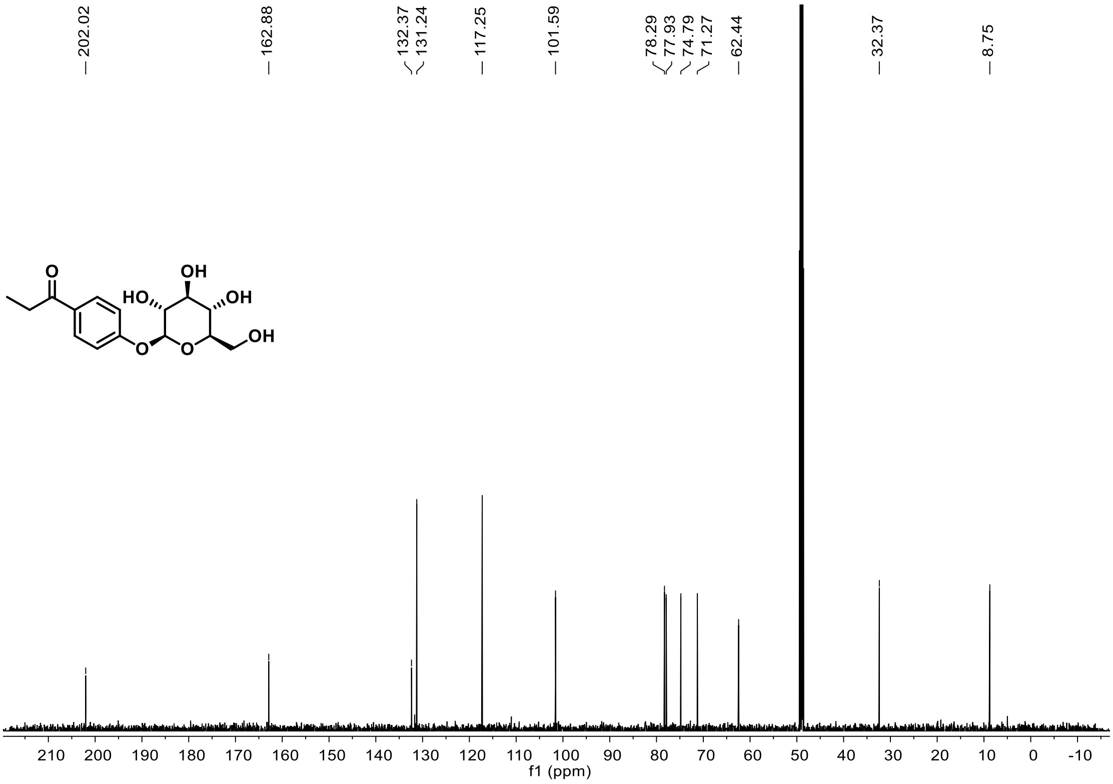

Fig. S58. ^{13}C NMR spectrum of 19a in CD_3OD.

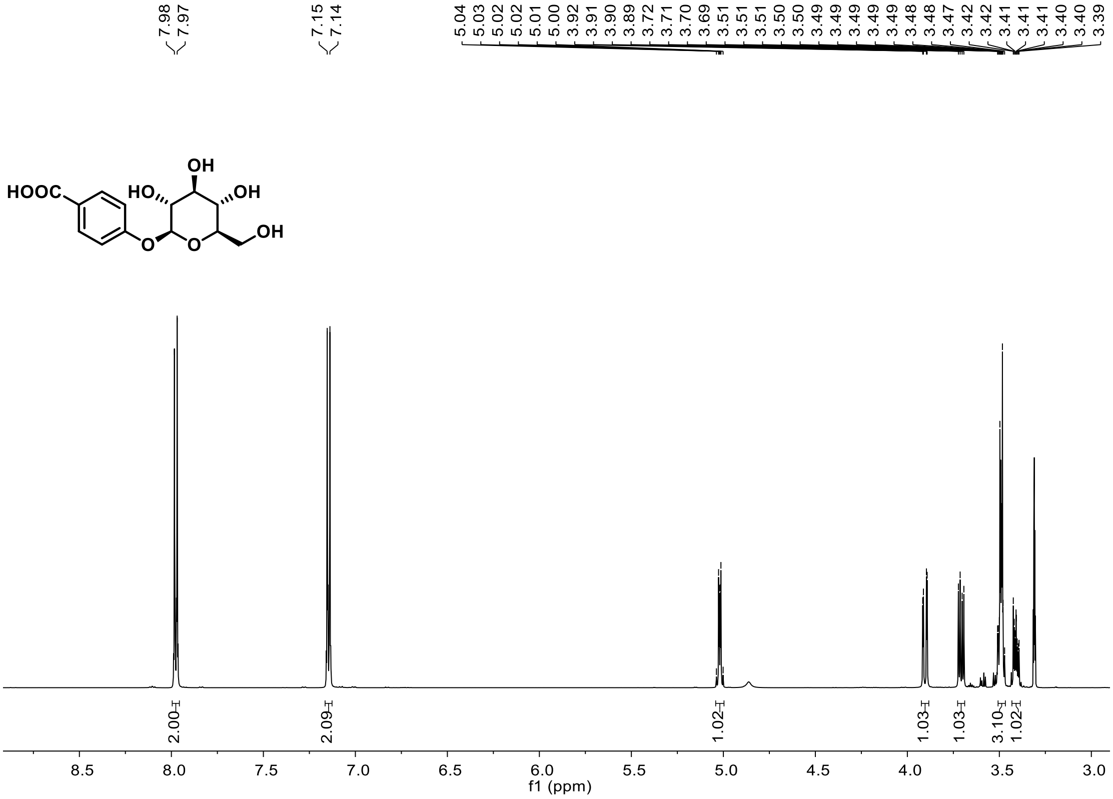

Fig. S59. ^{1}H NMR spectrum of 20a in CD3OD.

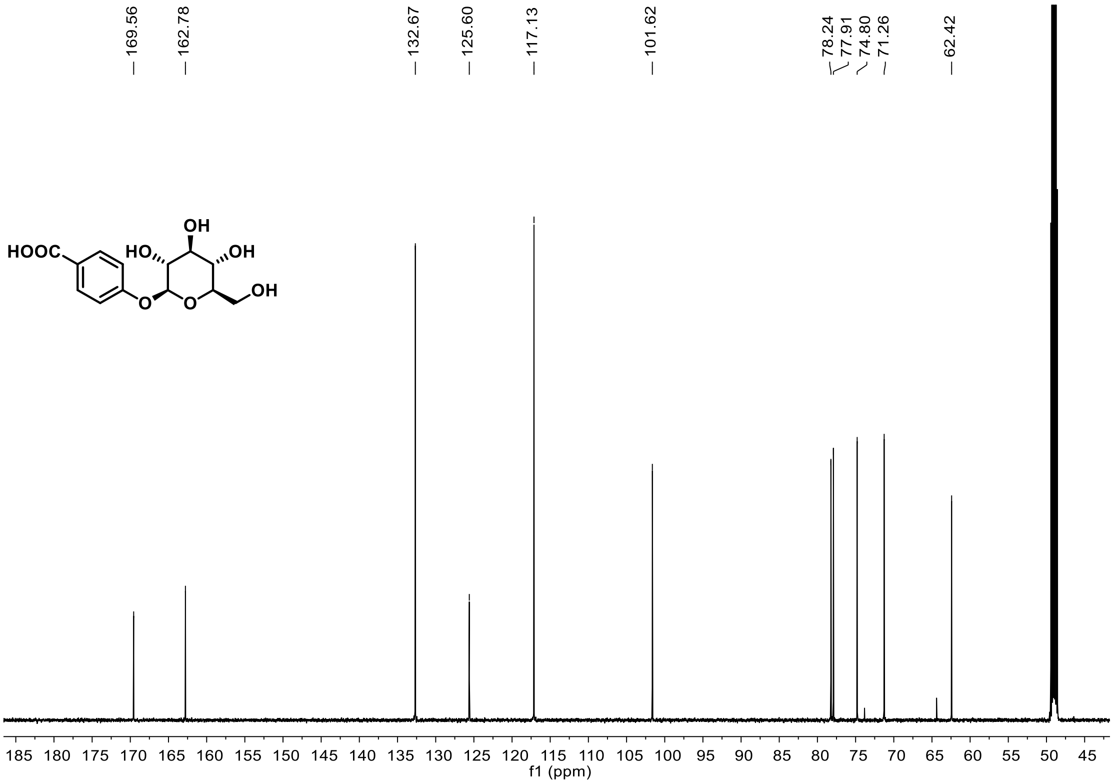

Fig. S60. ^{13}C NMR spectrum of 20a in CD3OD.

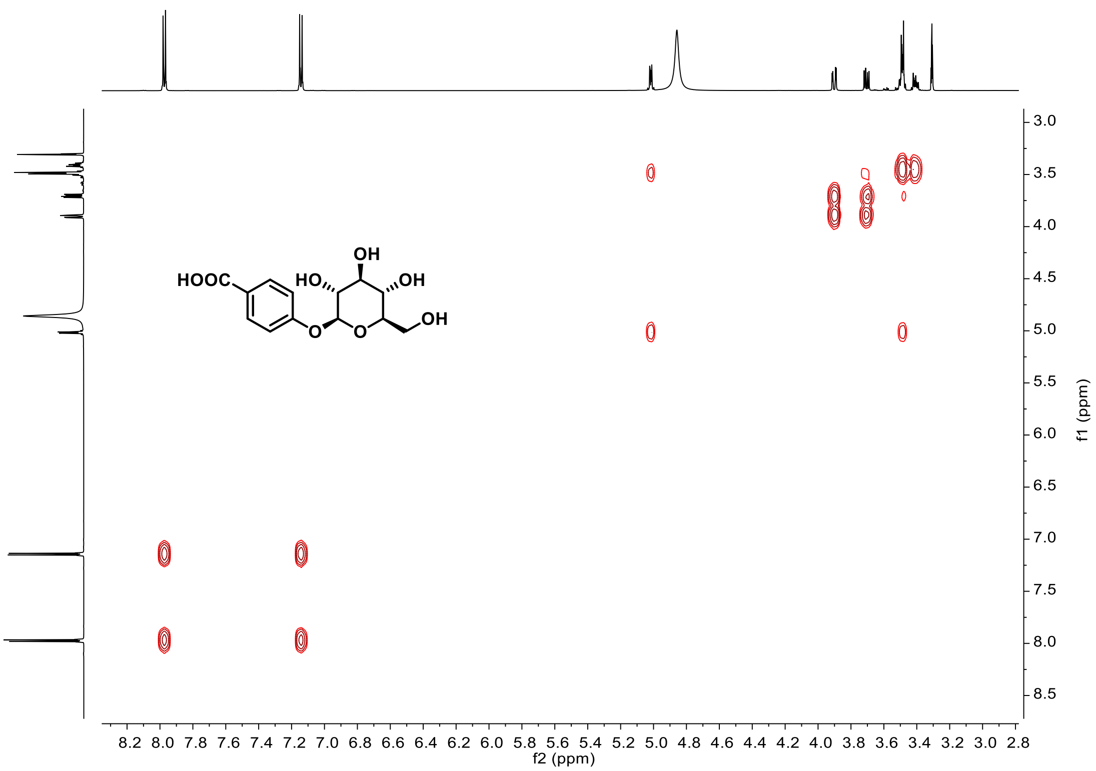

Fig. S61. COSY NMR spectrum of 20a in CD_3OD.

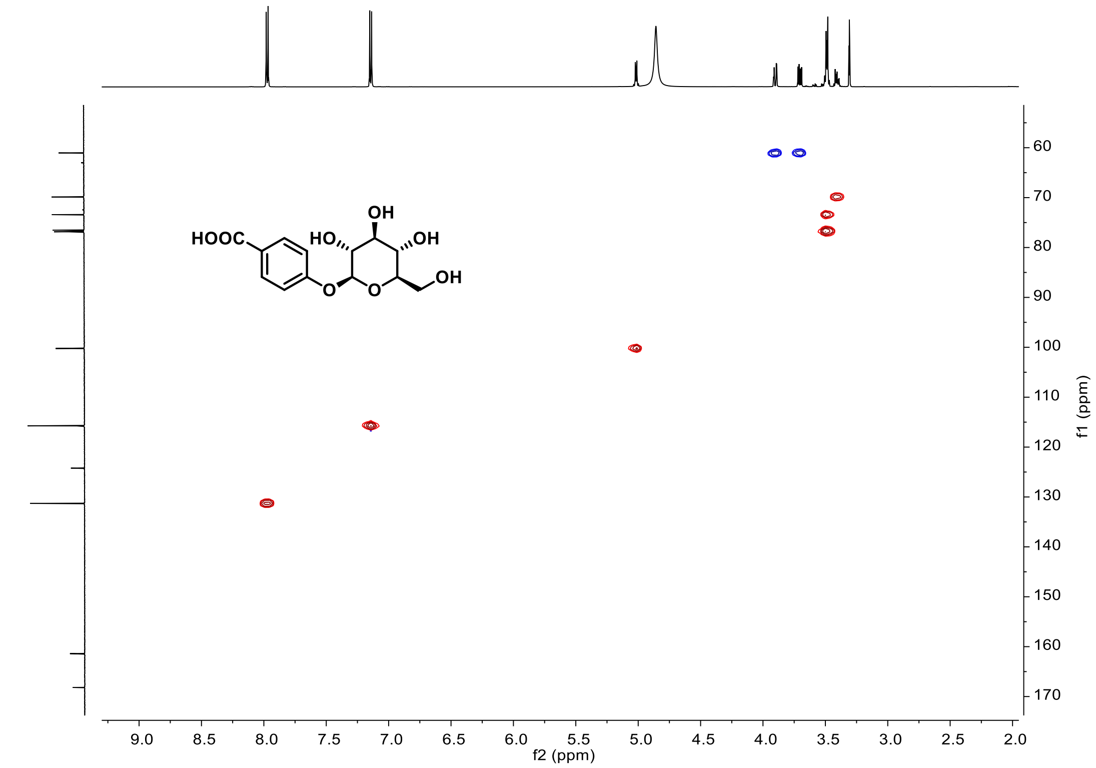

Fig. S62. HSQC NMR spectrum of 20a in CD_3OD.

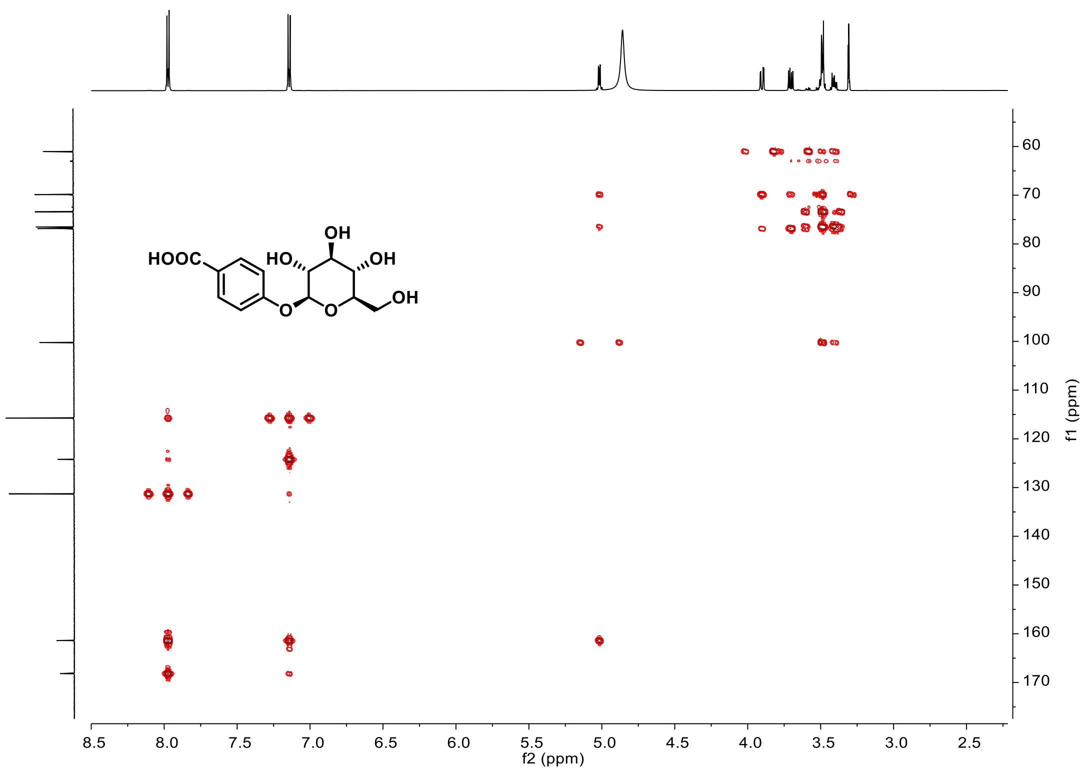

Fig. S63. HMBC NMR spectrum of 20a in CD3OD.

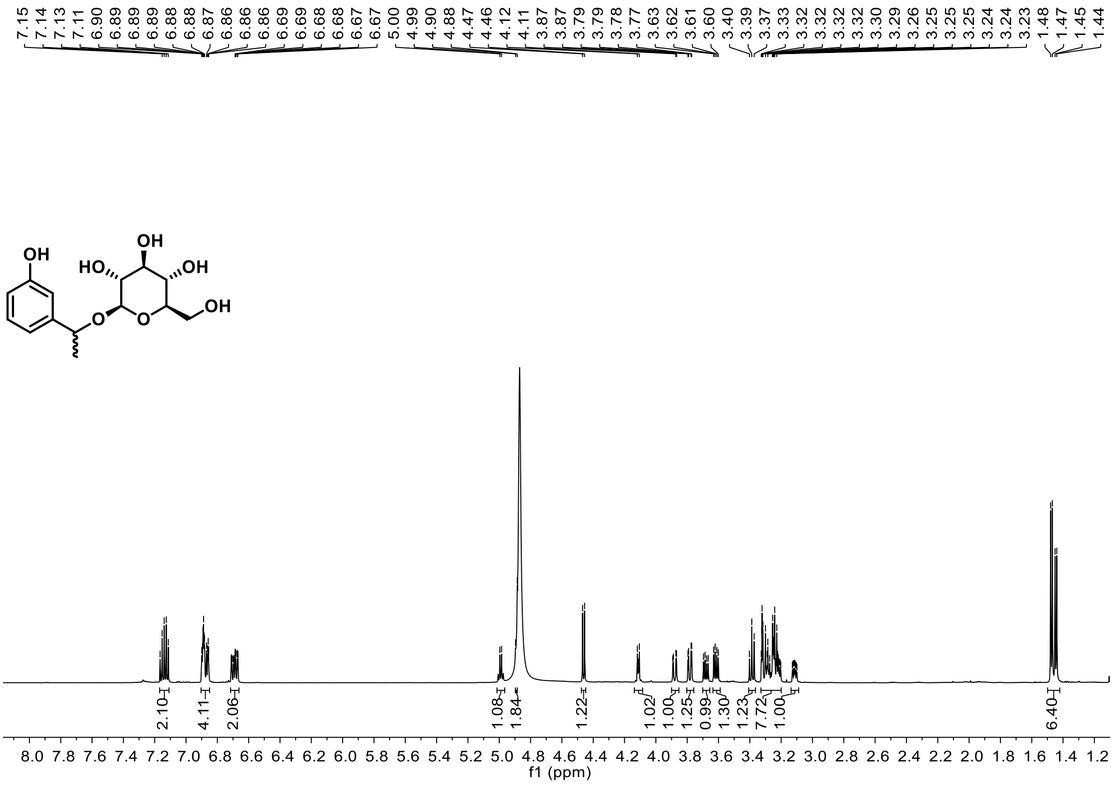

Fig. S64. ^{1}H NMR spectrum of 14b in CD3OD.

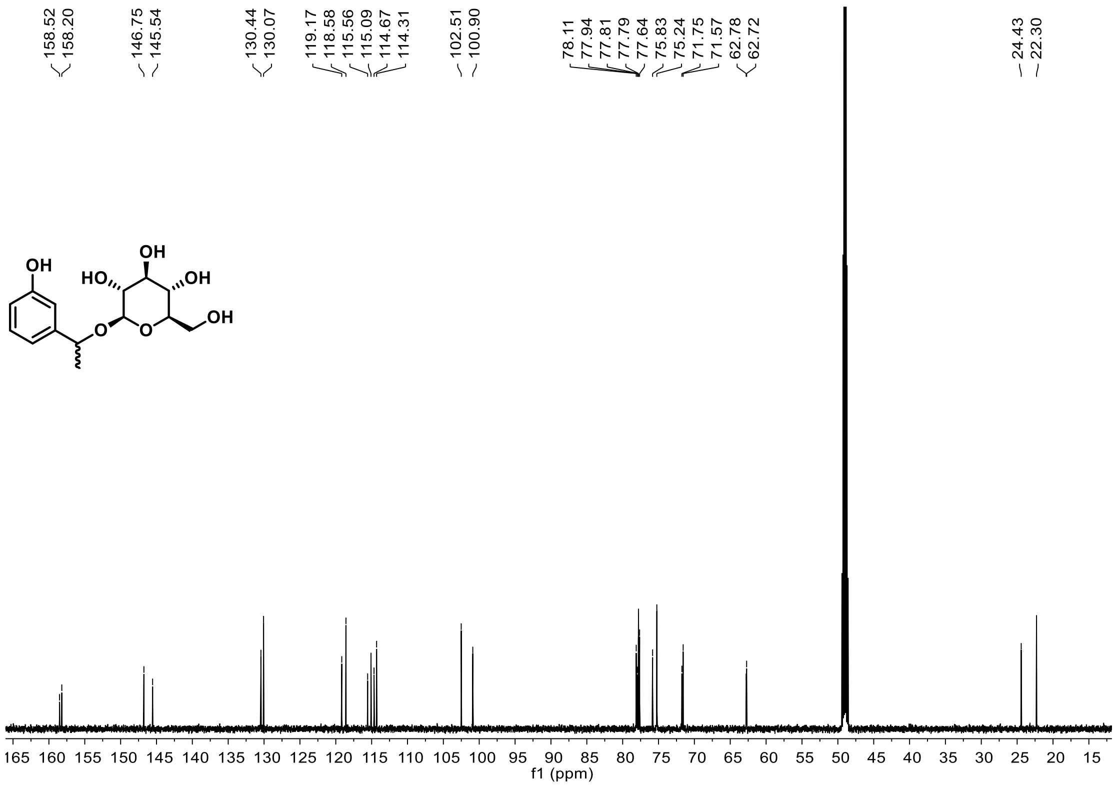

Fig. S65. ^{13}C NMR spectrum of 14b in CD3OD.

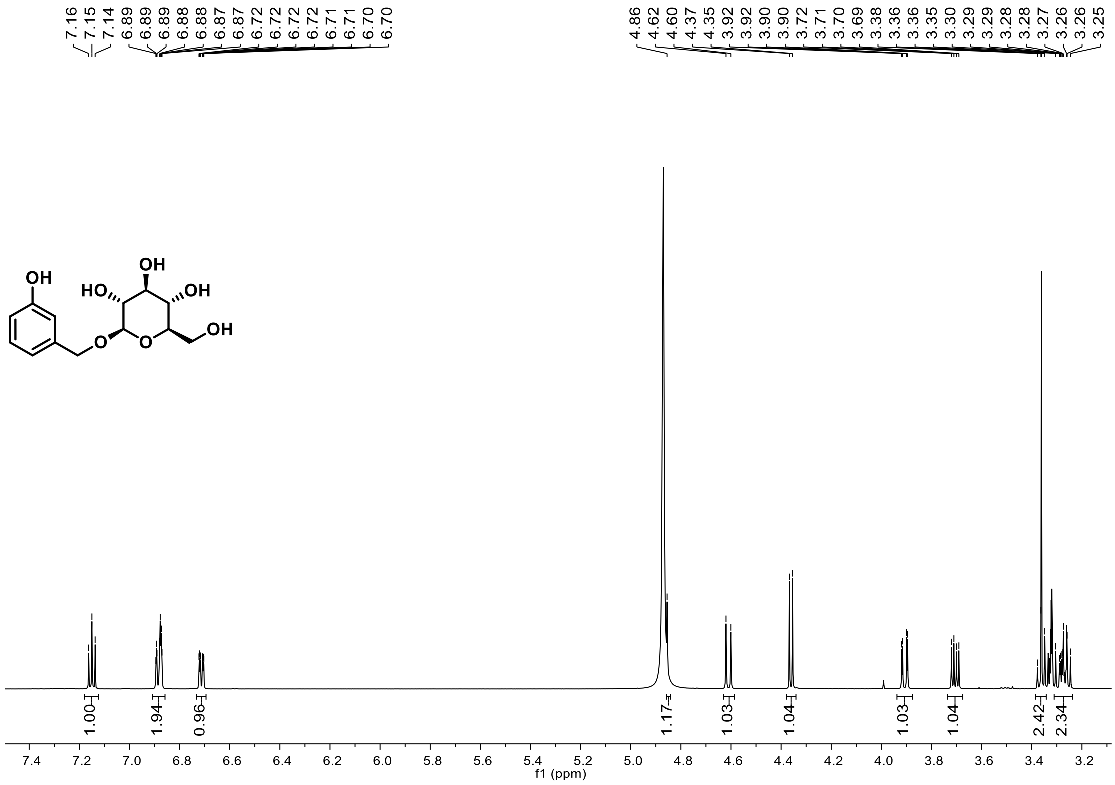

Fig. S66. ^{1}H NMR spectrum of 12b in CD3OD.

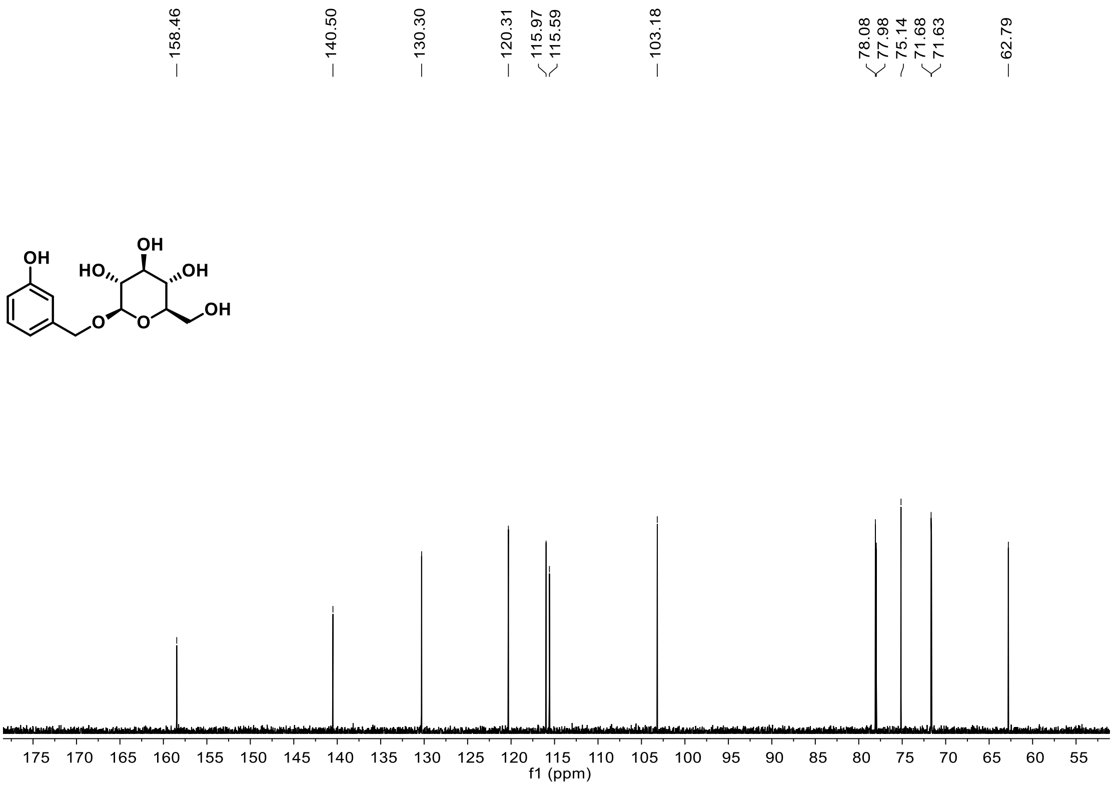

Fig. S67. ^{13}**C NMR spectrum of 12b in** CD_3OD**.**

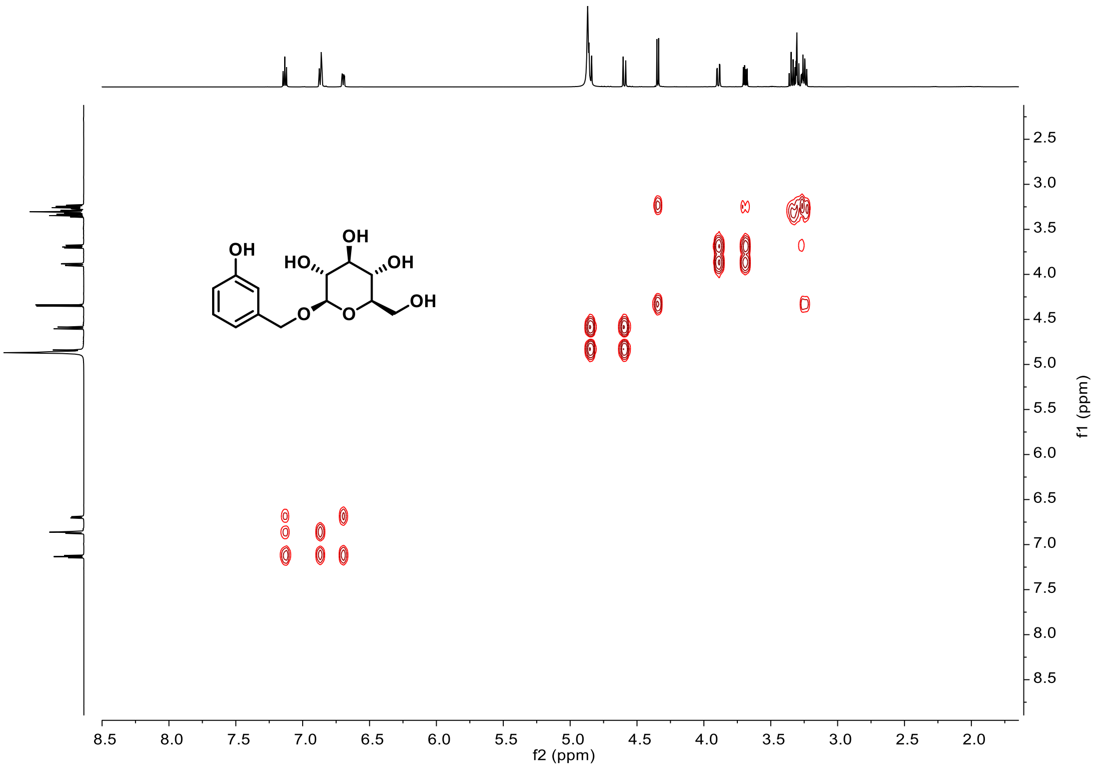

Fig. S68. COSY NMR spectrum of 12b in CD_3OD**.**

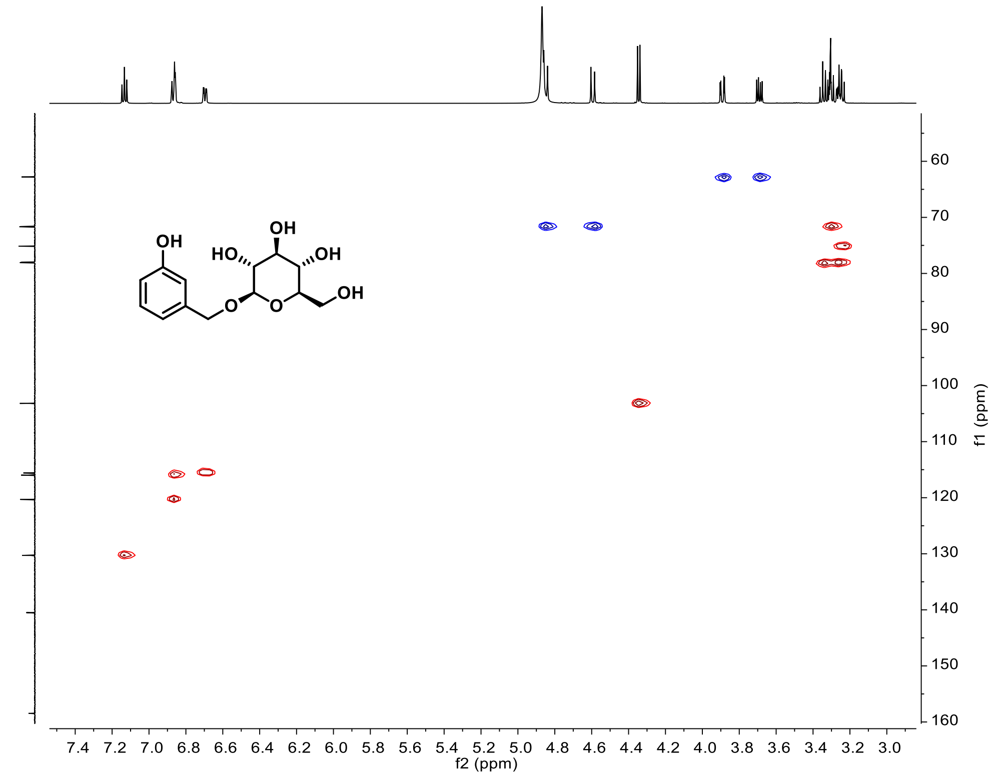

Fig. S69. HSQC NMR spectrum of 12b in CD_3OD.

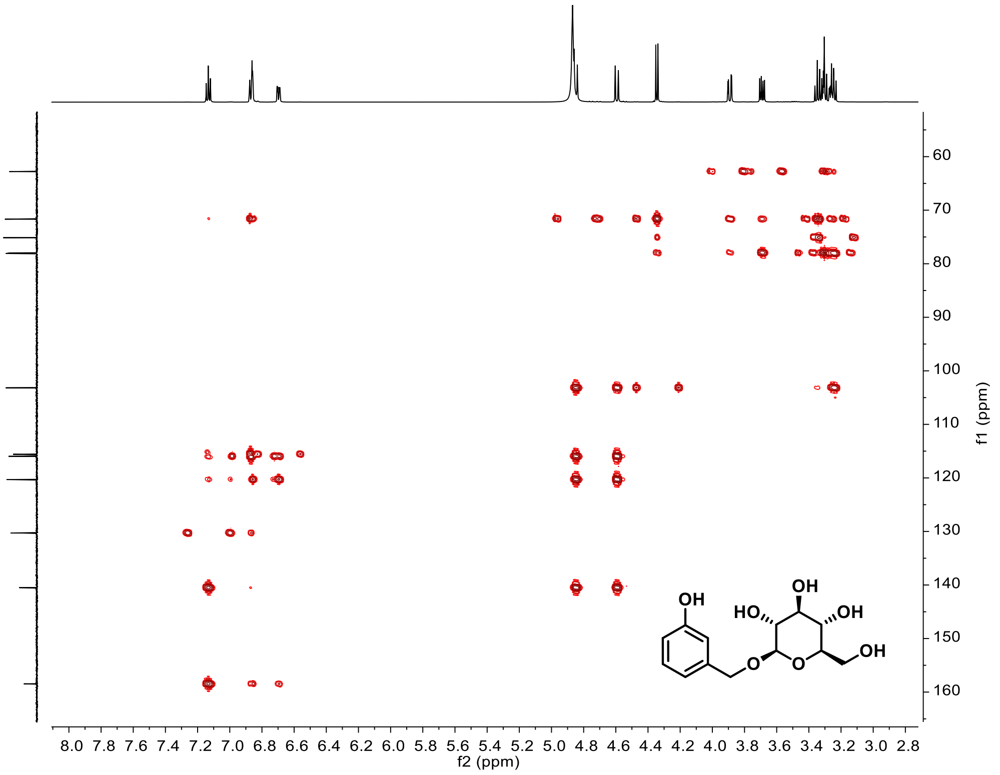

Fig. S70. HMBC NMR spectrum of 12b in CD_3OD.

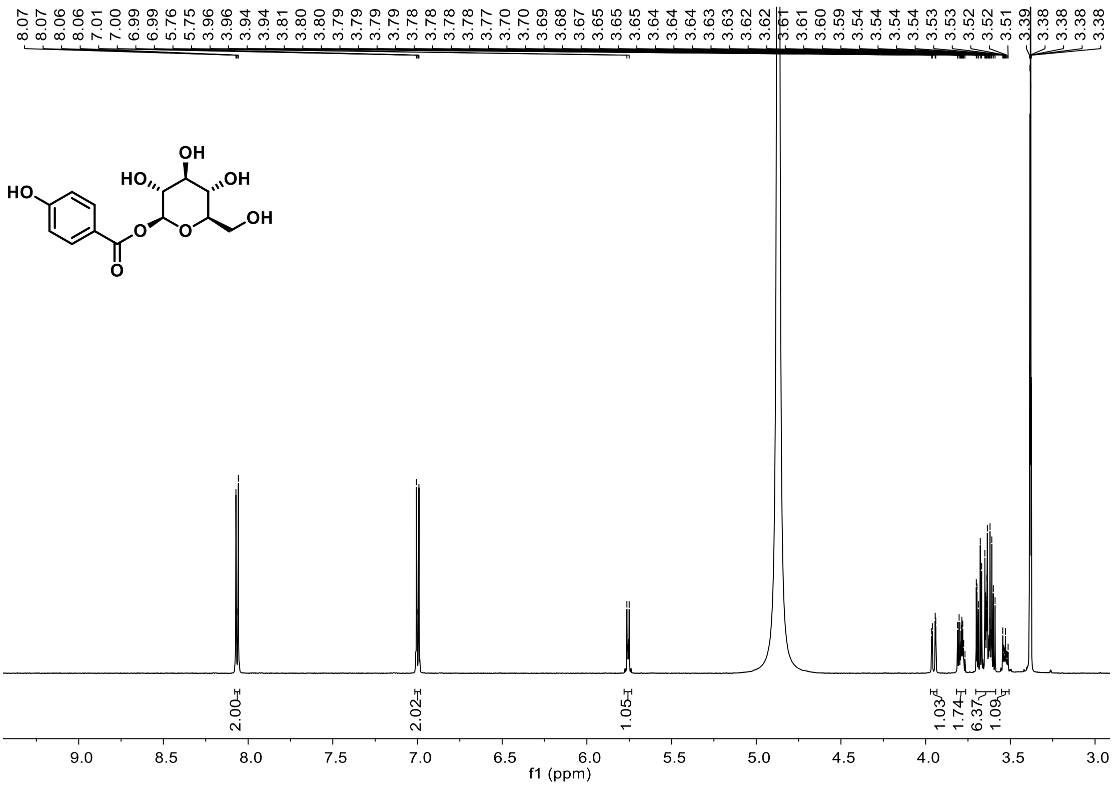

Fig. S71. ¹H NMR spectrum of 20b in CD₃OD. Note: the signals between the chemical shift of 3.5 and 4 is overlapped with the co-isolated residual glycerol.

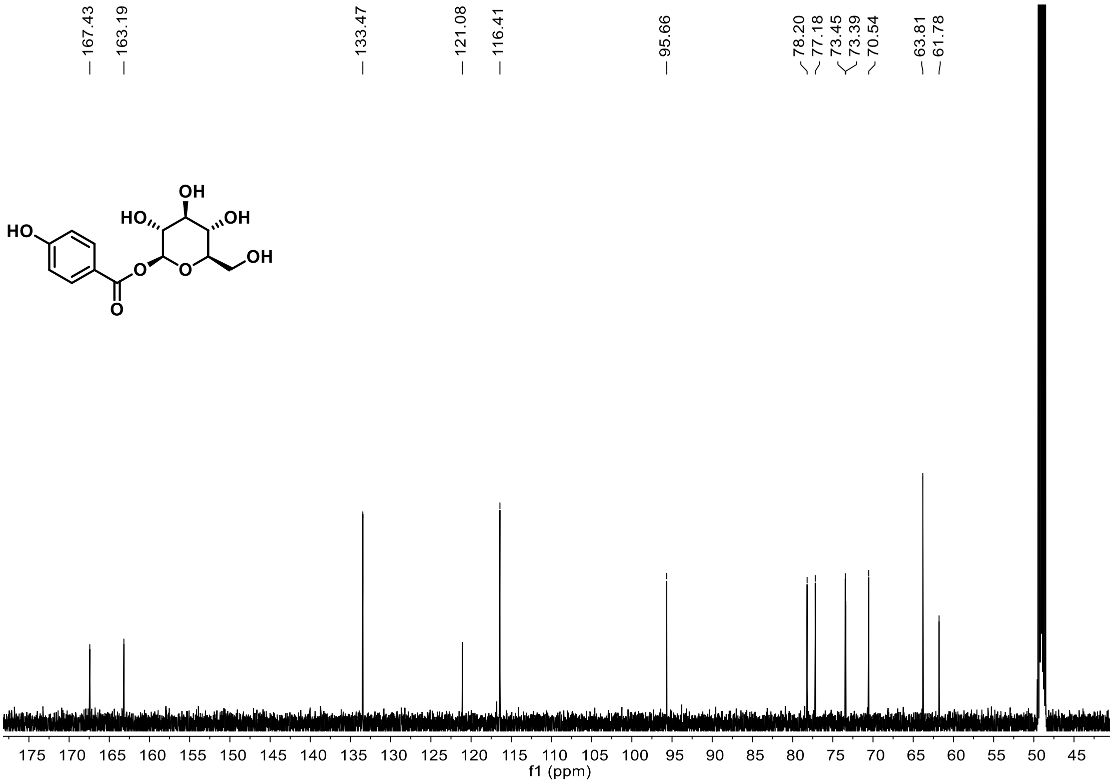

Fig. S72. ¹³C NMR spectrum of 20b in CD₃OD. Note: the signals of shifts 63.81 and 73.39 belong to the co-isolated residual glycerol.

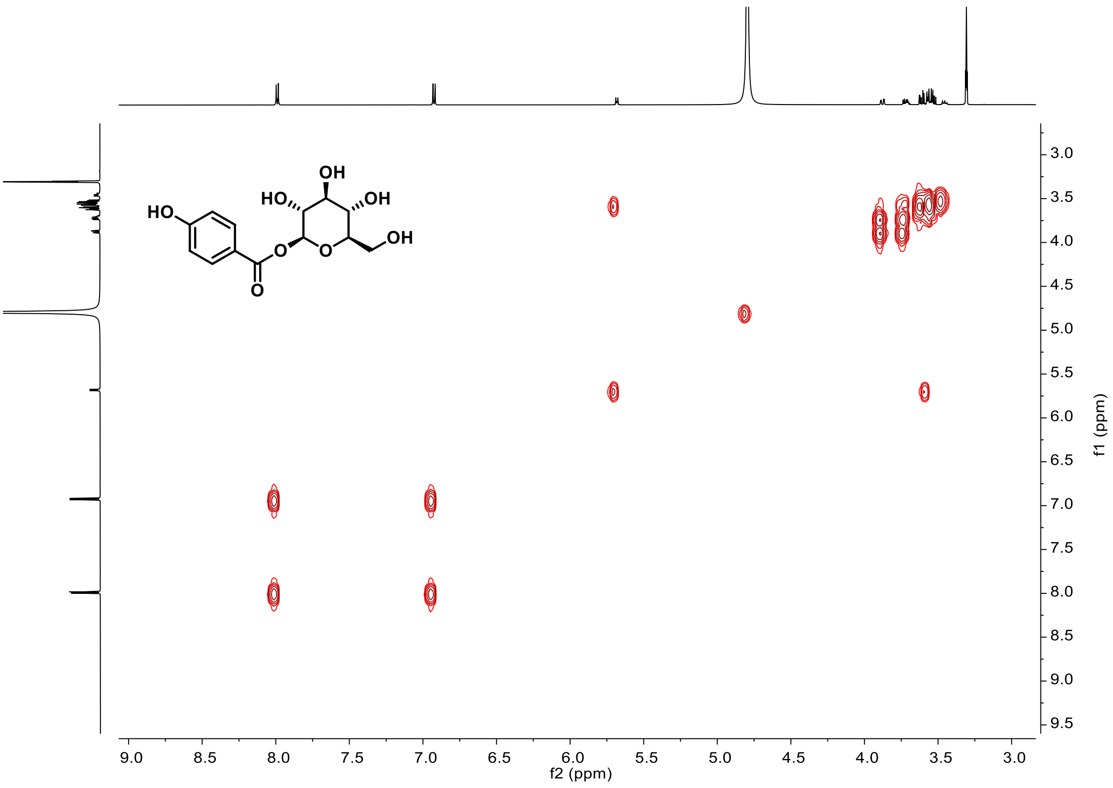

Fig. S73. COSY NMR spectrum of 20b in CD_3OD.

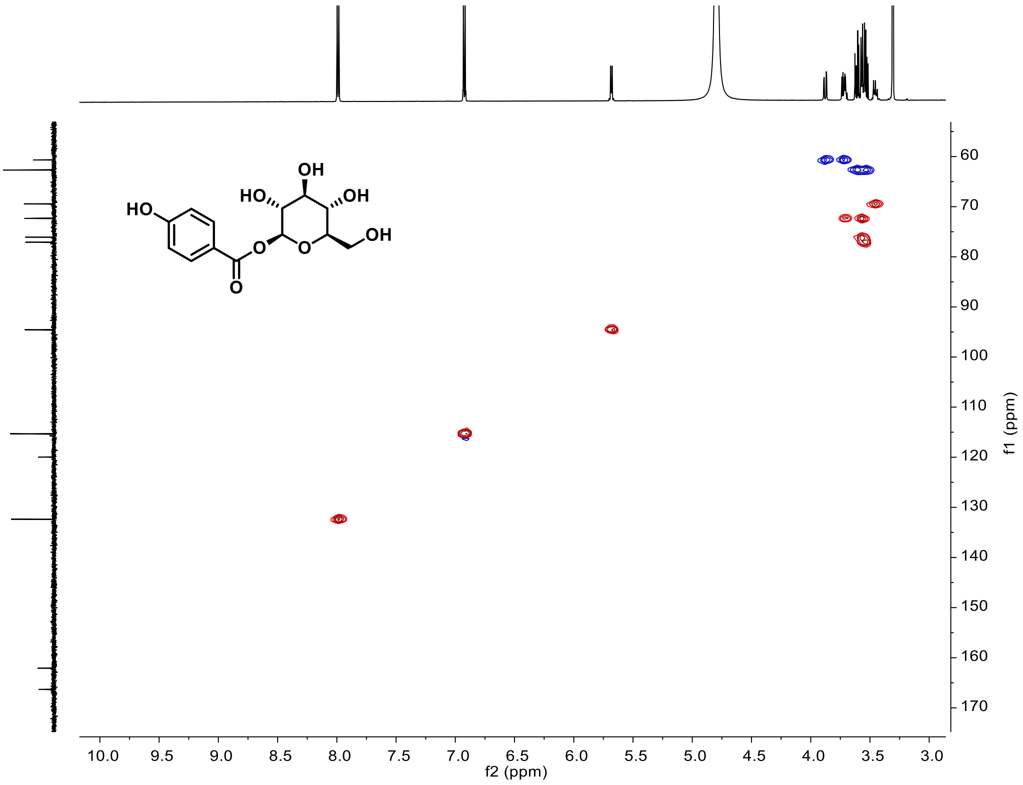

Fig. S74. HSQC NMR spectrum of 20b in CD_3OD.

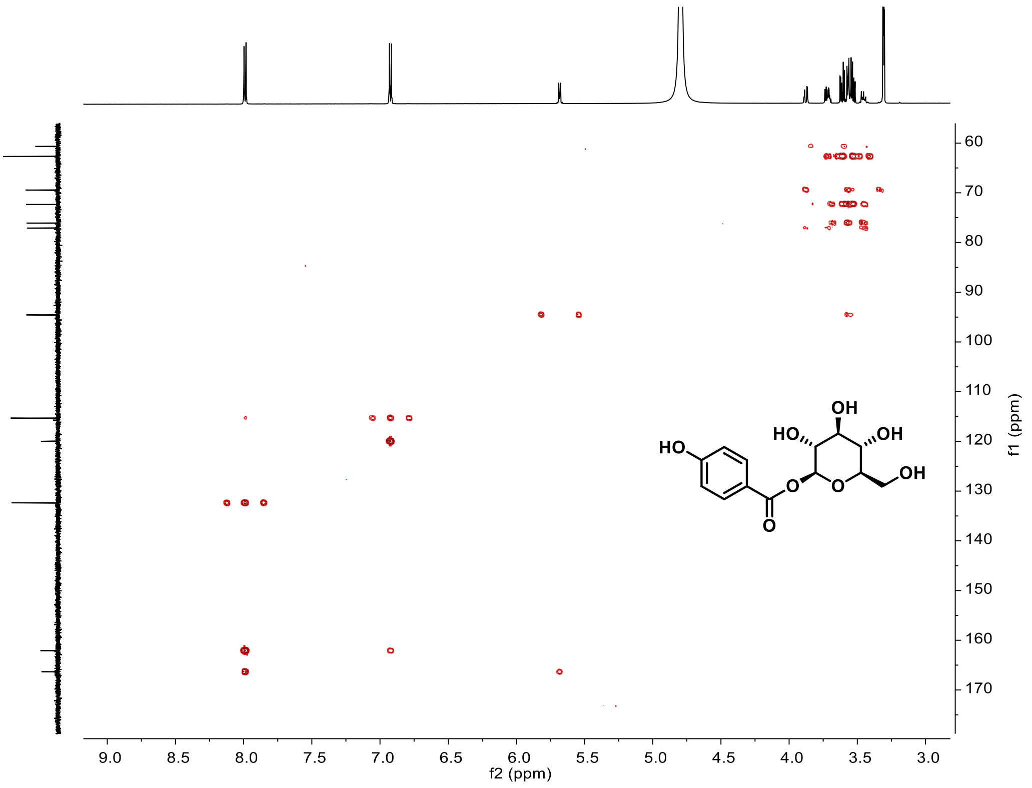

Fig. S75. HMBC NMR spectrum of 20b in CD_3OD.

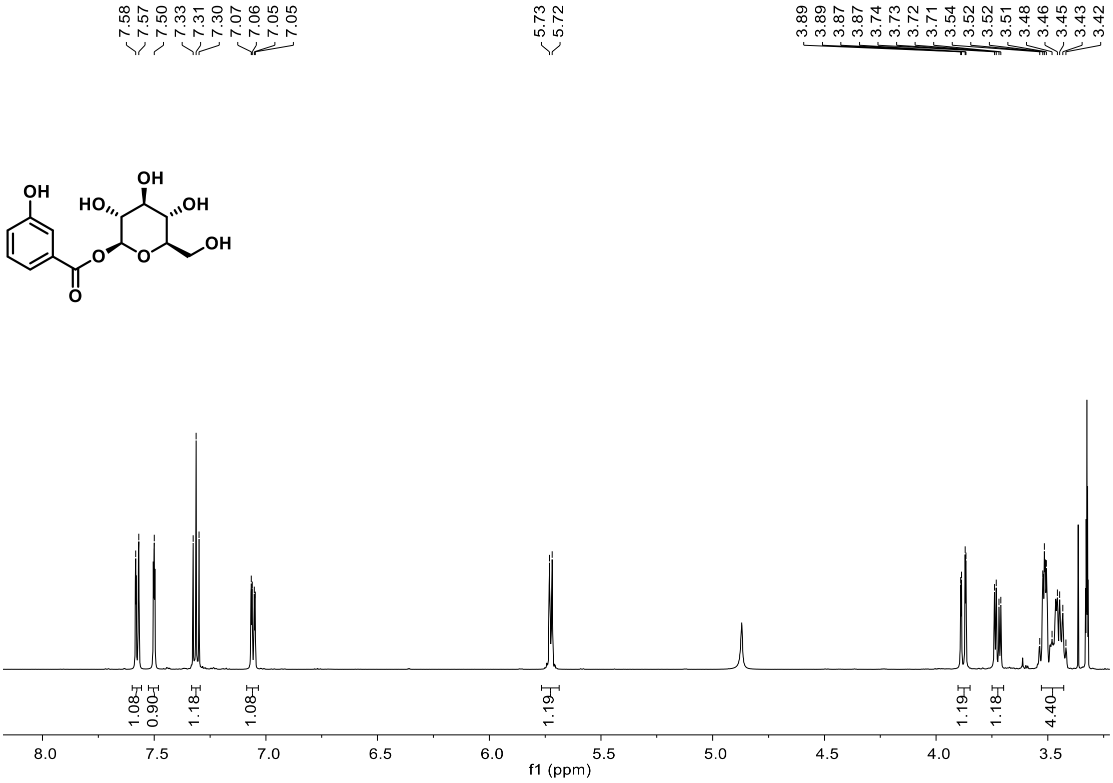

Fig. S76. ^{1}H NMR spectrum of 21b in CD_3OD.

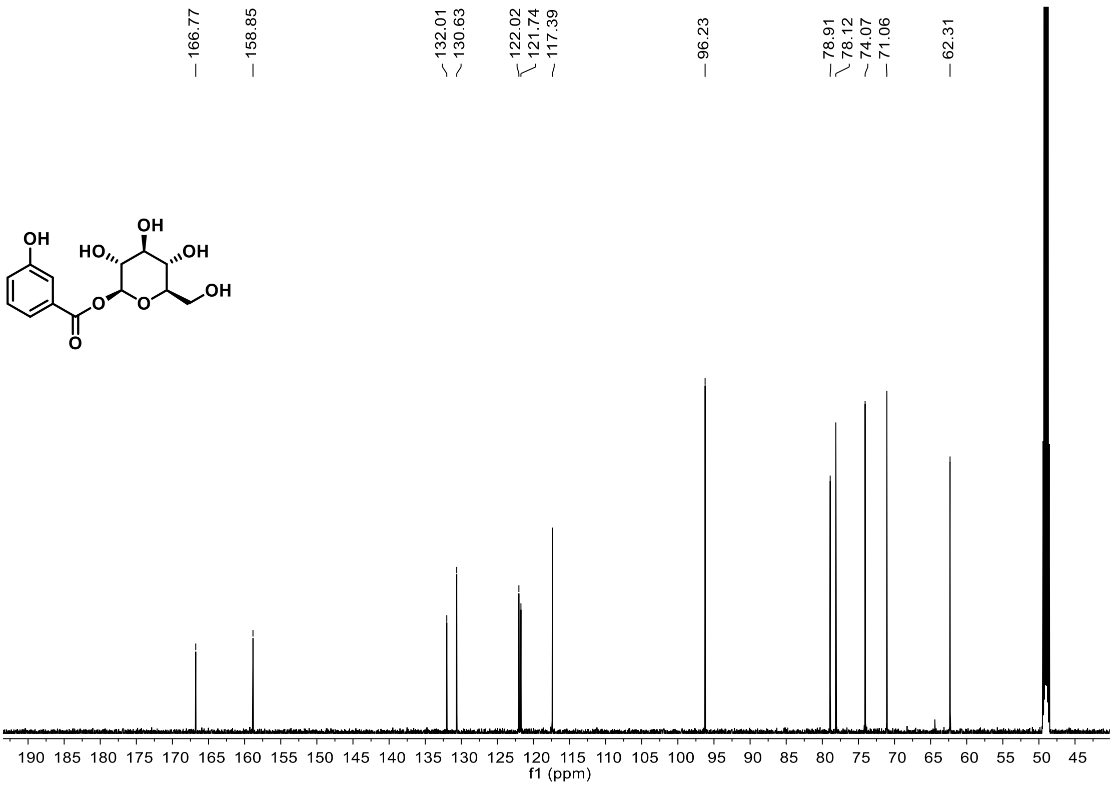

Fig. S77. ^{13}**C NMR spectrum of 21b in CD**$_3$**OD.**

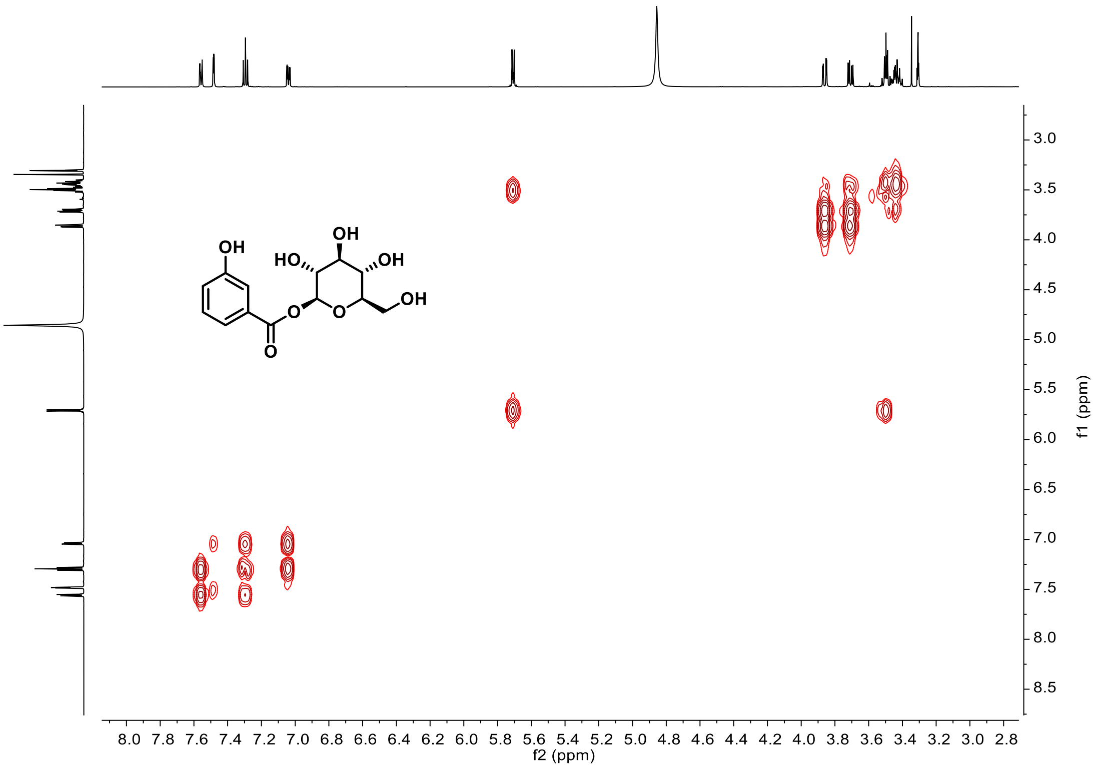

Fig. S78. COSY NMR spectrum of 21b in CD$_3$**OD.**

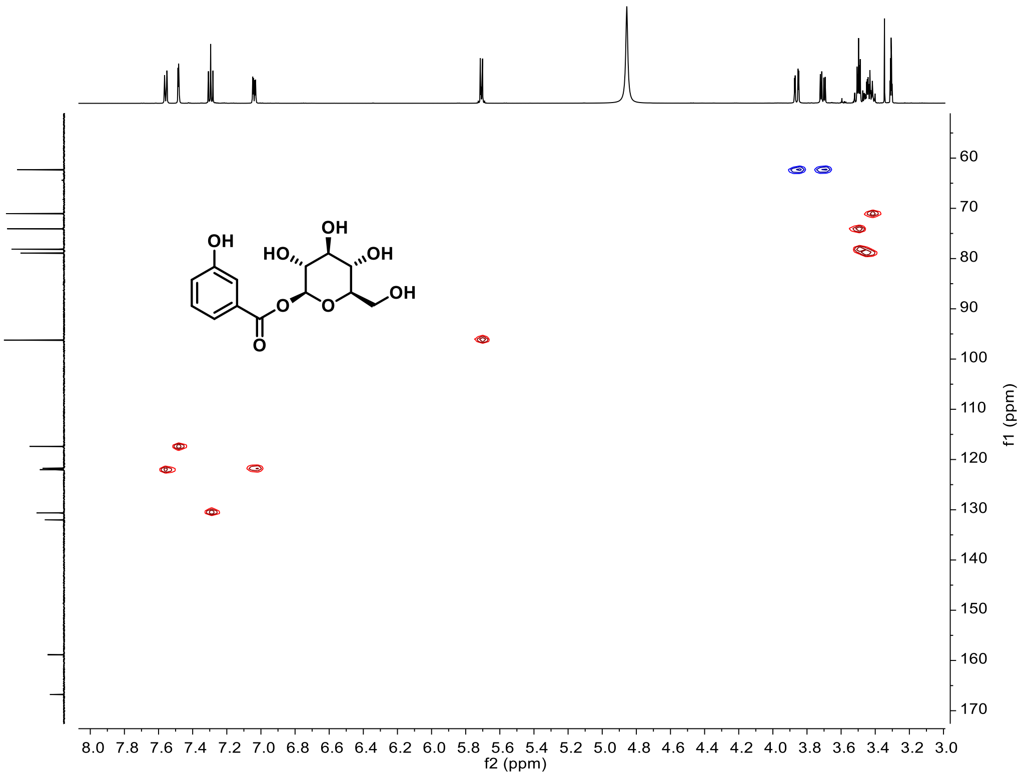

Fig. S79. HSQC NMR spectrum of 21b in CD3OD.

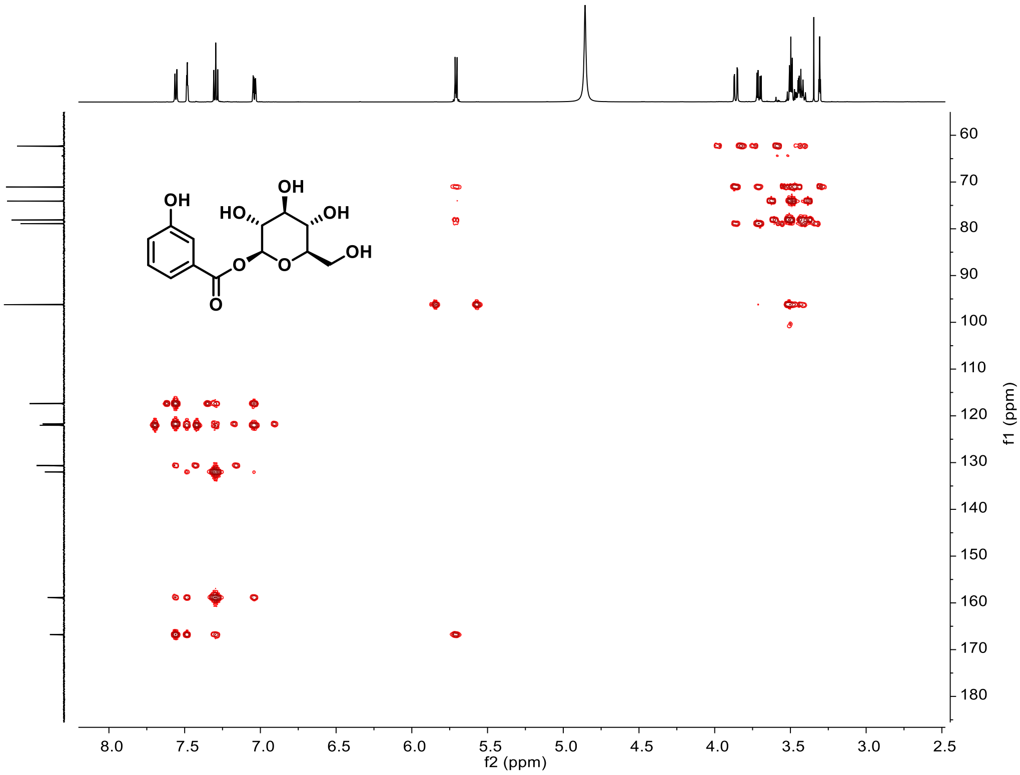

Fig. S80. HMBC NMR spectrum of 21b in CD3OD.

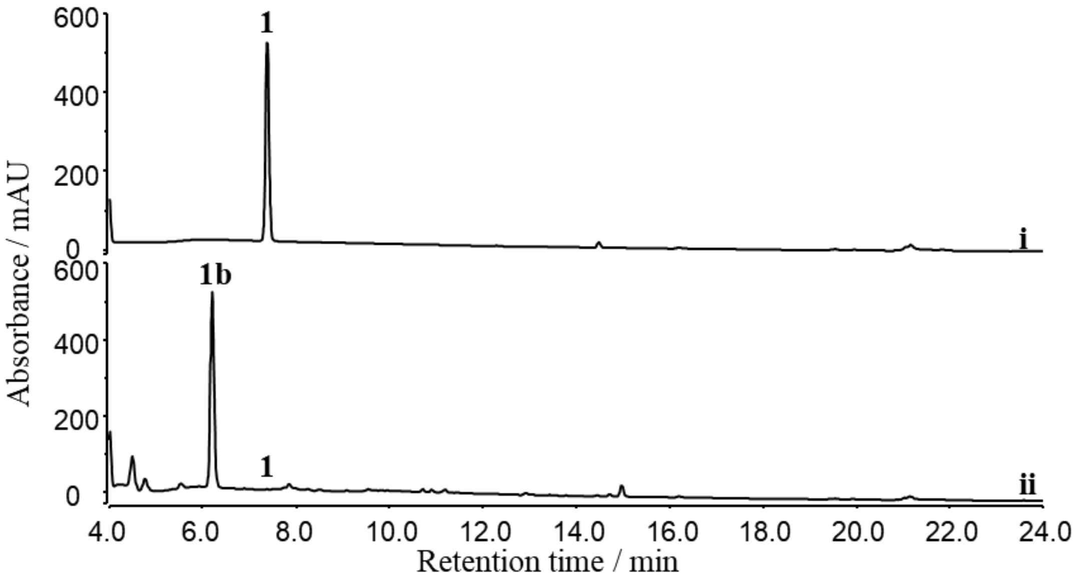

Fig. S81. Transformation of substrate 1 by *E. coli* BL21(DE3) containing UGT85A1. i, standard substrate **1**; ii, biotransformation. The conversion efficiency is 99.77%.

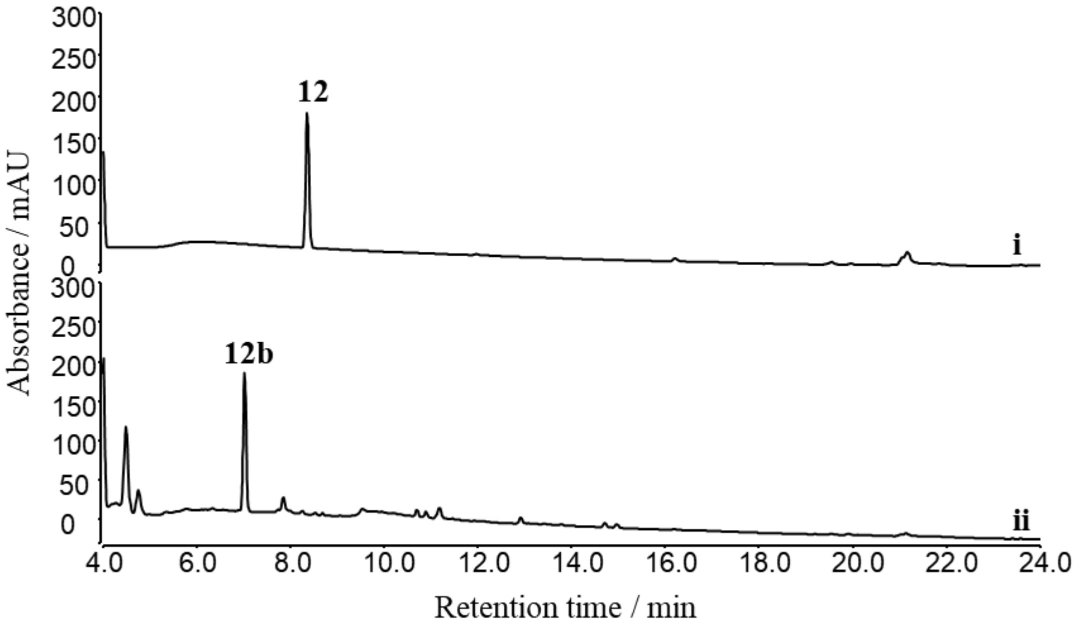

Fig. S82. Transformation of substrate 12 by *E. coli* BL21(DE3) containing UGT85A1. i, standard substrate **12**; ii, biotransformation. The conversion efficiency is 100%.

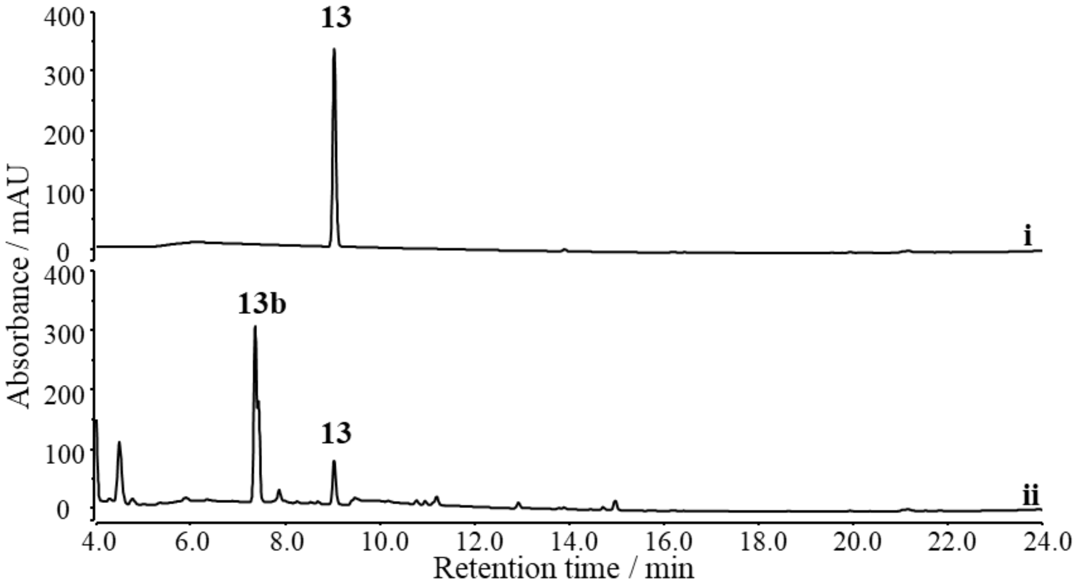

Fig. S83. Transformation of substrate 13 by *E. coli* BL21(DE3) containing UGT85A1. i, standard substrate **13**; ii, biotransformation. The conversion efficiency is 81.36%.

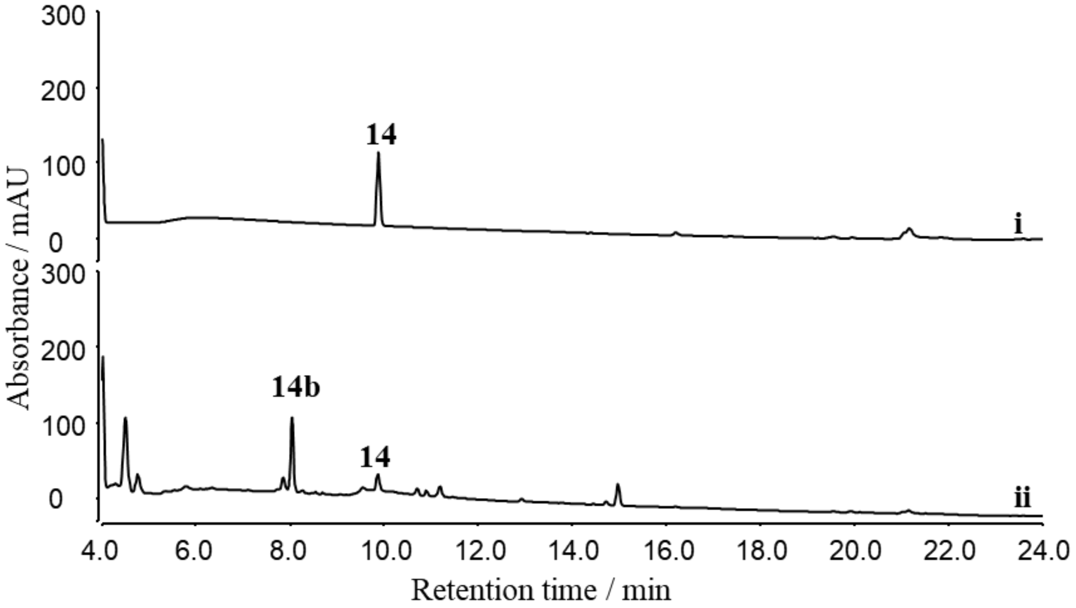

Fig. S84. Transformation of substrate 14 by *E. coli* BL21(DE3) containing UGT85A1. i, standard substrate **14**; ii, biotransformation. The conversion efficiency is 71.79%.

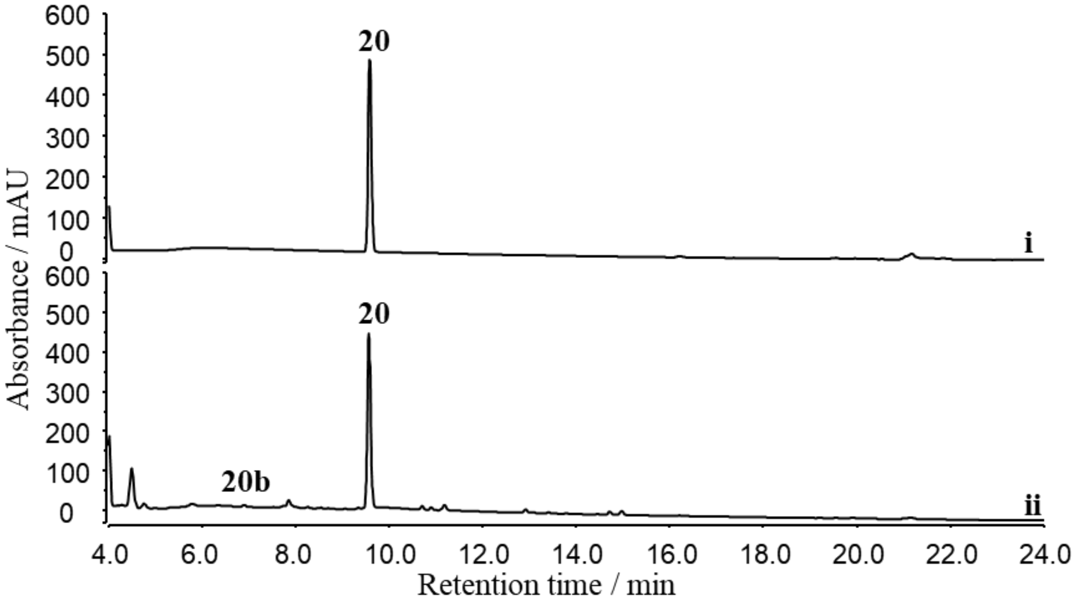

Fig. S85. Transformation of substrate 20 by *E. coli* BL21(DE3) containing UGT85A1. i, standard substrate **20**; ii, biotransformation. The conversion efficiency is 1.67%.

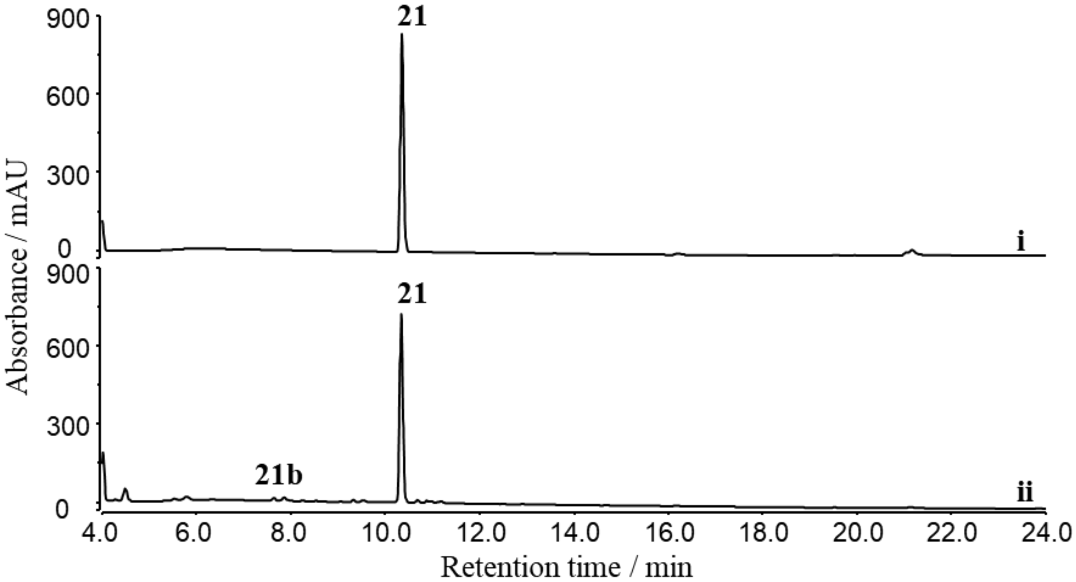

Fig. S86. Transformation of substrate 21 by *E. coli* BL21(DE3) containing UGT85A1. i, standard substrate **21**; ii, biotransformation. The conversion efficiency is 3.27%.

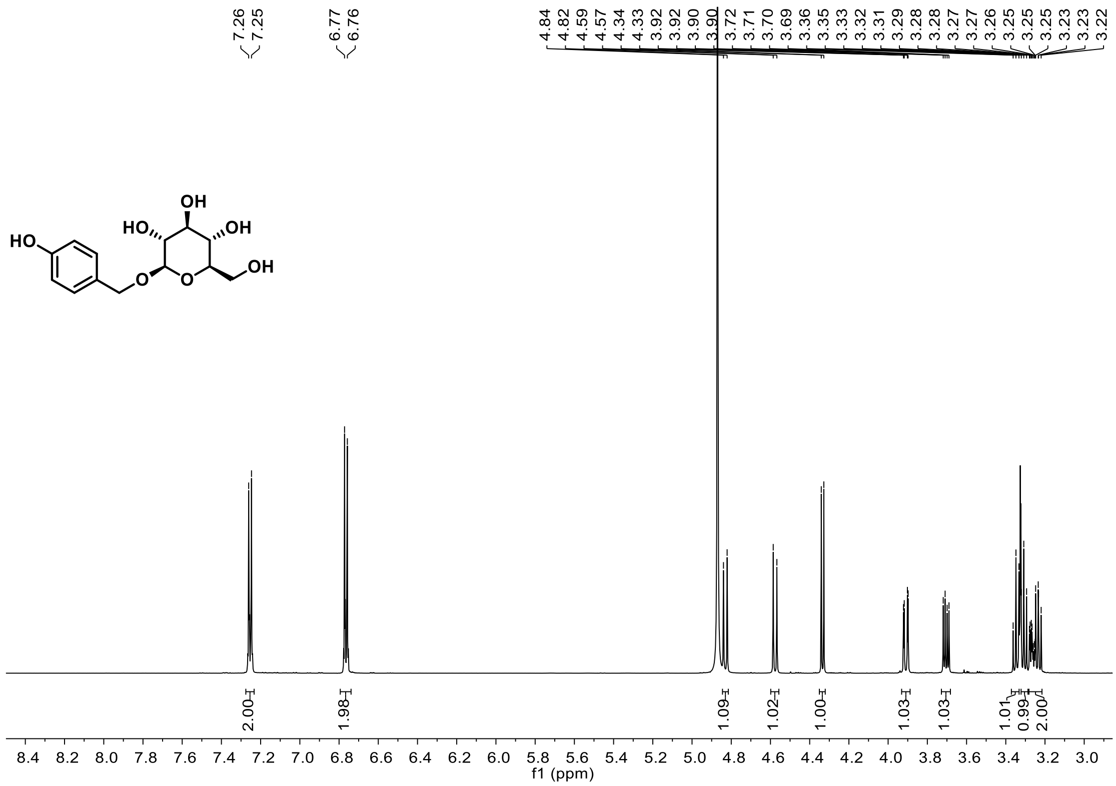

Fig. S87. ^{1}H NMR spectrum of 1b in CD_3OD.

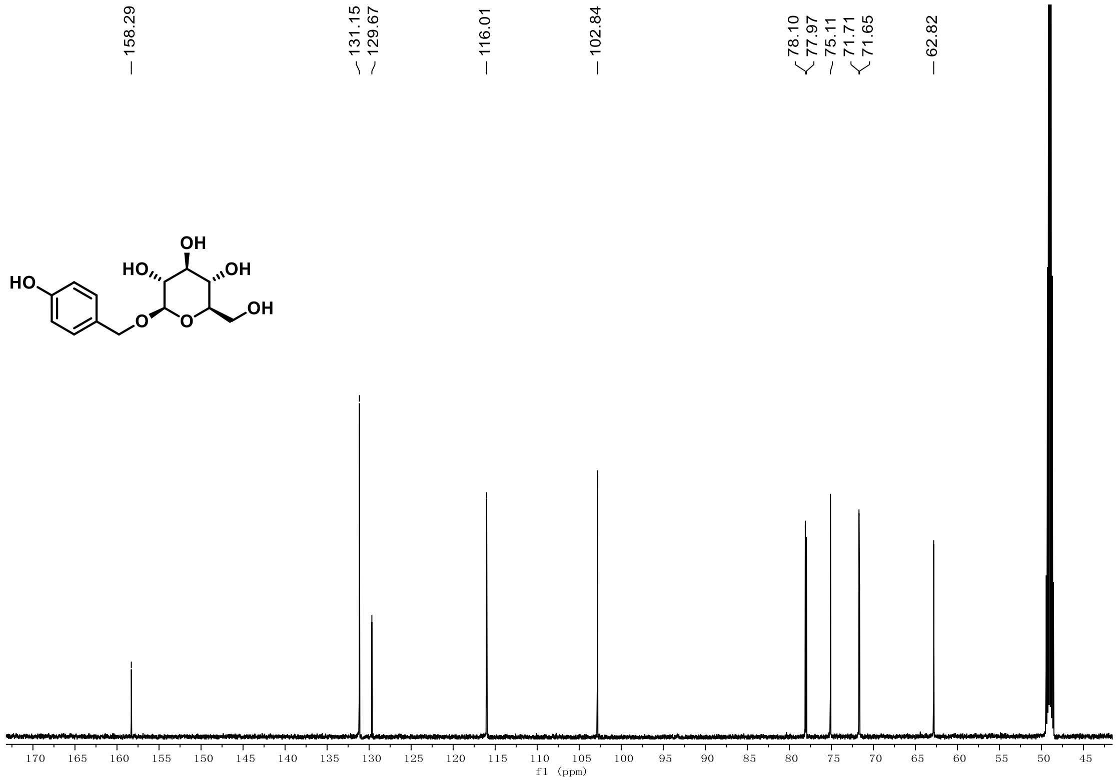

Fig. S88. ^{13}C NMR spectrum of 1b in CD_3OD.

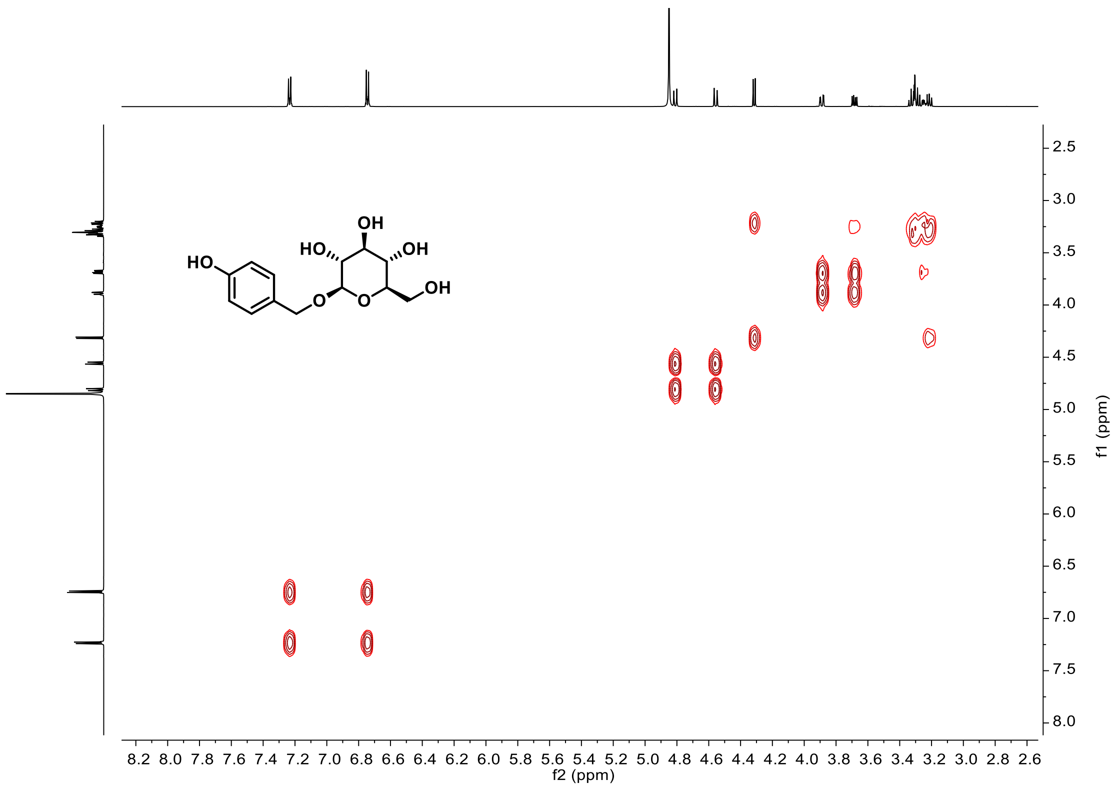

Fig. S89. COSY NMR spectrum of 1b in CD_3OD.

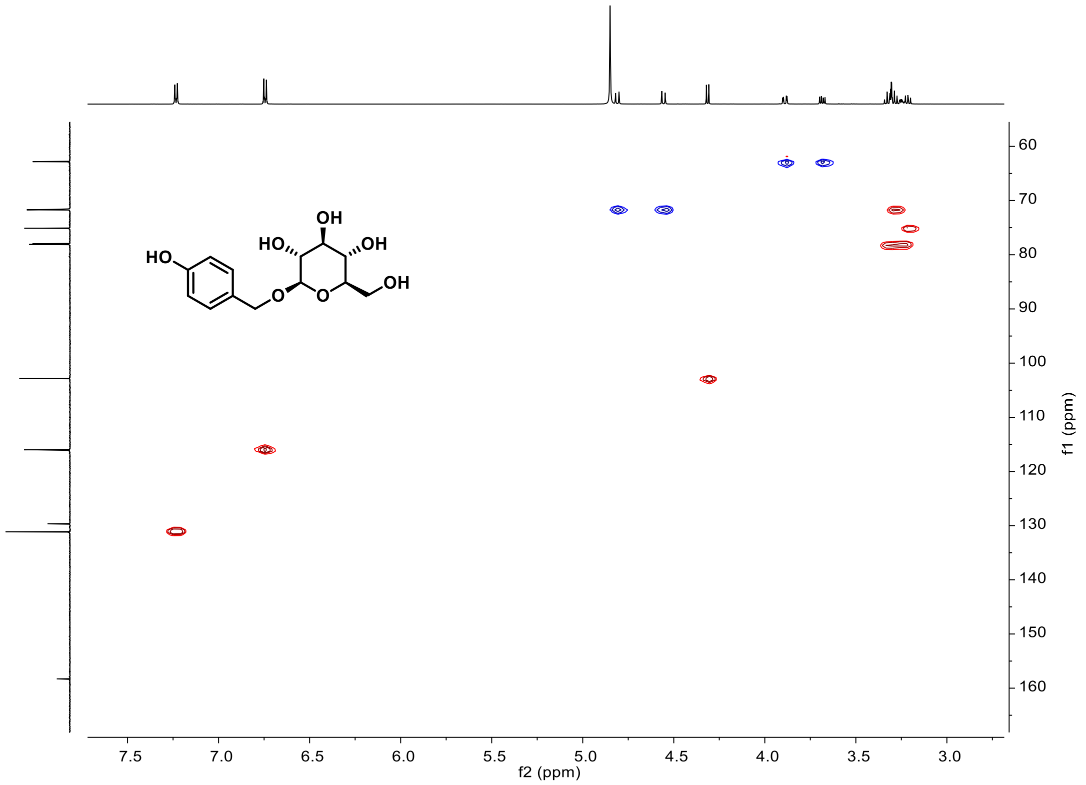

Fig. S90. HSQC NMR spectrum of 1b in CD_3OD.

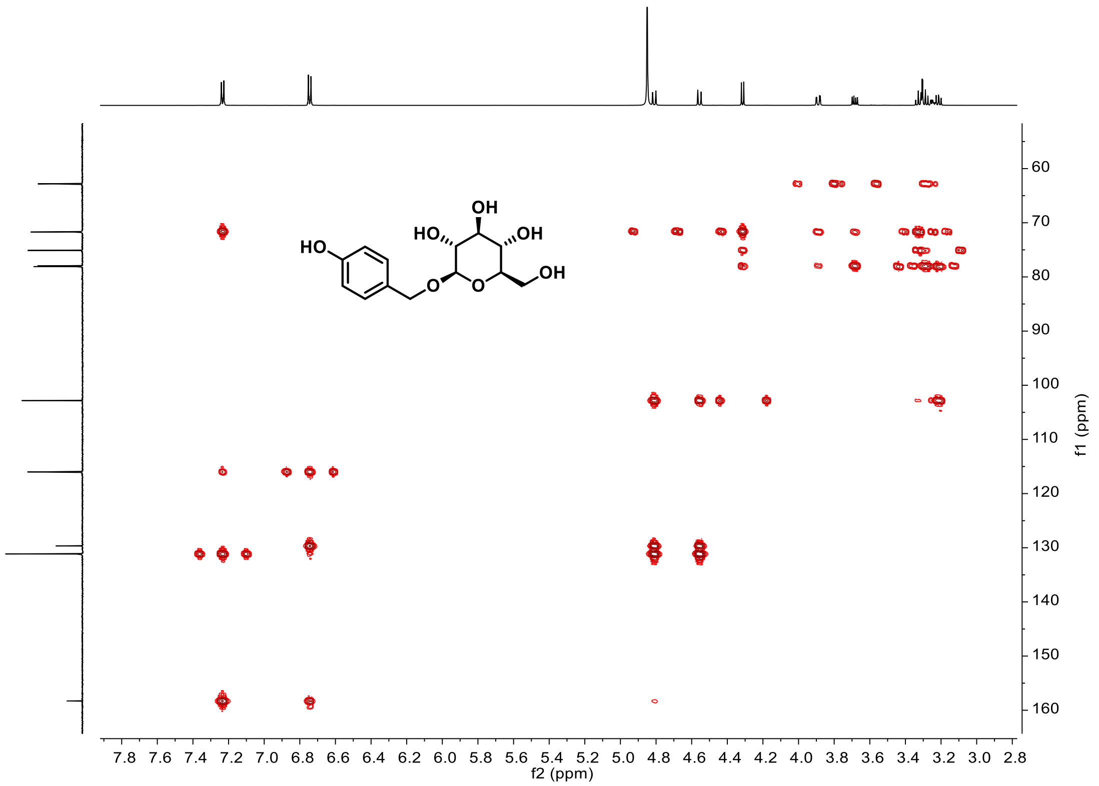

Fig. S91. HMBC NMR spectrum of 1b in CD3OD.

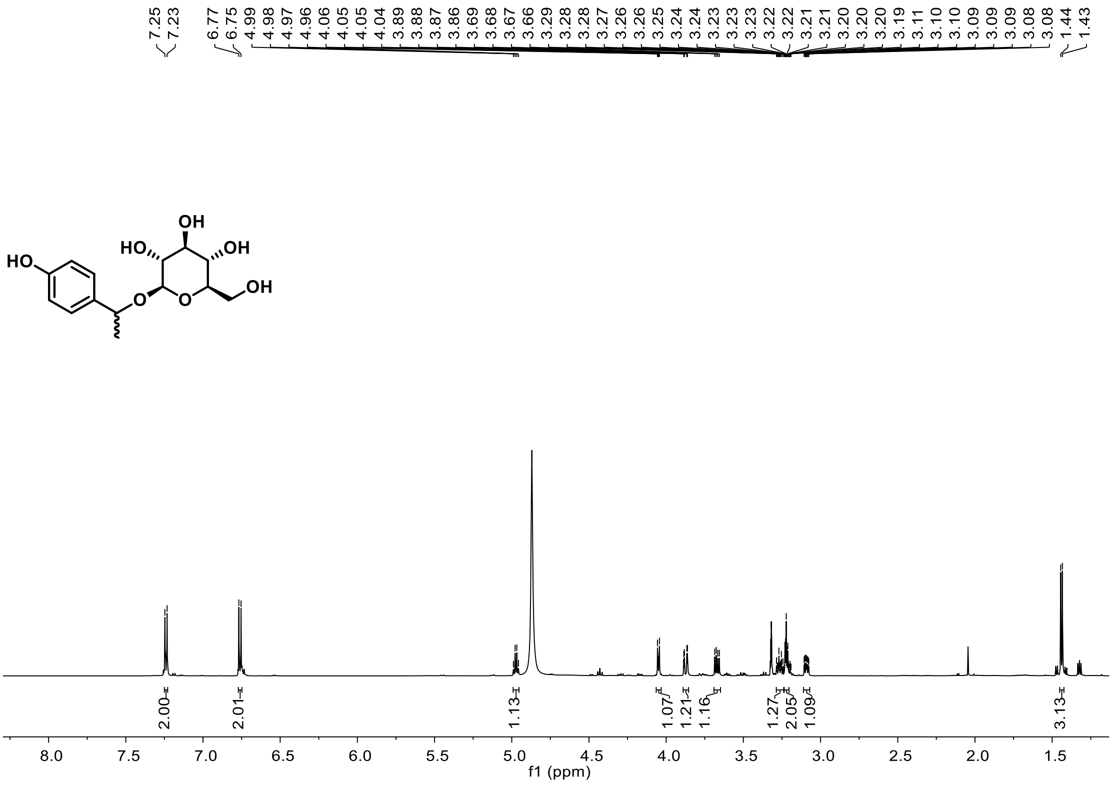

Fig. S92. ^{1}H NMR spectrum of 13b in CD3OD.

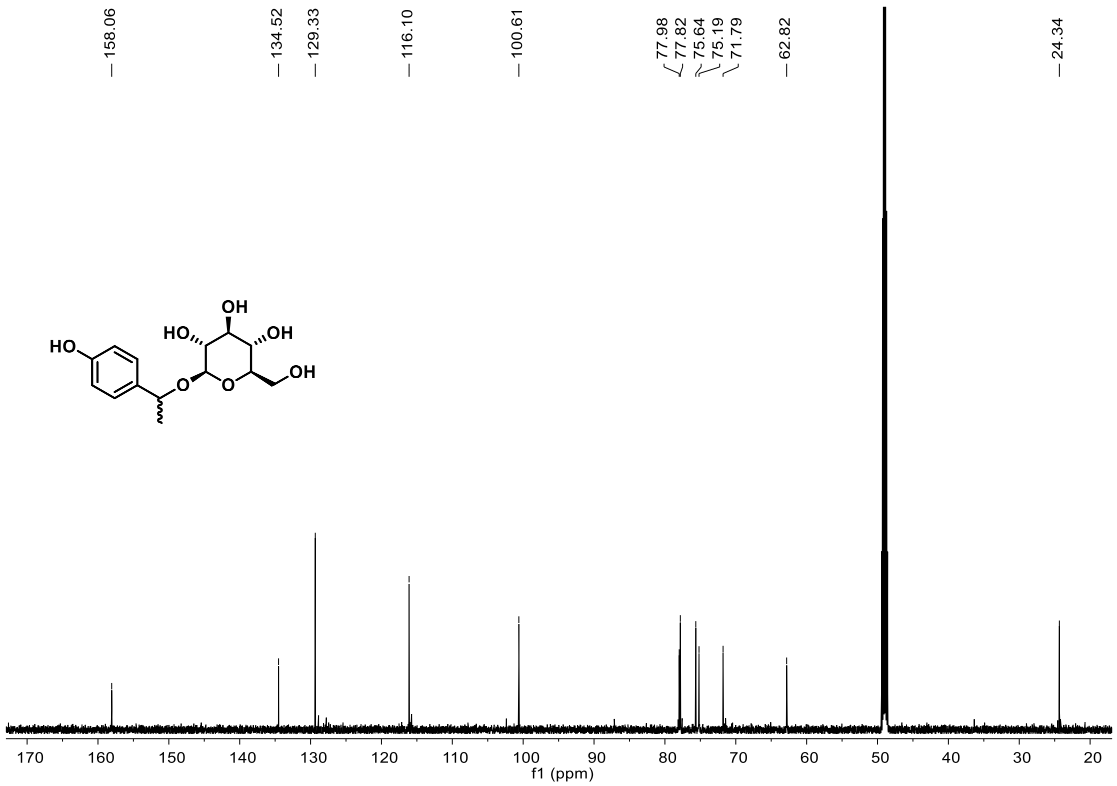

Fig. S93. ^{13}C NMR spectrum of 13b in CD_3OD.

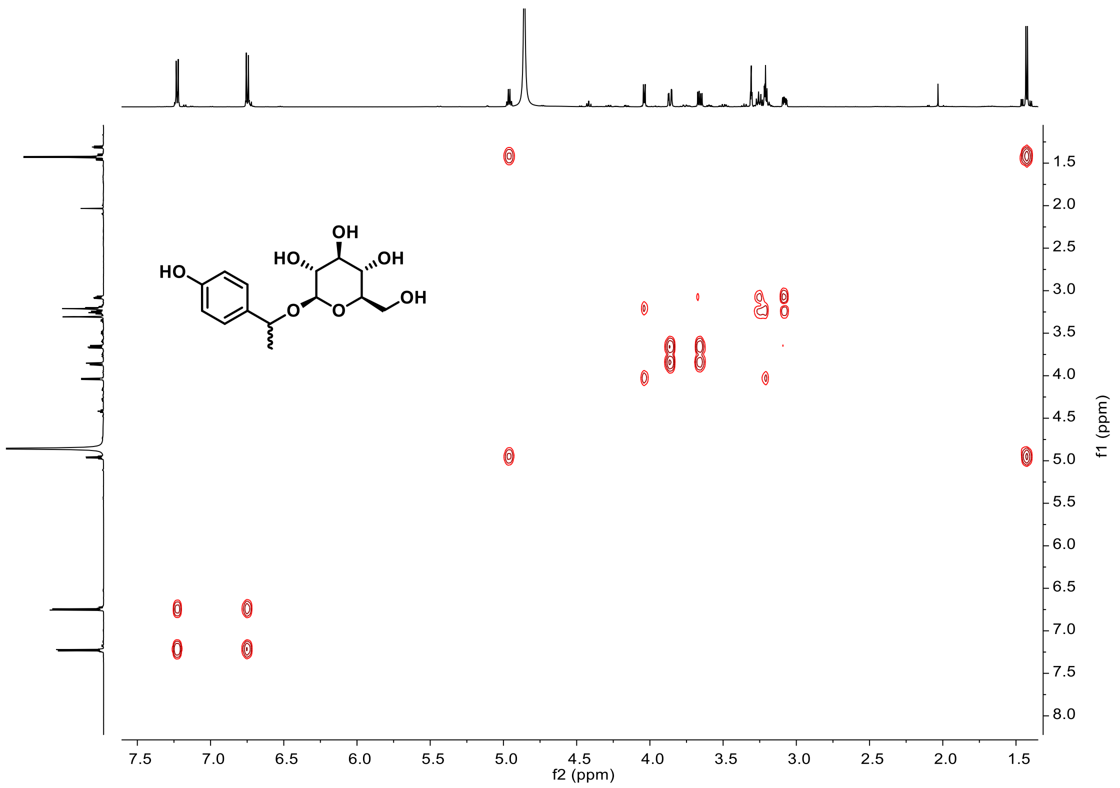

Fig. S94. COSY NMR spectrum of 13b in CD_3OD.

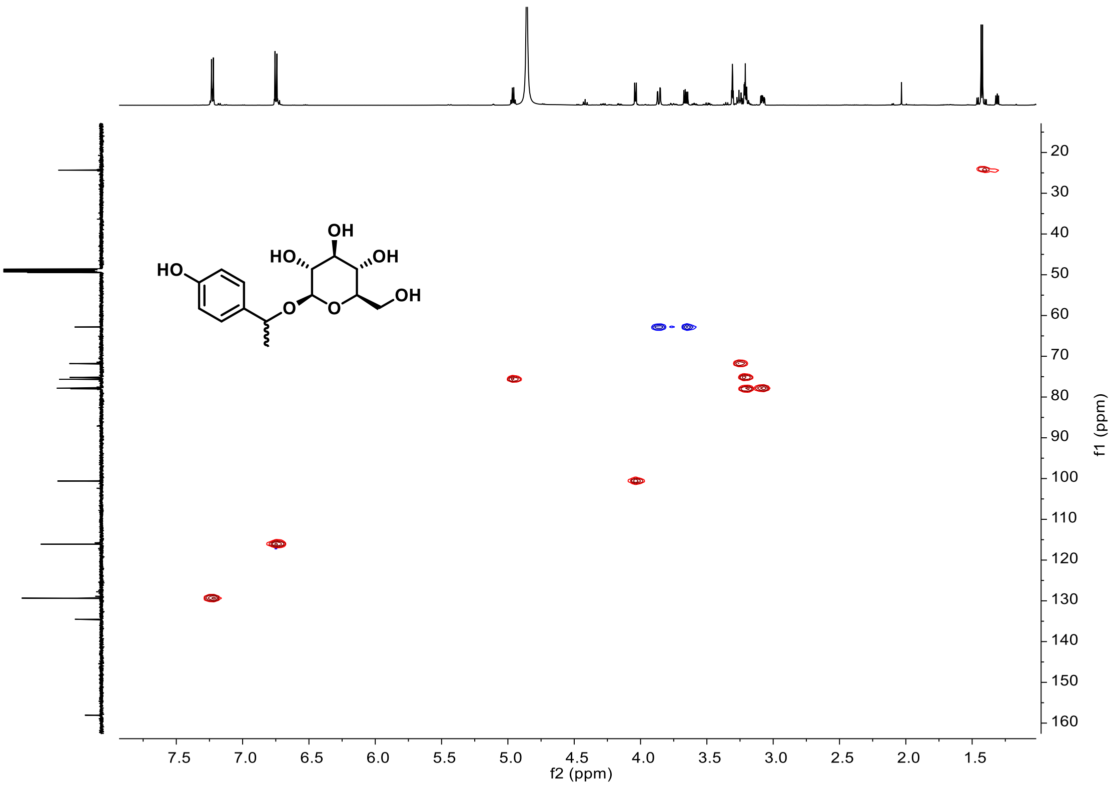

Fig. S95. HSQC NMR spectrum of 13b in CD_3OD.

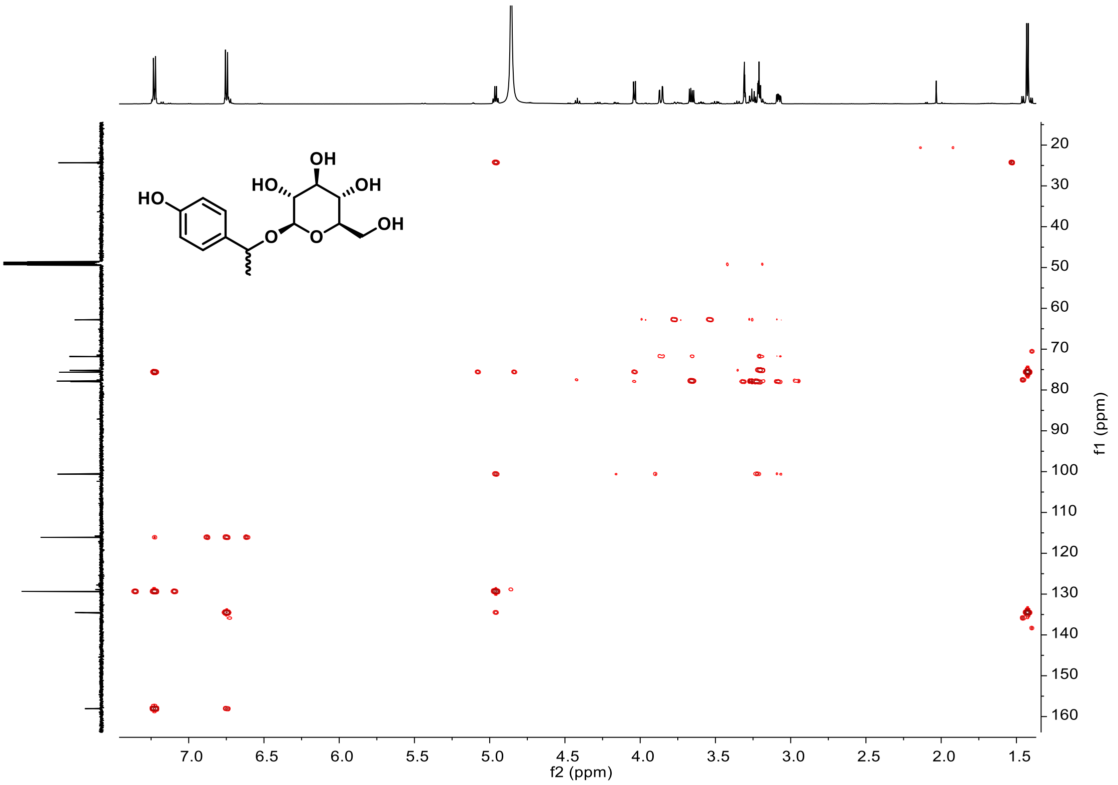

Fig. S96. HMBC NMR spectrum of 13b in CD_3OD.

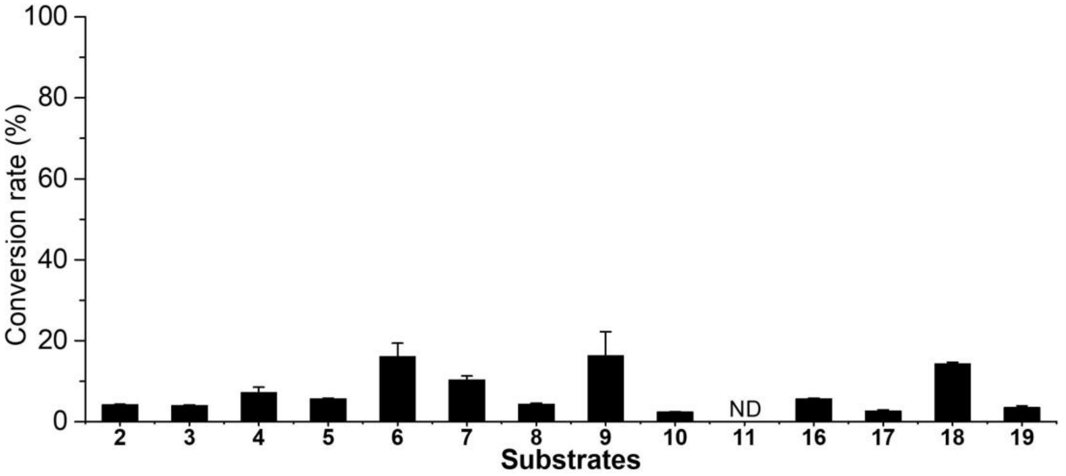

Fig. S97. Enzymatic assays of UGT85A1 towards substrates with only one phenolic hydroxyl group.

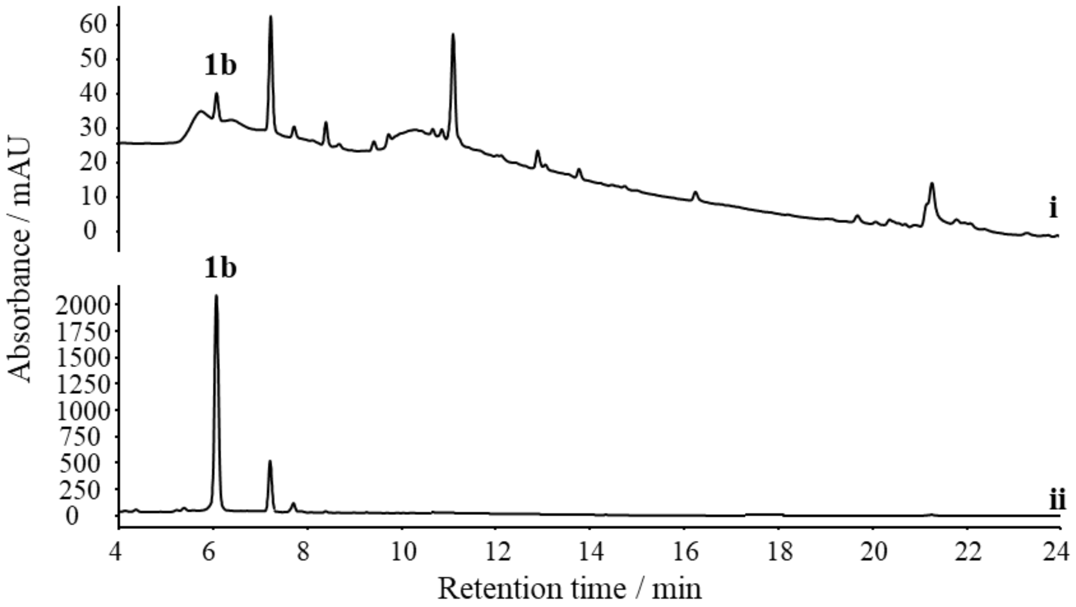

Fig. S98. HPLC Analysis of adsorption capacity of two macroporous resins S-8 (i) and AB-8 (ii) to 1b. Adsorption yields of glycosides with S-8 and AB-8 are calculated as 0.13% and 43.78%, respectively.

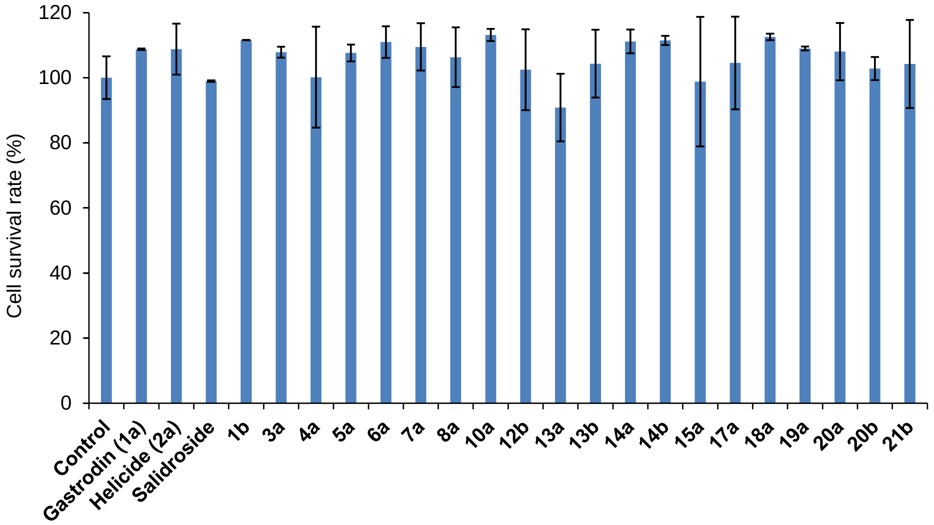

Fig. S99. Cytotoxicity determination of glucosides.

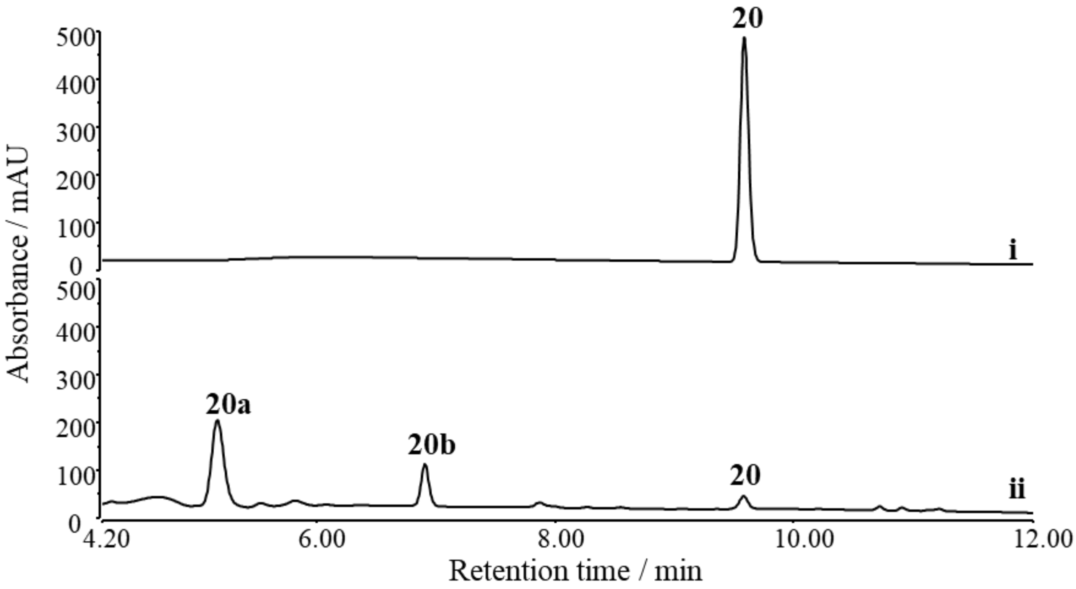

Fig. S100. Transformation of substrate 20 by *E. coli* BL21(DE3) containing UG73B6FS. i, standard substrate **20**; ii, biotransformation. The conversion efficiency is 92.52%. Product **20a** accounted for 66.55%, and product **20b** accounted for 25.97%.

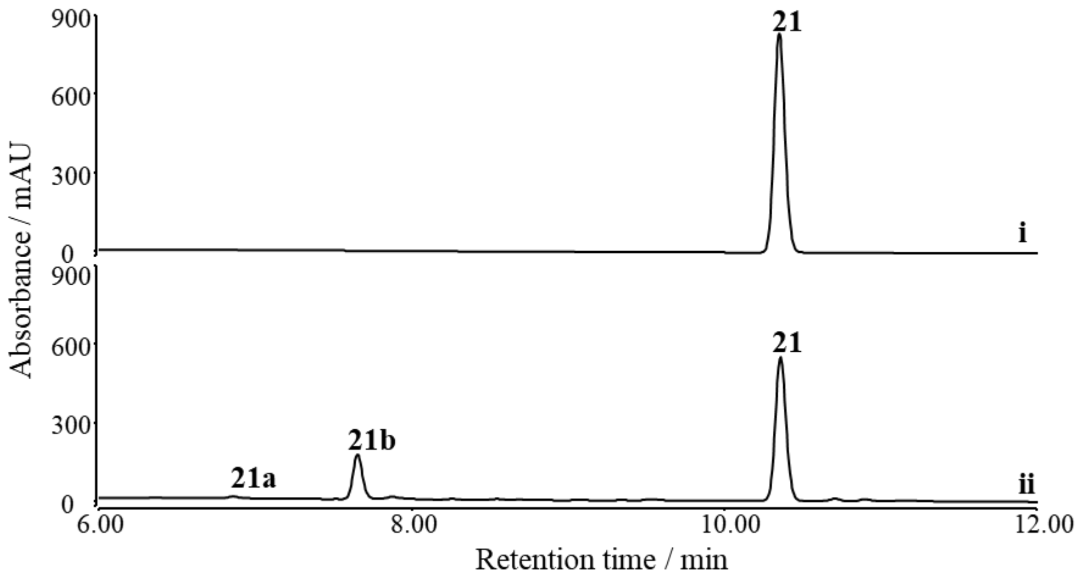

Fig. S101. Transformation of substrate 21 by *E. coli* BL21(DE3) containing UG73B6FS. i, standard substrate **21**; ii, biotransformation. The conversion efficiency is 34.92%. Product **21a** accounted for 8.03%, and product **21b** accounted for 26.89%.

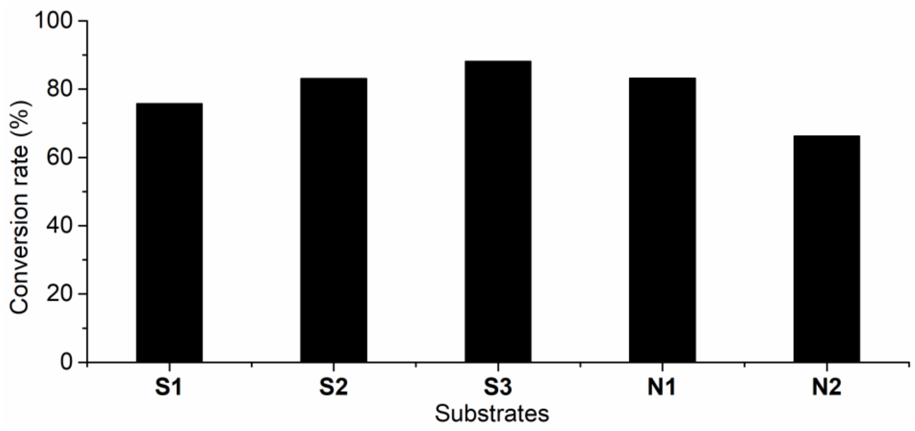

Fig. S102. Enzymatic assays of RrUGT3 towards the substrates with a sulfhydryl group or an amino group.

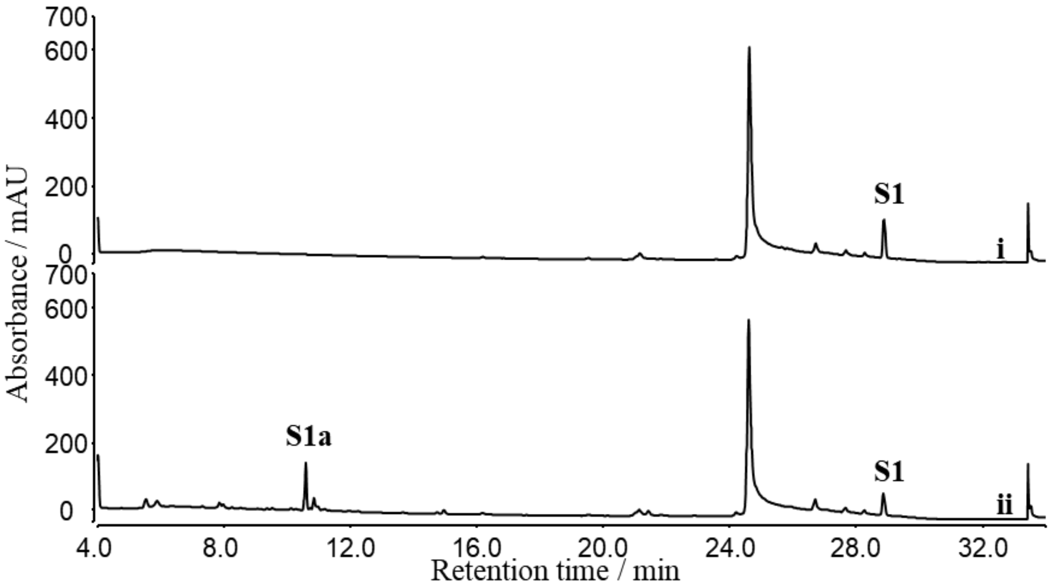

Fig. S103. Transformation of substrate S1 by *E. coli* BL21(DE3) containing RrUGT3. i, standard substrate **S1**; ii, biotransformation. The conversion efficiency is 36.45%.

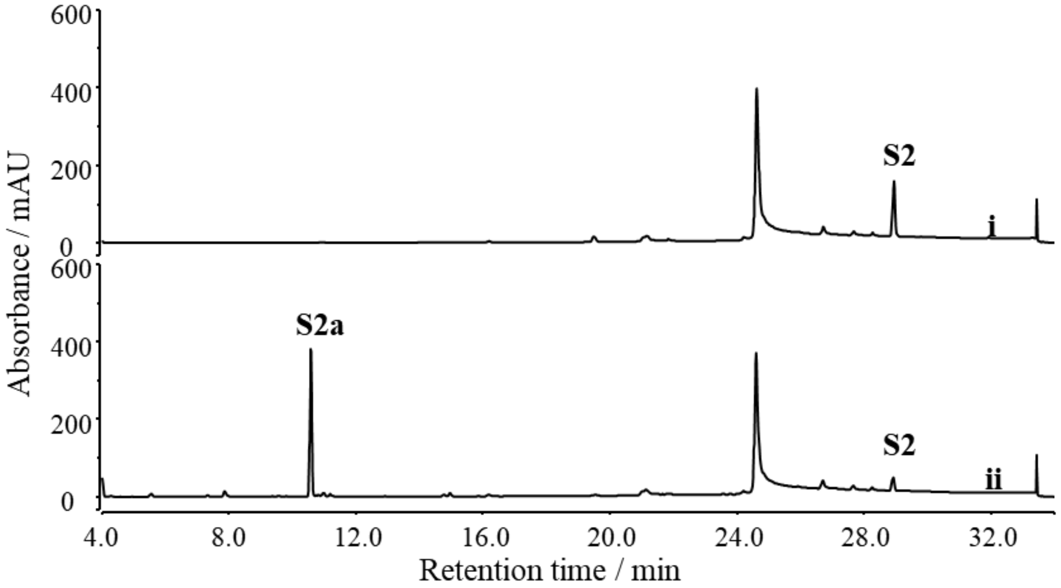

Fig. S104. Transformation of substrate S2 by *E. coli* BL21(DE3) containing RrUGT3. i, standard substrate **S2**; ii, biotransformation. The conversion efficiency is 81.18%.

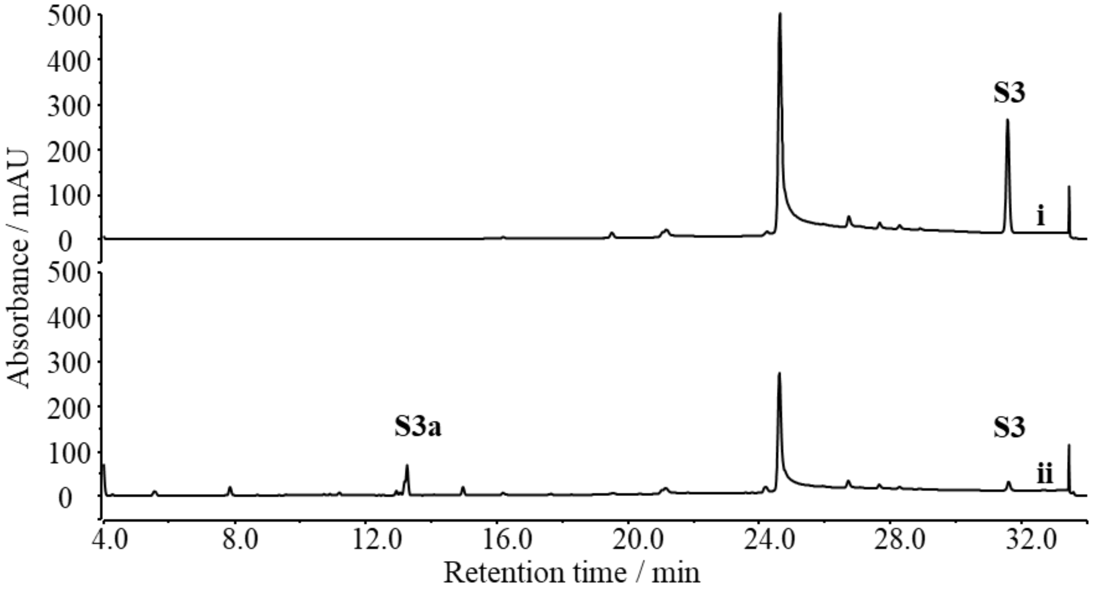

Fig. S105. Transformation of substrate S3 by *E. coli* BL21(DE3) expressing RrUGT3. i, standard substrate **S3**; ii, biotransformation. The conversion ratio is 93.5%.

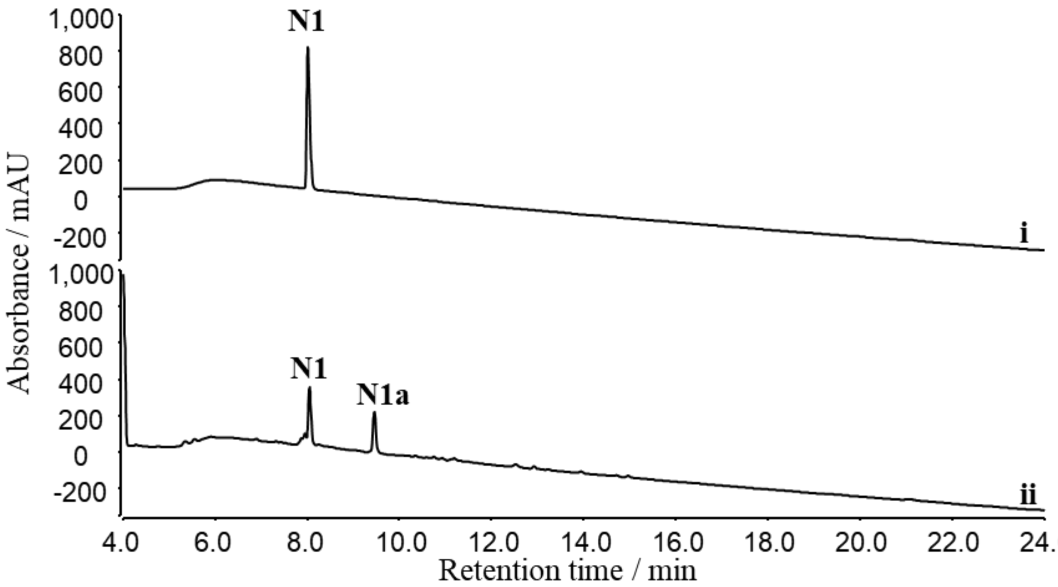

Fig. S106. Transformation of substrate N1 by *E. coli* BL21(DE3) containing RrUGT3. i, standard substrate **N1**; ii, biotransformation. The conversion efficiency is 58.16%.

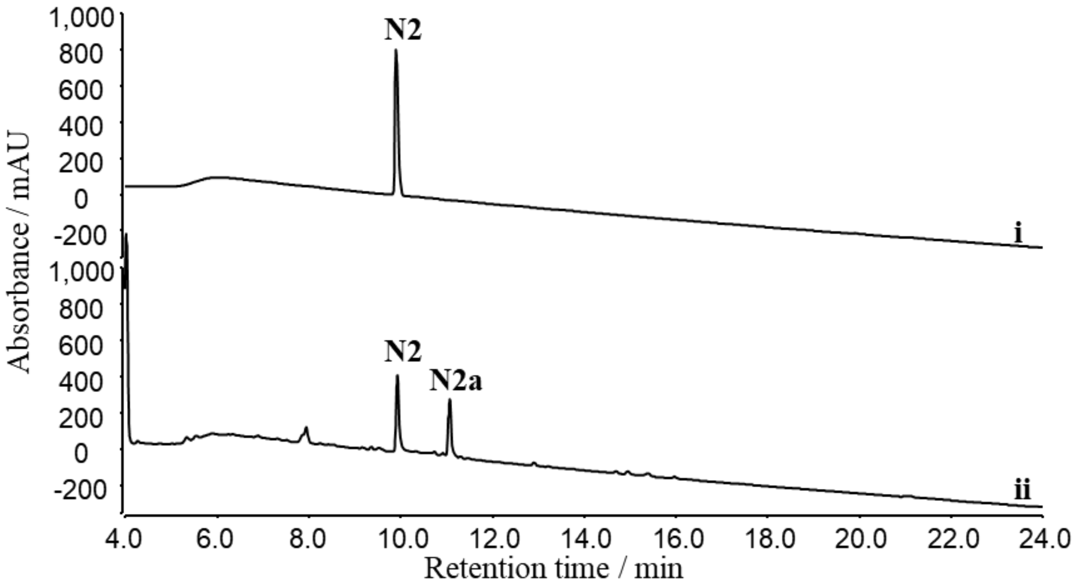

Fig. S107. Transformation of substrate N2 by *E. coli* BL21(DE3) containing RrUGT3. i, standard substrate **N2**; ii, biotransformation. The conversion efficiency is 51.49%.

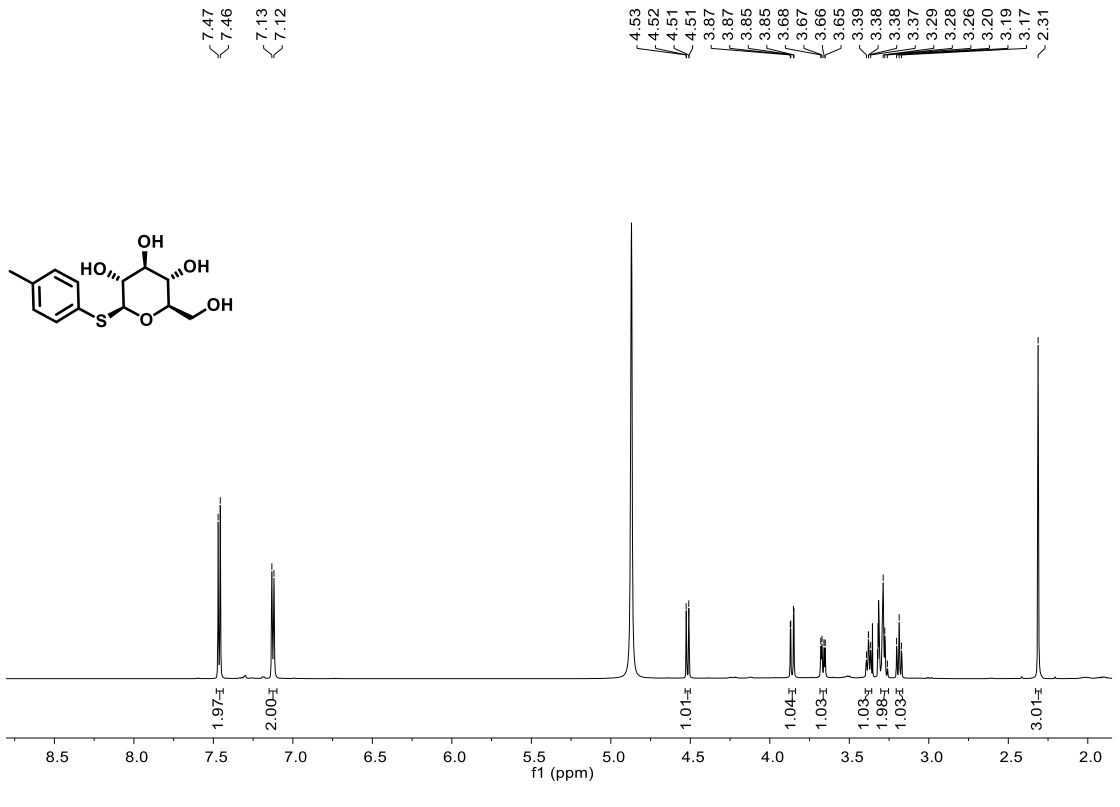

Fig. S108. ^{1}H NMR spectrum of S1a in CD3OD.

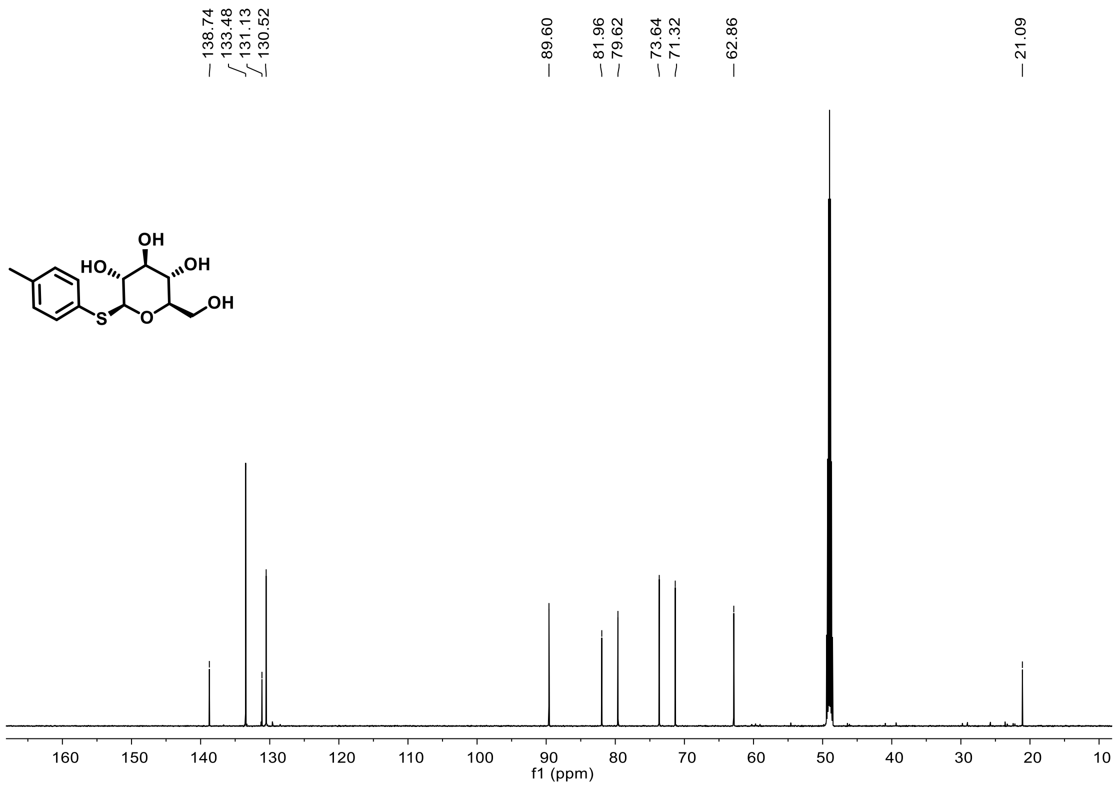

Fig. S109. ^{13}C NMR spectrum of S1a in CD$_3$OD.

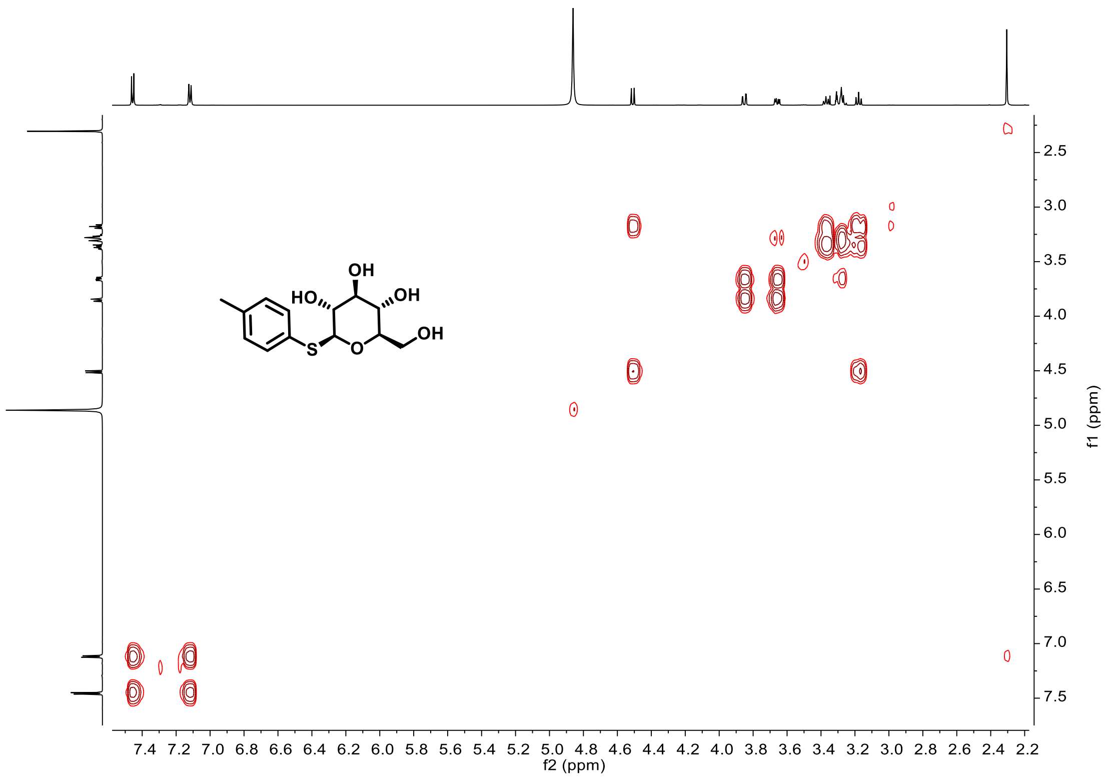

Fig. S110. COSY NMR spectrum of S1a in CD$_3$OD.

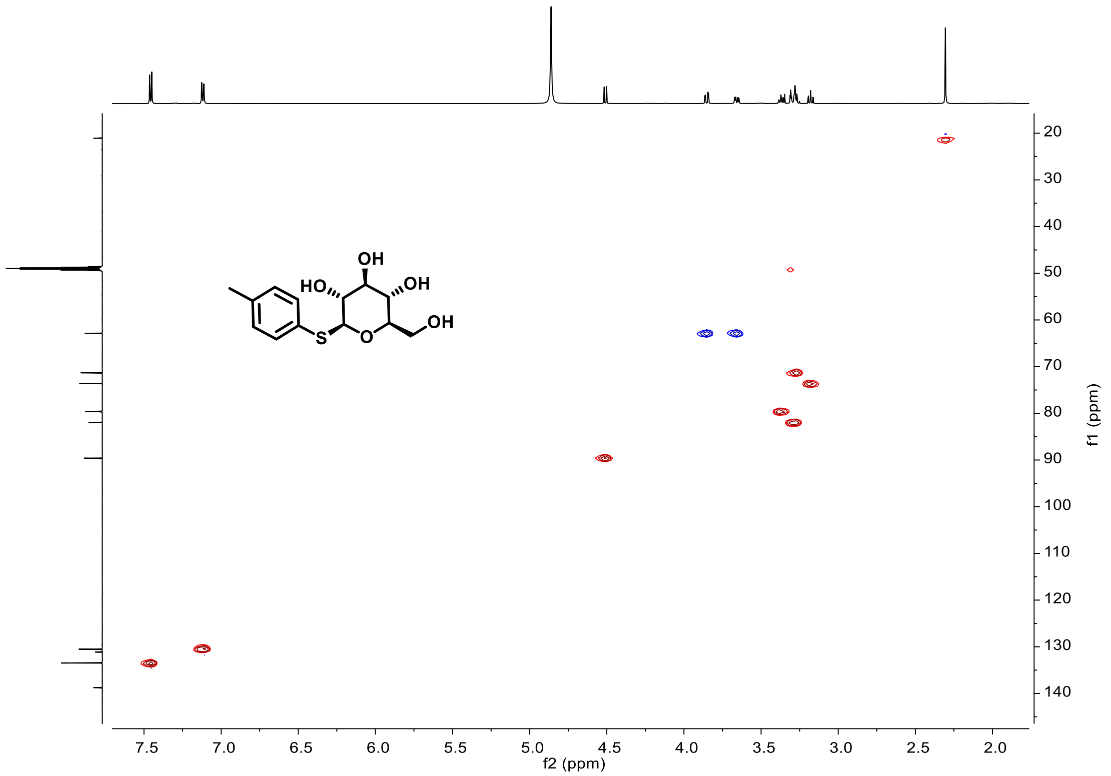

Fig. S111. HSQC NMR spectrum of S1a in CD_3OD.

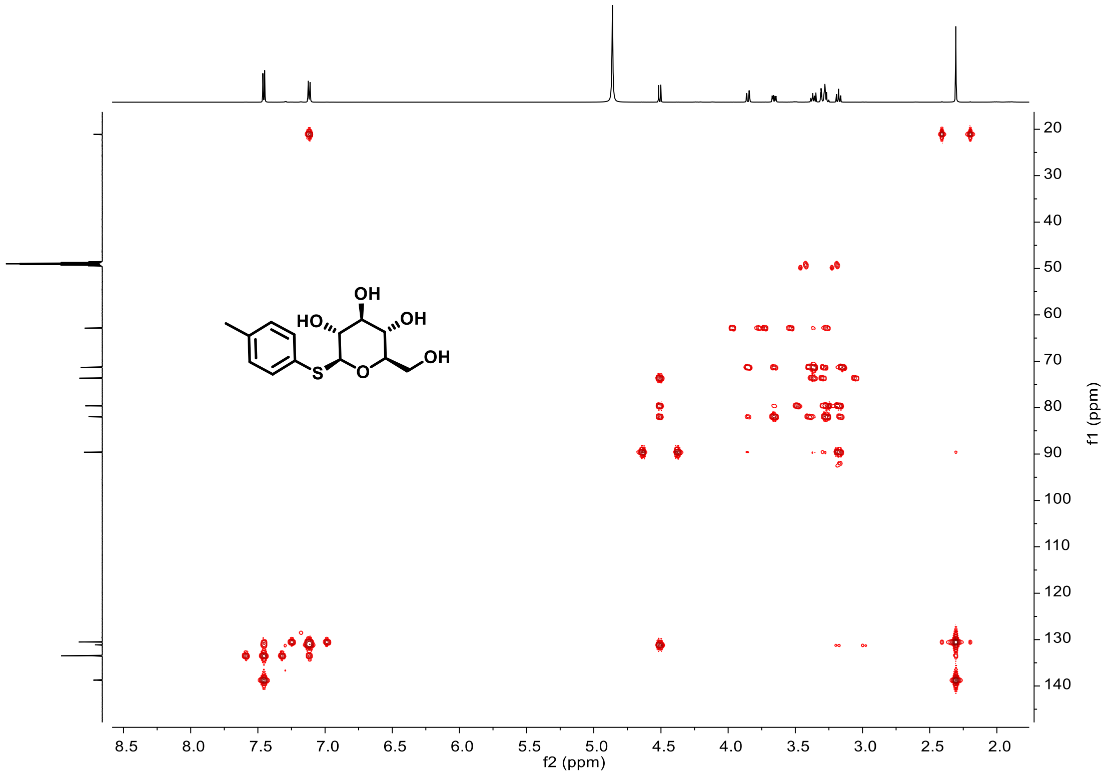

Fig. S112. HMBC NMR spectrum of S1a in CD_3OD.

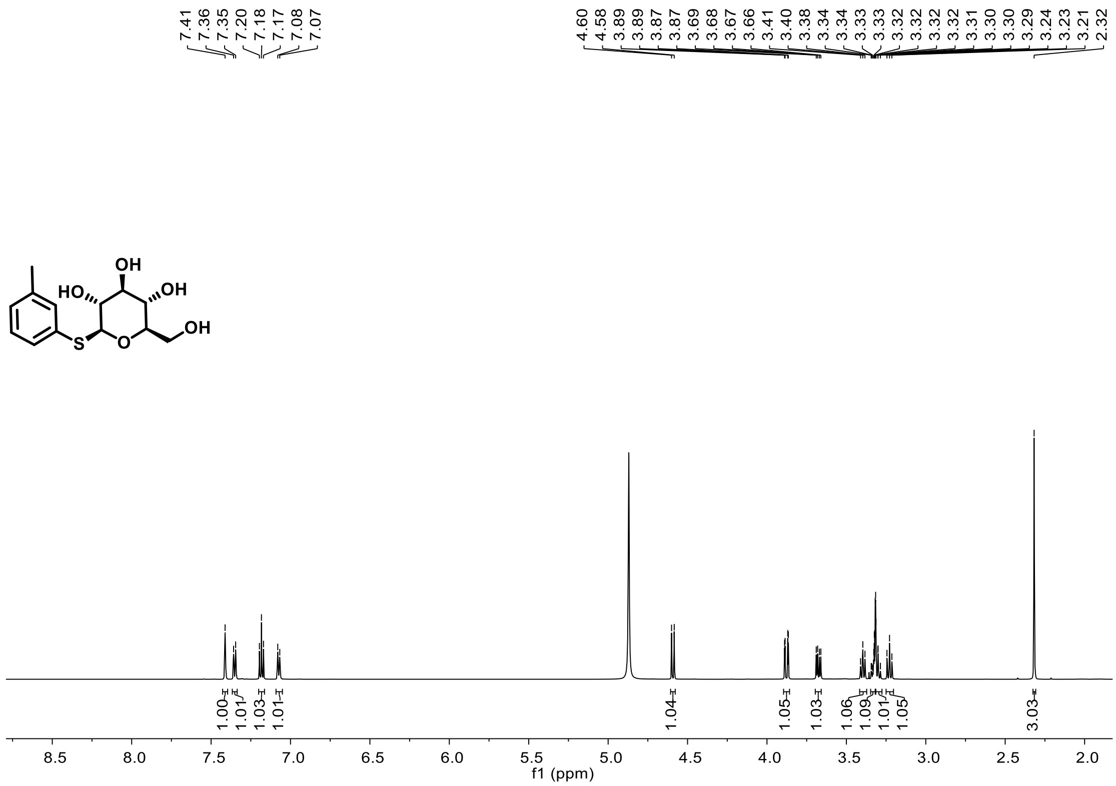

Fig. S113. ^{1}H NMR spectrum of S2a in CD_3OD.

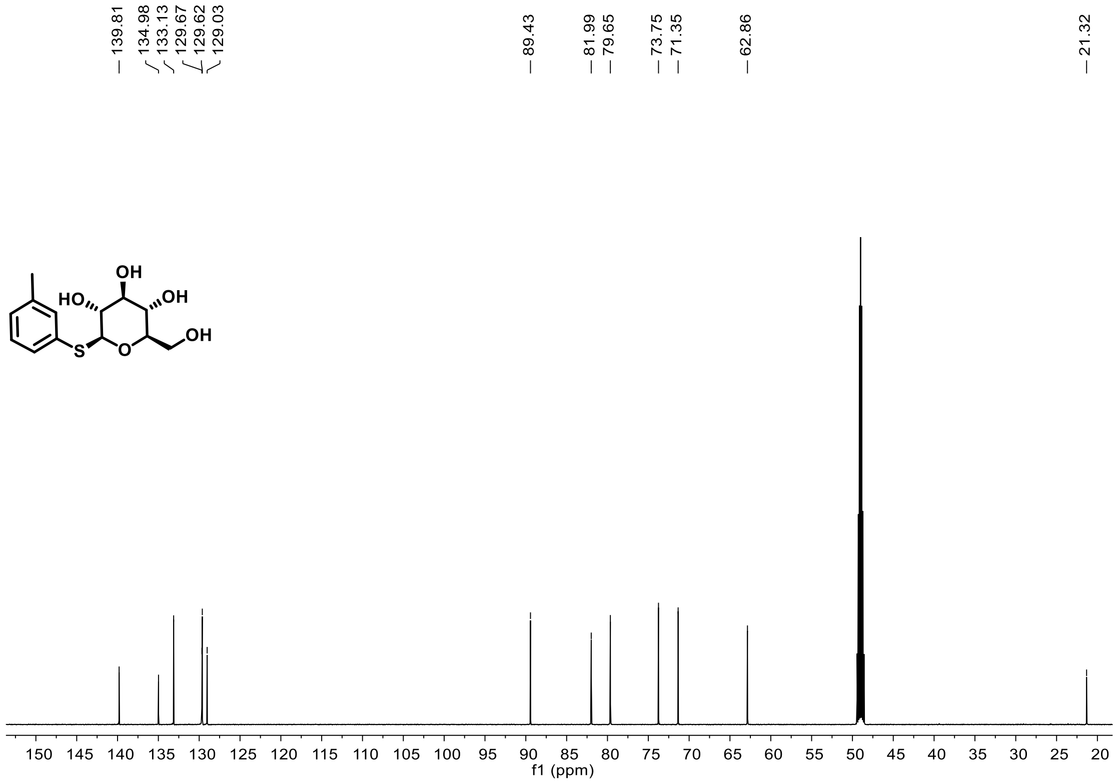

Fig. S114. ^{13}C NMR spectrum of S2a in CD_3OD.

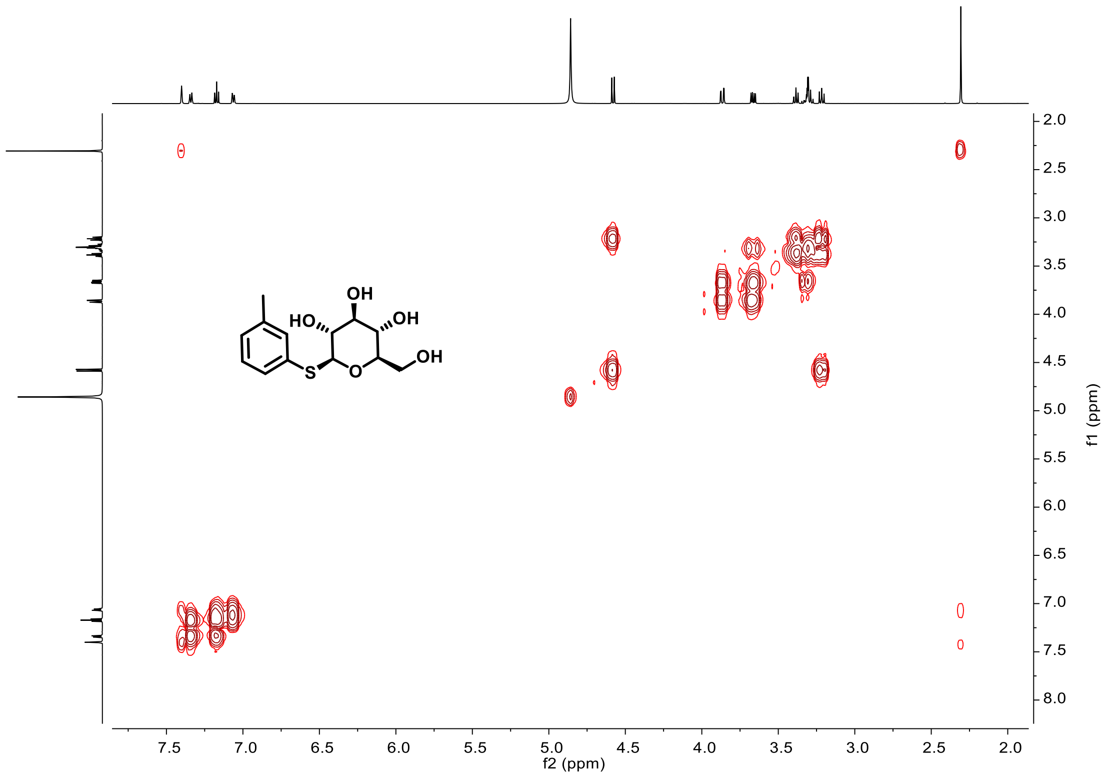

Fig. S115. COSY NMR spectrum of S2a in CD3OD.

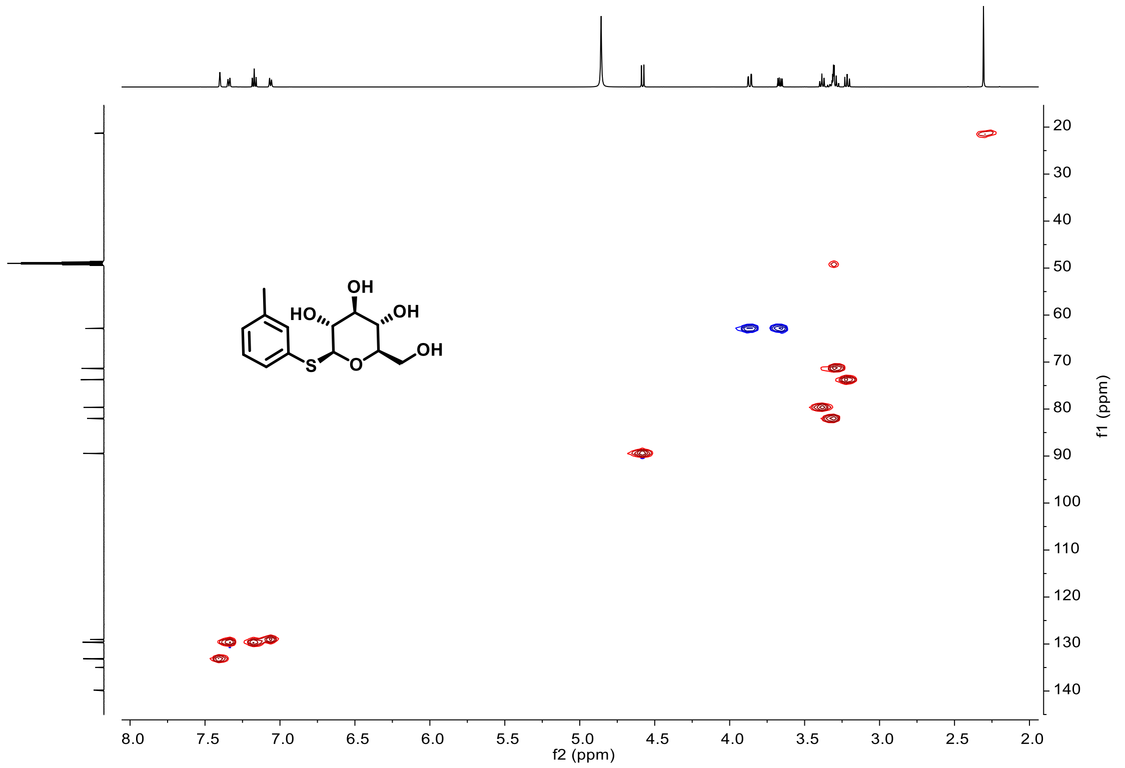

Fig. S116. HSQC NMR spectrum of S2a in CD3OD.

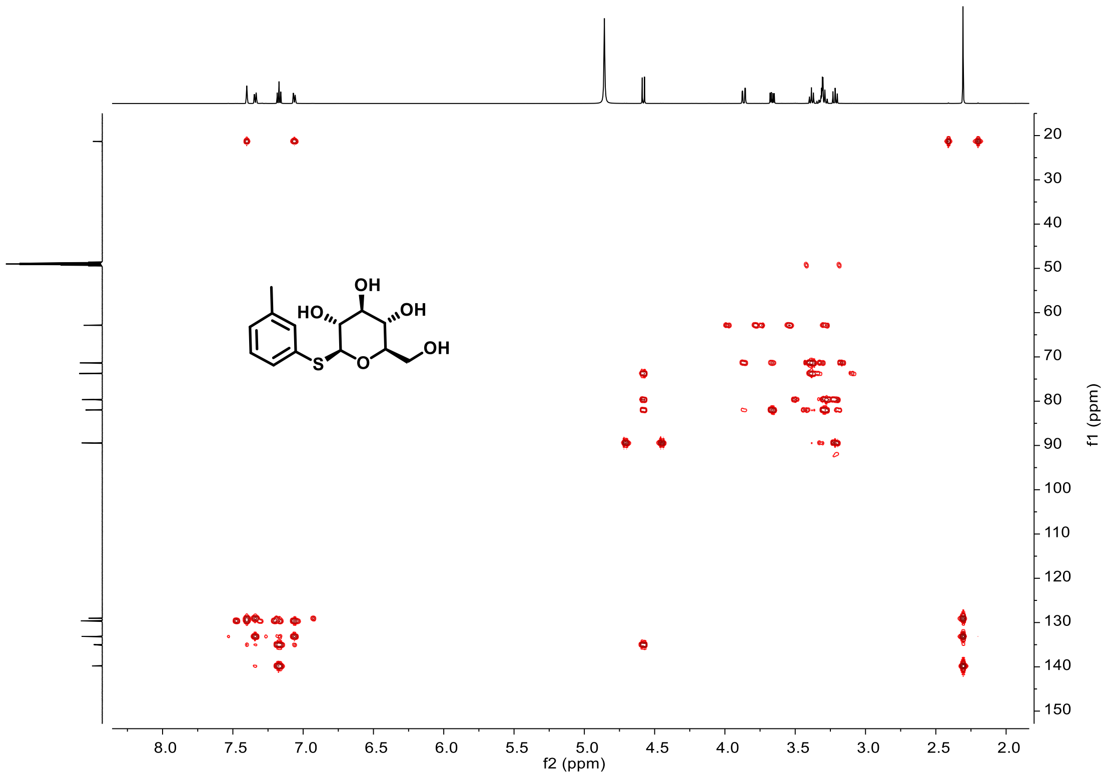

Fig. S117. HMBC NMR spectrum of S2a in CD3OD.

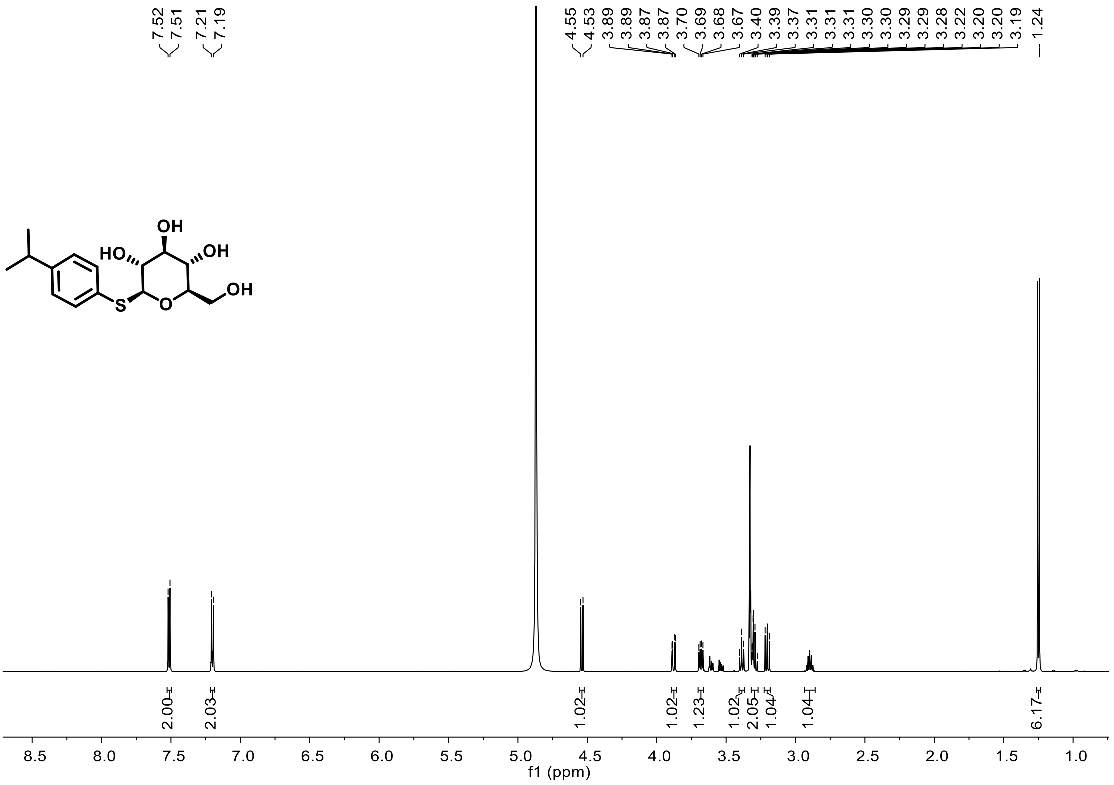

Fig. S118. ^{1}H NMR spectrum of S3a in CD3OD.

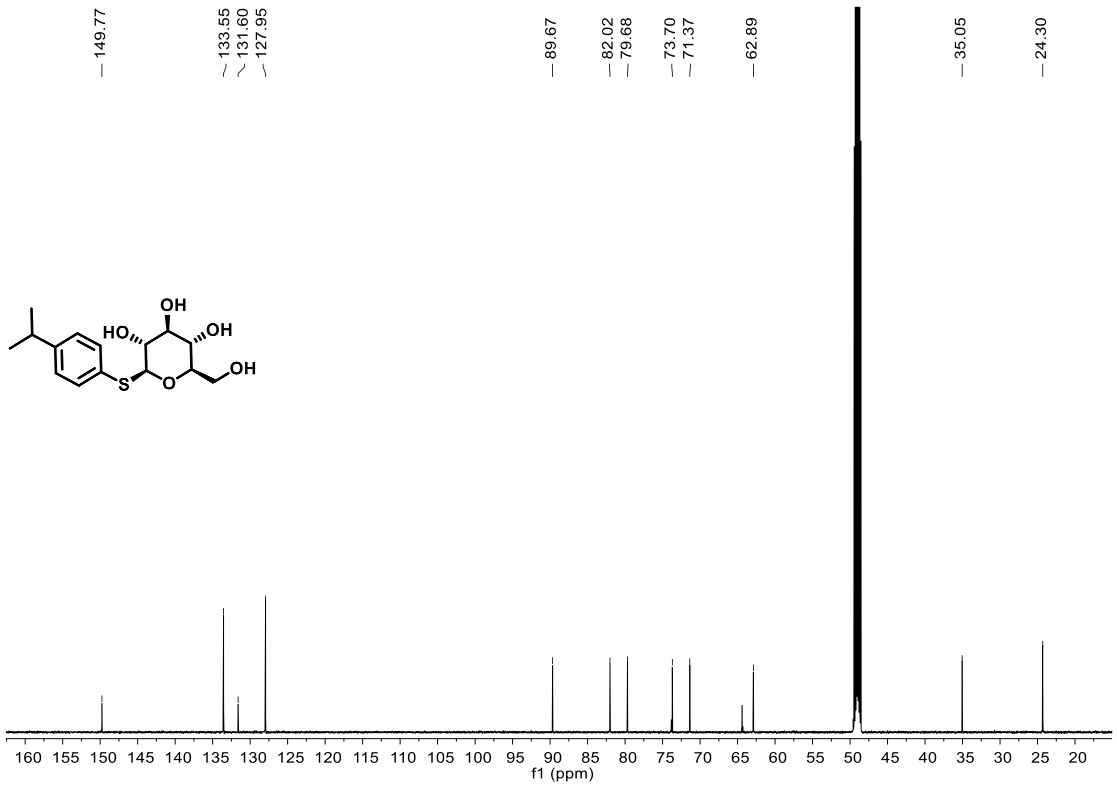

Fig. S119. ^{13}C NMR spectrum of S3a in CD3OD.

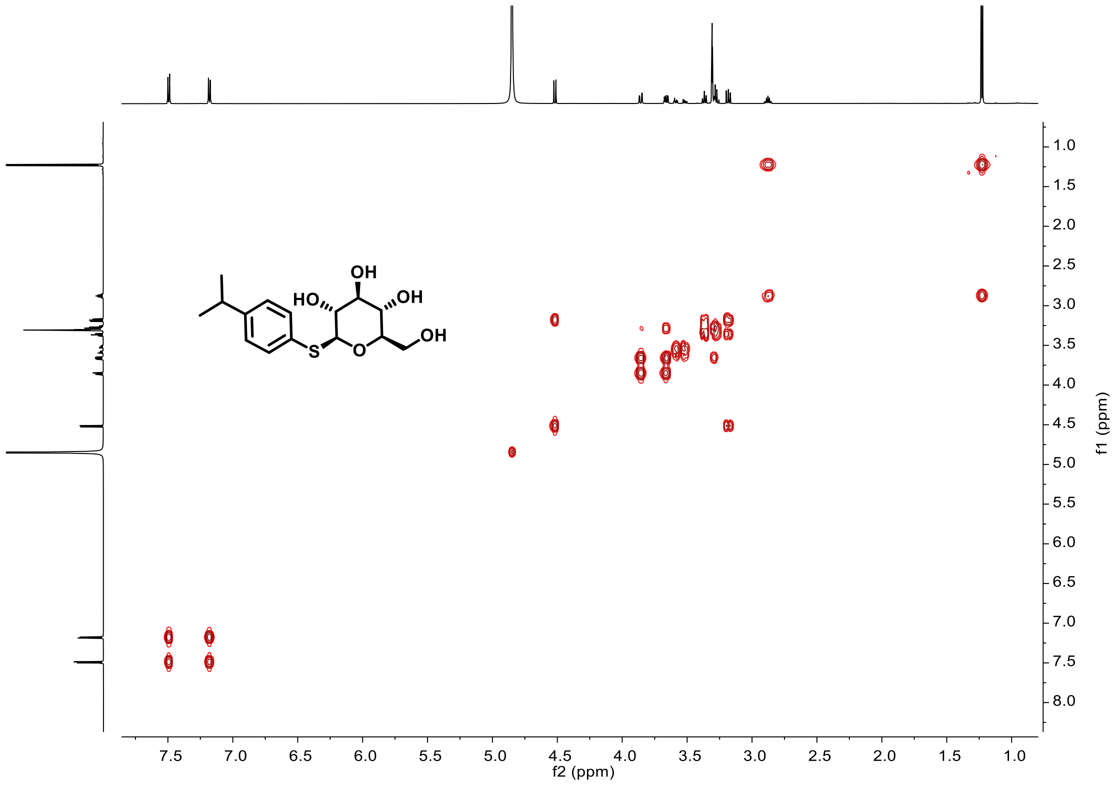

Fig. S120. COSY NMR spectrum of S3a in CD3OD.

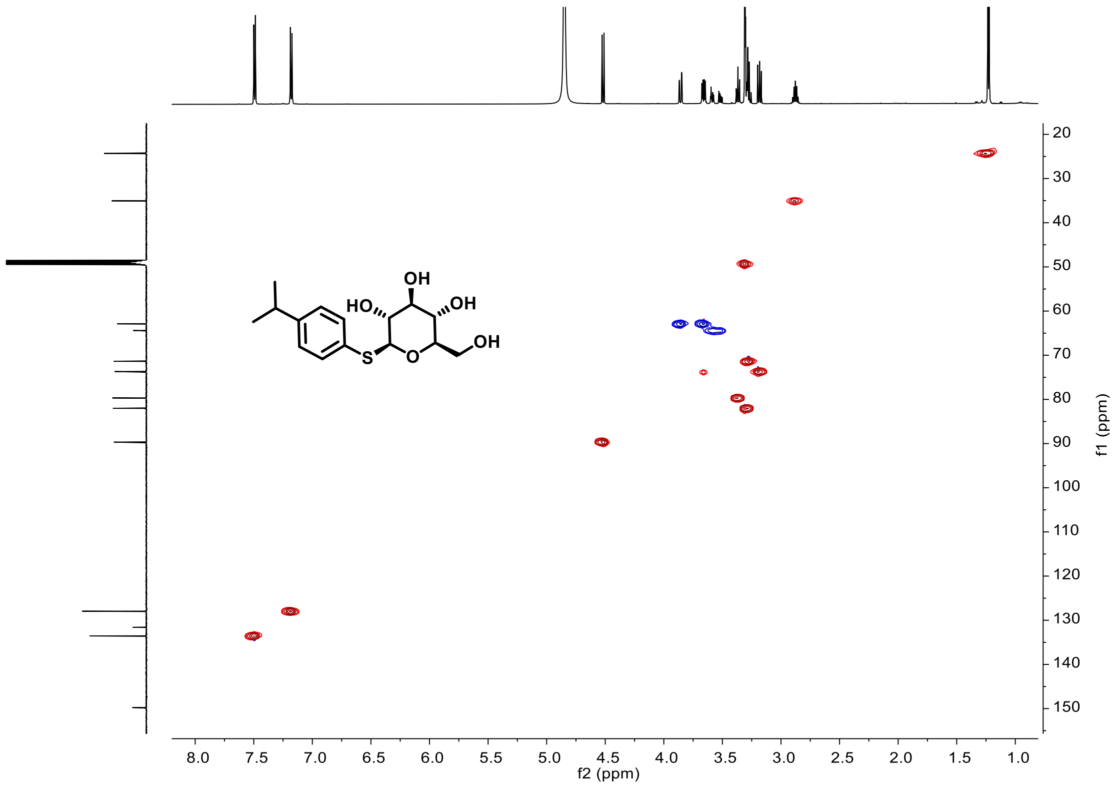

Fig. S121. HSQC NMR spectrum of S3a in CD_3OD.

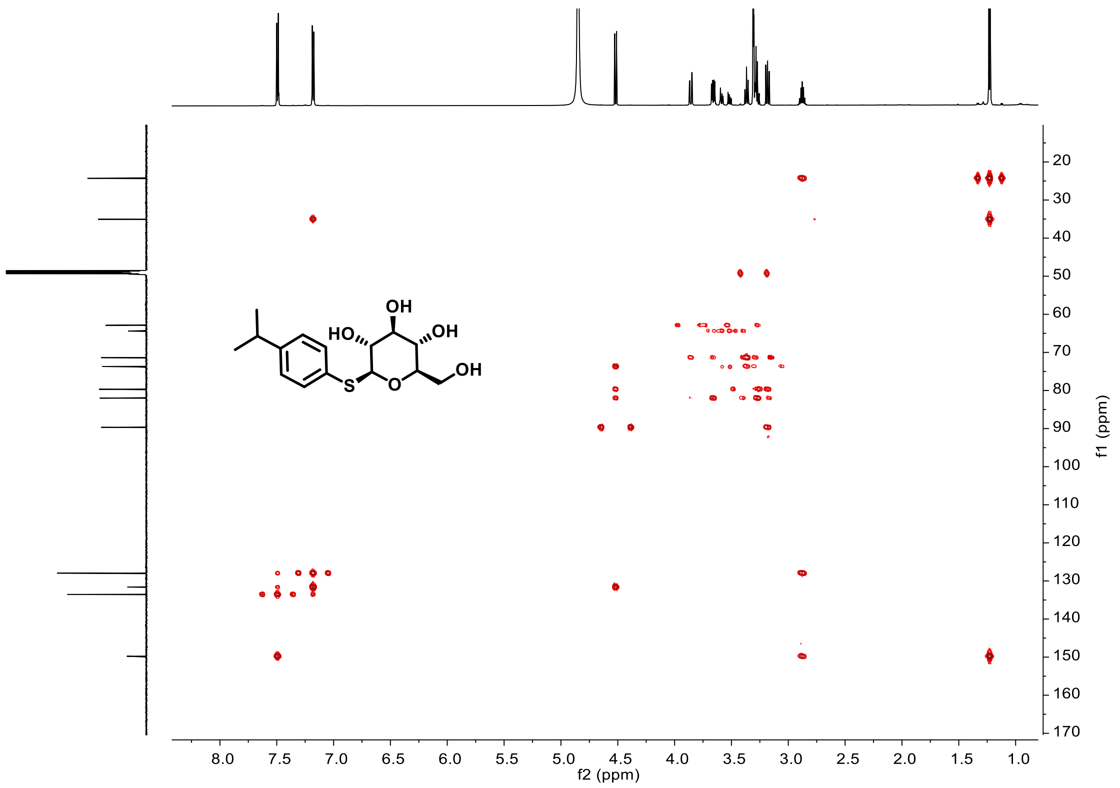

Fig. S122. HMBC NMR spectrum of S3a in CD_3OD.

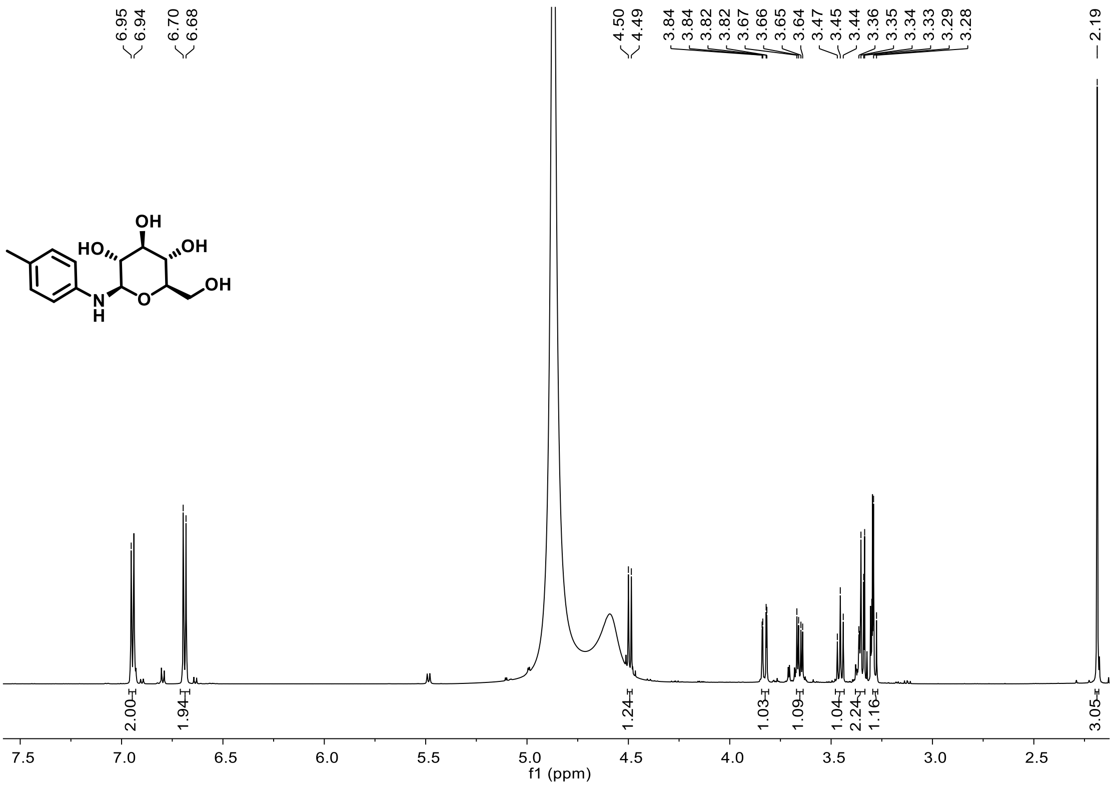

Fig. S123. ¹H NMR spectrum of N1a in CD₃OD.

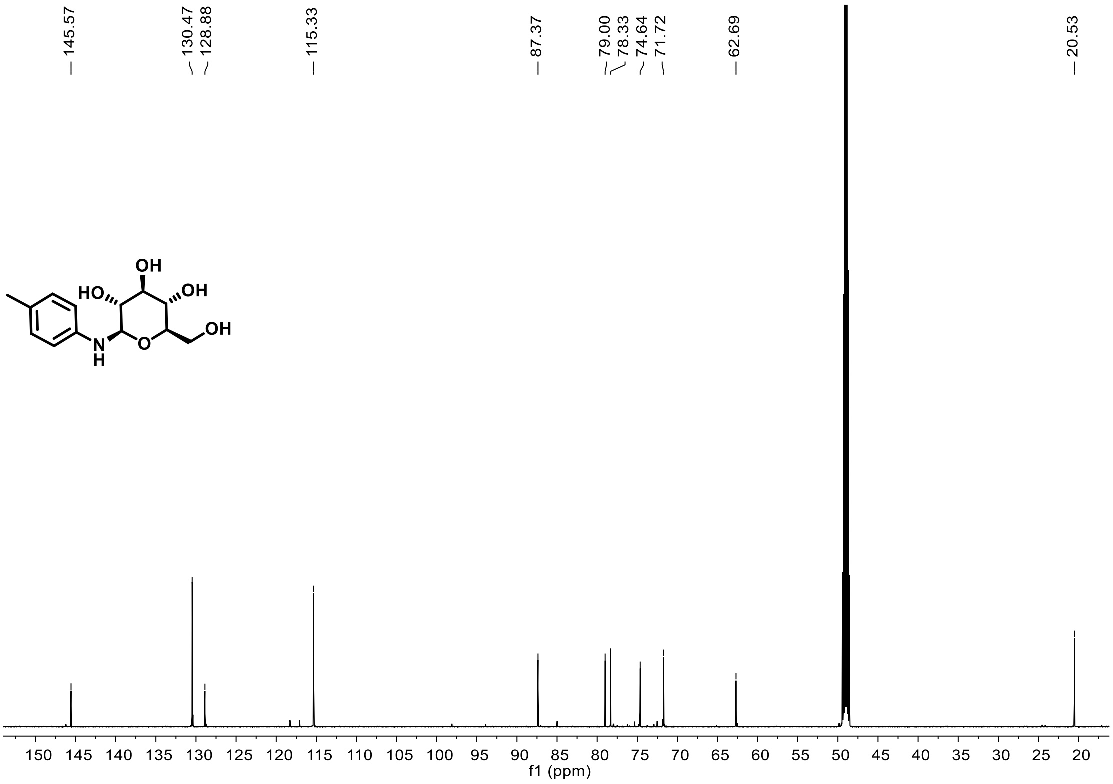

Fig. S124. ¹³C NMR spectrum of N1a in CD₃OD.

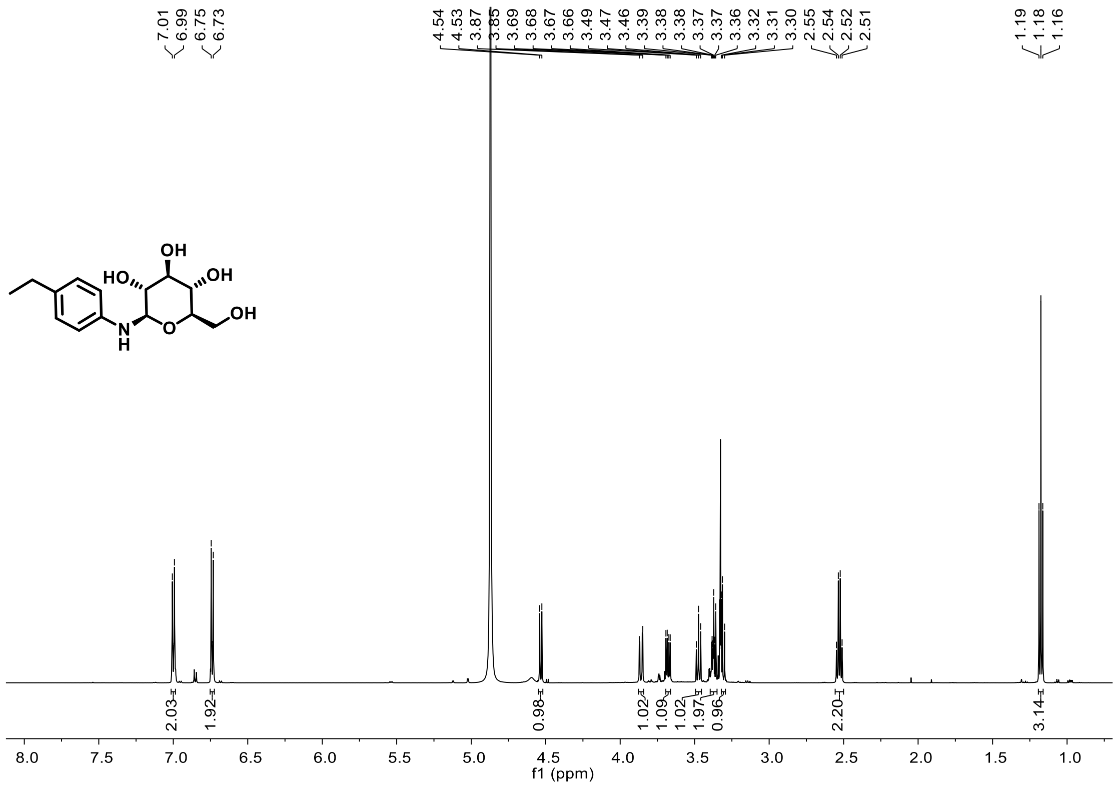

Fig. S125. ^{1}H NMR spectrum of N2a in CD_3OD.

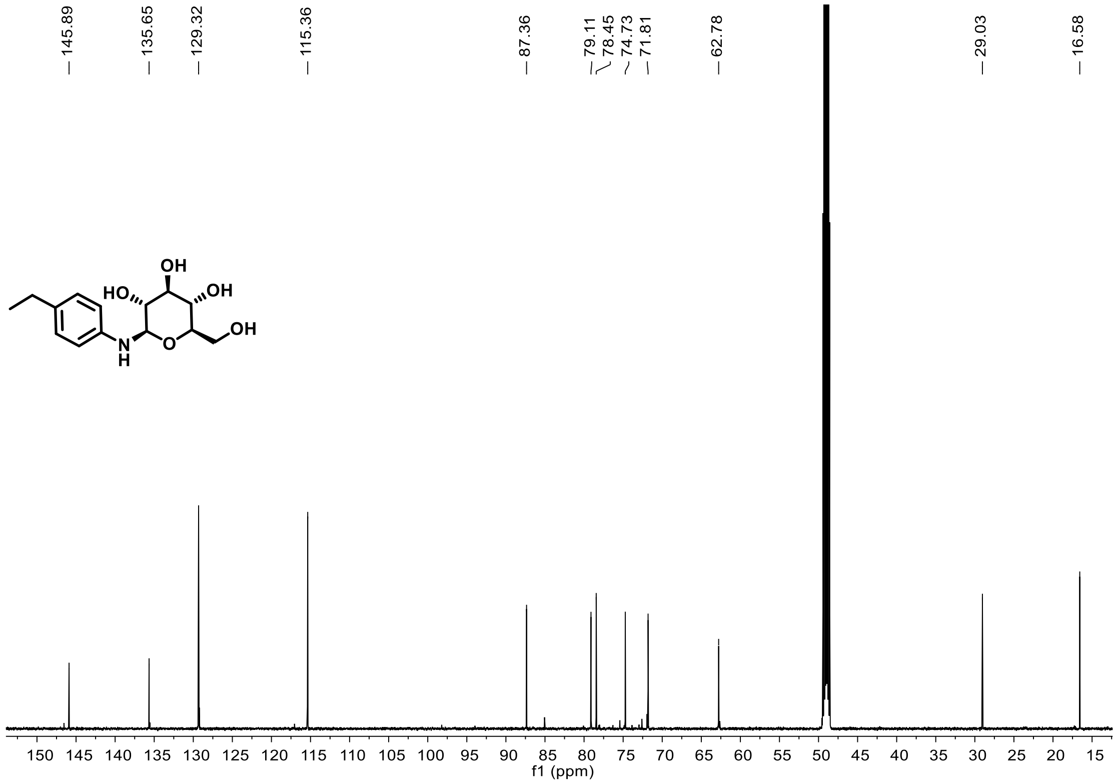

Fig. S126. ^{13}C NMR spectrum of N2a in CD_3OD.

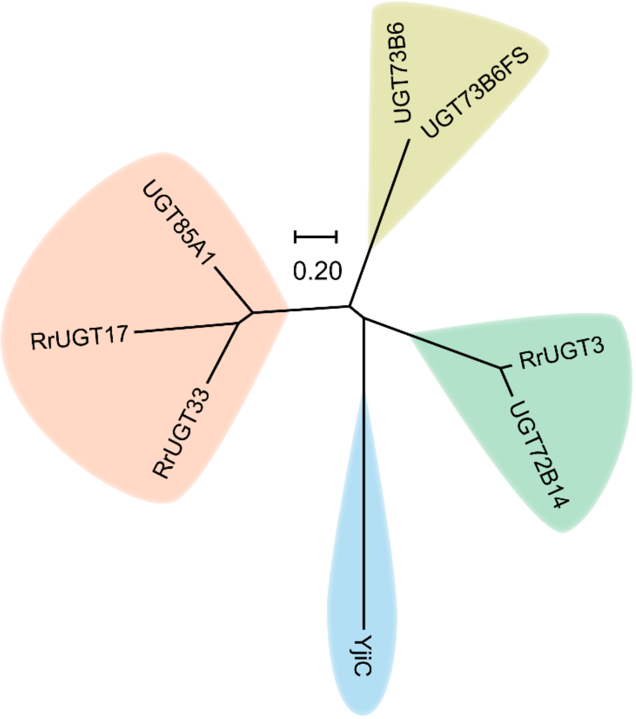

Fig. S127. Phylogenetic analysis of UGTs. The evolutionary history is inferred by using the Maximum Likelihood method and JTT matrix-based model.

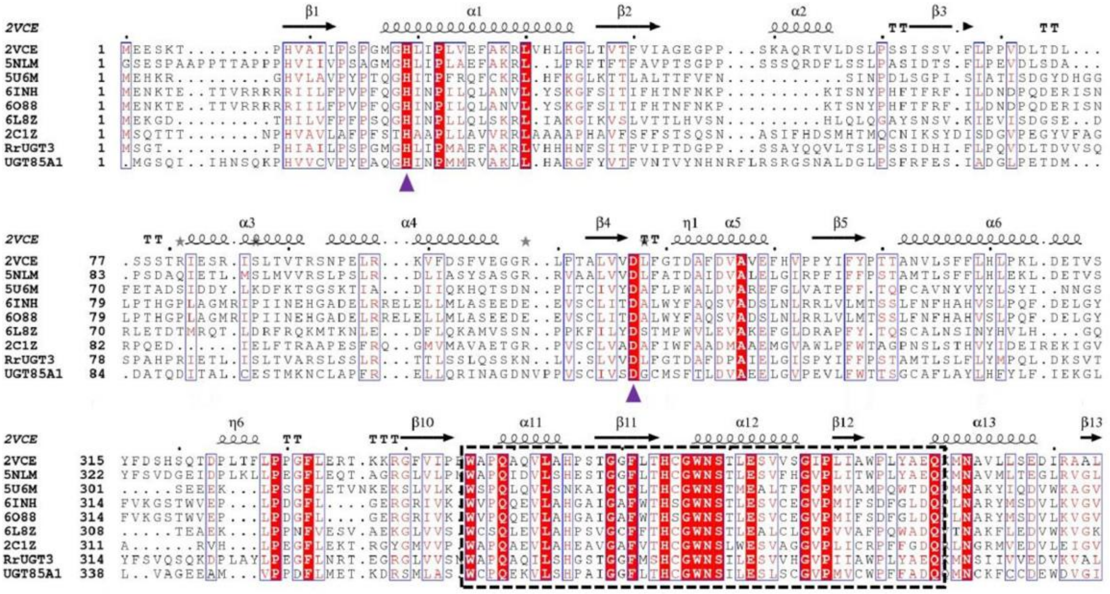

Fig. S128. Sequence alignment of RrUGT3, UGT85A1 and the homologous UGTs. Red backgrounds and blue rectangles indicate sequence identity and similarity, respectively. The catalytic dyad residues are marked with purple triangles. The PSPG motif is boxed in black dashed rectangles.

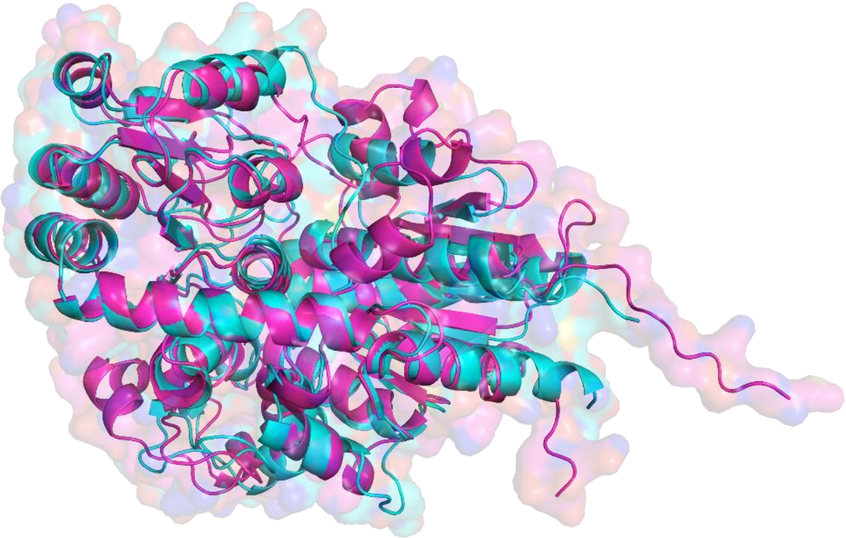

Fig. S129. Comparison of the AlphaFold2-simulated structures of RrUGT3 (cyan) and UGT85A1 (magenta).

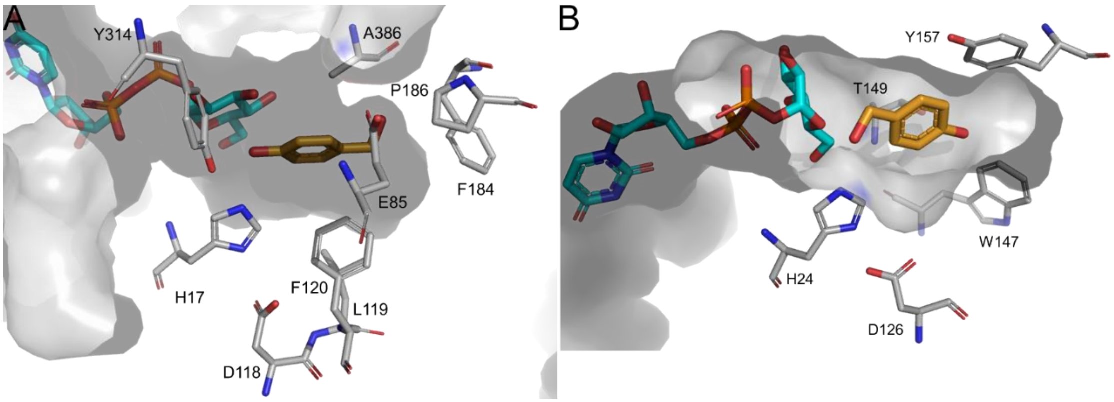

Fig. S130. The substrate docking results of RrUGT3 (A) and UGT85A1 (B). The residues forming the substrate binding pockets are shown in grey. The sugar donor and acceptor are colored in cyan and orange, respectively.

Reference

1. Wang J, Sui X, Ding Y, Fu Y, Feng X, Liu M, et al. A fast and robust iterative genome-editing method based on a Rock-Paper-Scissors strategy. Nucleic Acids Res. 2021;49:e12.
